# SGBS cells as a model of human adipocyte browning: A comprehensive comparative study with primary human white subcutaneous adipocytes

**DOI:** 10.1038/s41598-017-04369-2

**Published:** 2017-06-22

**Authors:** Chia Rou Yeo, Madhur Agrawal, Shawn Hoon, Asim Shabbir, Manu Kunaal Shrivastava, Shiqi Huang, Chin Meng Khoo, Vanna Chhay, M. Shabeer Yassin, E. Shyong Tai, Antonio Vidal-Puig, Sue-Anne Toh

**Affiliations:** 1 Department of Medicine, Yong Loo Lin School of Medicine, National University of Singapore, 117599 Singapore, Singapore; 2 Molecular Engineering Laboratory, Biomedical Sciences Institutes, A*Star, 138668 Singapore, Singapore; 3 Department of Surgery, National University Hospital, 119074 Singapore, Singapore; 4 Metabolic Research Laboratories, Institute of Metabolic Science, Addenbrooke’s Hospital, University of Cambridge, Cambridge, CB2 0QQ UK; 5 Food Science and Technology Program, Department of Chemistry, National University of Singapore, Singapore, 117542 Singapore; 6 Department of Medicine, National University Health System, 119228 Singapore, Singapore; 7 Wellcome Trust Sanger Institute, Wellcome Trust Genome Campus, Hinxton, Cambridge, CB10 1SA UK

## Abstract

The Simpson Golabi Behmel Syndrome (SGBS) pre-adipocyte cell strain is widely considered to be a representative *in vitro* model of human white pre-adipocytes. A recent study suggested that SGBS adipocytes exhibit an unexpected transient brown phenotype. Here, we comprehensively examined key differences between SGBS adipocytes and primary human white subcutaneous (PHWSC) adipocytes. RNA-Seq analysis revealed that extracellular matrix (ECM)-receptor interaction and metabolic pathways were the top two KEGG pathways significantly enriched in SGBS adipocytes, which included positively enriched mitochondrial respiration and oxidation pathways. Compared to PHWSC adipocytes, SGBS adipocytes showed not only greater induction of adipogenic gene expression during differentiation but also increased levels of UCP1 mRNA and protein expression. Functionally, SGBS adipocytes displayed higher ISO-induced basal leak respiration and overall oxygen consumption rate, along with increased triglyceride accumulation and insulin-stimulated glucose uptake. In conclusion, we confirmed that SGBS adipocytes, which are considered of white adipose tissue origin can shift towards a brown/beige adipocyte phenotype. These differences indicate SGBS cells may help to identify mechanisms leading to browning, and inform our understanding for the use of SGBS vis-à-vis primary human subcutaneous adipocytes as a human white adipocyte model, guiding the selection of appropriate cell models in future metabolic research.

## Introduction

A human preadipocyte cell strain derived from the stromal vascular fraction of subcutaneous adipose tissue of a male infant with Simpson-Golabi-Behmel syndrome (SGBS) was first described in 2001^1^. SGBS is a rare overgrowth syndrome characterized by a broad spectrum of clinical manifestations including multiple congenital abnormalities, facial and cardiac abnormalities, defective lung segmentation, Wilms tumor, macrocephaly, and organomegaly^2–5^. While it could be lethal in affected males, with up to 50% neonatal mortality rate, the syndrome shows a milder form in female carriers^6–8^. Several affected individuals’ body weights have been reported to be in the >97^th^ percentile^6, 7, 9^ but it is not known whether body temperature is elevated in patients with SGBS syndrome. Wabitsch *et al*. previously reported that SGBS cells responded to catecholamine exposure with an increase in glucose uptake upon insulin stimulation and effective inhibition of cathecolamine-stimulated lipolysis^1^. Genomic rearrangements and point mutations involving the glypican (GPC)-3, GPC-4 and oral-facial-digital syndrome (OFD1) genes have been shown to be associated with SGBS. These genes are involved in the regulation of cell division and growth^5, 10, 11^.

Neither transformed nor immortalized, the SGBS preadipocyte cell strain is capable of retaining their potential for adipose differentiation up to 50 generations^1^. This cell strain thus serves as an almost unlimited source of human preadipocytes and a widely used tool to study human adipocyte biology^12–17^.

During the differentiation process, SGBS cells developed a gene expression pattern similar to that found in differentiating primary human preadipocytes with a characteristic increase in fat cell-specific mRNAs encoding lipoprotein lipase (LPL), glycerol-3-phosphate dehydrogenase (GPDH), GLUT4, leptin and others^1^. Subsequent studies performed in SGBS adipocytes and primary human subcutaneous adipocytes isolated from lipoaspirates showed a similar trend in the induction of FABP4 and PPARγ mRNA and FABP4 secretion during differentiation^18^. Another study characterizing SGBS adipocytes and primary human omental adipocytes derived from obese and non-obese individuals concurred that the cell lines are similar in terms of the morphology, induction of adipocyte-specific gene expression and GPDH activity^13^. However, inherent differences in metabolic profiles of SGBS adipocyte and primary human adipocytes derived from adult subcutaneous depot remain largely unclear. More recently SGBS adipocytes were reported to display a transient feature of brown adipocytes at day 14 and eventual differentiation and maturation to white adipocyte phenotype by day 28 of differentiation^19^. UCP1 expression appeared to be an intrinsic property of SGBS adipocytes as it remained high at day 14 when differentiated in a rosiglizatone free medium. Moreover, when exposed to cold at 30 °C, both UCP1 expression and mitotracker co-staining were elevated, implying a cold stimulus driven inducible brown phenotype^19^. Given that SGBS is often used as a representative model of primary human white SC adipocytes^13, 18, 20–23^, the validation is required to identify and characterize any potential differences in their metabolic phenotype. We conducted a comparative study and found that while both these cell types have overlapping similarities, they also exhibit distinct metabolic signatures that may explain differences in their capacities for adipocyte differentiation and maturation, lipid metabolism and thermogenic activity. Understanding these differences could open opportunities to understand the browning of adipose tissue and also further our knowledge of the strength and limitations of each cell line and provide guidance on the choice of cellular models in future research on human fat cell development and metabolism.

## Results

### Fully differentiated SGBS adipocytes display a transcriptomic profile different to primary human white SC adipocytes (PHWSC)

**Figure 1:**
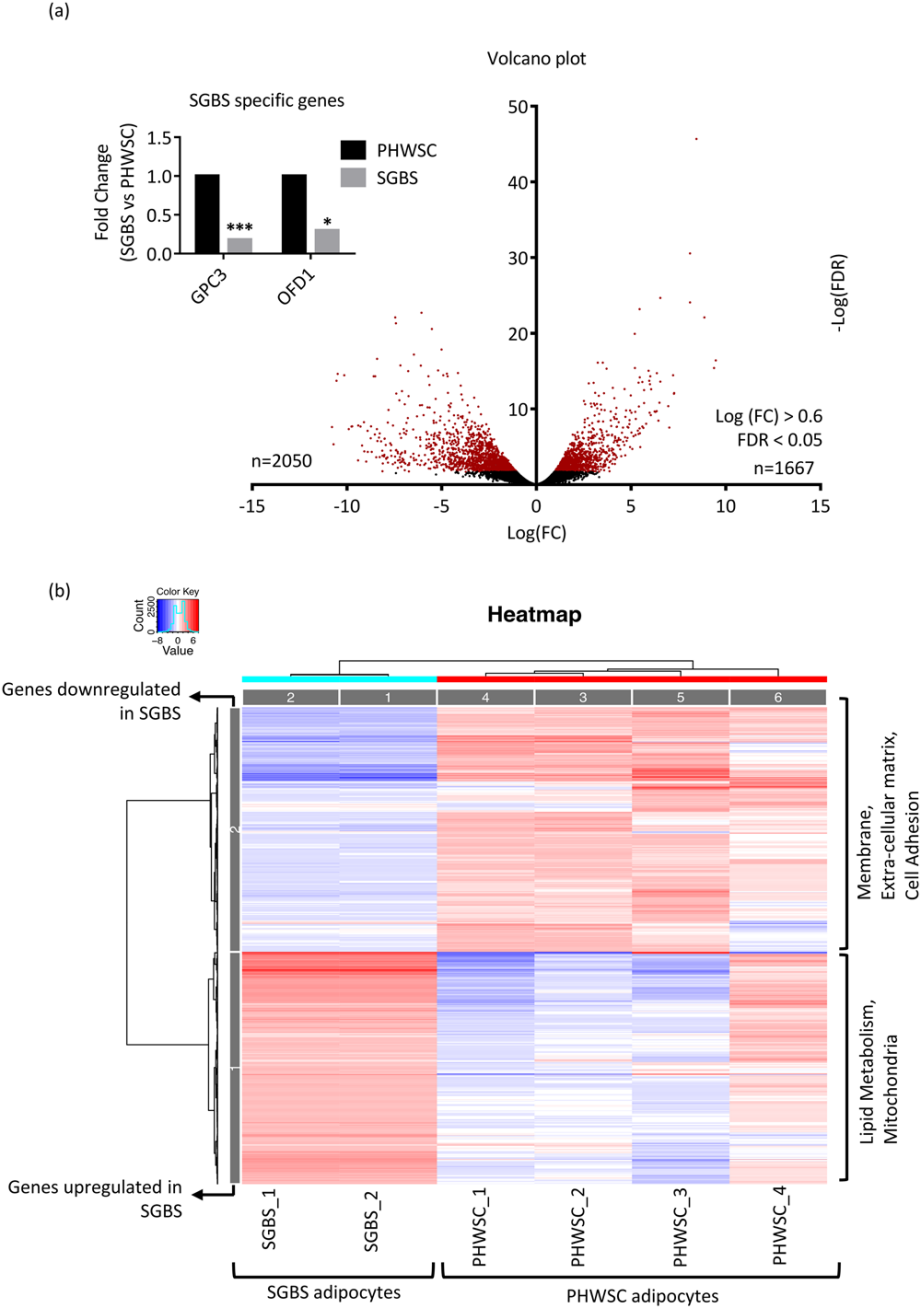
Global transcriptome profiling of SGBS and PHWSC adipocytes (**a**) Volcano plots displaying a number of differentially expressed genes in SGBS adipocytes compared to PHWSC adipocytes. Red dots denote statistically significant genes. Genes with FDR < 0.05 and log (FC) > 0.60 were included. (**b**) Heat map clustering top 1000 significantly upregulated (red) or downregulated (blue) genes between SGBS and PHWSC adipocytes. For PHWSC adipocytes in RNA-Seq, experiments were performed in cells derived from 4 different donors (SC_1 to SC_4, n = 4). For SGBS adipocytes, experiments were performed in duplicates (SGBS_1 and SGBS_2) from the same SGBS adipocyte.

Approximately 15000 genes were read by RNA-Seq, of which 1667 genes were upregulated and 2050 genes were downregulated in SGBS adipocytes relative to PHWSC (Fig. 1a and supplementary Table S2 and supplementary Table S3; log FC > ± 0.6; FDR < 0.05).

Next we performed unsupervised clustering on the top 1,000 genes with the most variable RNA-seq counts across all experiments. The results were visualized using a heat map and clusters were analyzed for enriched gene ontologies (GO). Genes downregulated in SGBS were associated with the membrane (GO_cellular component), extra-cellular matrix (GO_cellular component) and cell adhesion (GO_biological function) gene ontologies. Conversely, genes upregulated in SBGS adipocytes were associated with lipid metabolism (GO_biological function) and mitochondria (GO_cellular component) gene ontologies (Fig. 1b). When we generated an alternate heat map using all differentially expressed genes with a FDR < 0.05 (Supplementary Fig. S1), it was reassuring to see that the enriched gene ontologies were in perfect concordance. We next analysed all differentially expressed genes, both up and downregulated combined, in the context of KEGG pathways. Here, 20 KEGG pathways were enriched in SGBS when compared to PHWSC adipocytes and the top 2 significantly enhanced pathways were ECM-receptor interaction (FDR = 3.18E-06) and metabolic pathways (FDR = 3.07E-05) (Table 1). Genes significantly up or downregulated within the metabolic pathways in SGBS adipocytes were further analysed for KEGG pathway enrichment and an interaction map was built using Cytoscape (Supplementary Table S4 and Supplementary Fig. S2). The interaction map highlighted significant positive enrichment (red coloured nodes) in oxidative phosphorylation, TCA cycle, fatty acid elongation, butanoate metabolism and steroid biosynthesis pathways in SGBS adipocytes when compared to PHWSC adipocytes (Supplementary Fig. S2 and Supplementary Table S4).

**Table 1:** List of KEGG pathways enriched in SGBS adipocytes when compared to PHWSC adipocytes.

KEGG biological pathways	FDR
ECM-receptor interaction	3.18E-06
Metabolic pathways	3.07E-05
Type I diabetes mellitus	0.000142
Fatty acid metabolism	0.000178
Cytokine-cytokine receptor interaction	0.000256
Calcium signaling pathway	0.000443
Autoimmune thyroid disease	0.001638
PI3K-Akt signaling pathway	0.002073
Neuroactive ligand-receptor interaction	0.003842
Pathways in cancer	0.004007
Hematopoietic cell lineage	0.004051
Graft-versus-host disease	0.005511
Cell adhesion molecules (CAMs)	0.005968
Fatty acid degradation	0.006437
Biosynthesis of unsaturated fatty acids	0.009968
Valine, leucine and isoleucine degradation	0.013166
Focal adhesion	0.016408
Allograft rejection	0.018391
PPAR signaling pathway	0.029223
Glycerolipid metabolism	0.044868

Genes with log (FC) > 0.60 and FDR < 0.05 were used as an input in ipathway guide to generate significantly enriched KEGG pathways (P < 0.05, Bonferonni corrected). Pathways are listed in increasing order of P value.

Next, we examined whether RNA-Seq analyses could reveal further insights into the lineage of SGBS adipocytes, referencing a comprehensive list of adipocyte lineage markers reported to date as a base (Table 2). SGBS adipocytes display expression profiles consistent generally with WAT lineage, although upregulation of several markers relating to brown/beige lineage was also seen. Of interest, the UCP1 gene, a bona fide marker of brown fat, along with genes PDK4, PREX1, SIRT1, PLIN5, EAR2, SHOX2, SLC27A1, were markedly upregulated (FDR = 0.037) in SGBS adipocytes, when compared to PHWSC adipocytes.

**Table 2:** List of adipocyte and adipose tissue lineage markers analysed in SGBS adipocytes compared to PHWSC adipocytes.

Gene Name	**Lineage**	**Fold Change (SGBS vs PHWSC)**	**FDR**	**Function of gene/encoded protein**	**Reference**
EDNRB	BAT	−5.14	0.005	UCP1 regulator.	Xue and Tseng *et al*. 2015
EBF2	BAT	−4.68	0.000	Recruits PPARϒ to brown fat-selective gene targets, including PRDM16.	Rajakumari, Wu *et al*. 2013
PDK4	BAT	4.57	0.030	Stimulated by PPARα and PPARδ. Inhibits PDH/TCA cycle, decreasing glucose utilization and promoting fat metabolism.	Mottillo *et al*. 2012
PREX1	BAT	5.74	0.000	UCP1 regulator.	Xue and Tseng *et al*. 2015
MYF5	BAT	NA	NA	Myogenic regulatory factor expressed in skeletal muscle and brown adipocyte precursors.	Seale, Bjork *et al*. 2008
ZIC1	BAT	NA	NA	Expressed in anterior somites in embryonic development and in anterior adipose tissues.	De Jong and Larsson *et al*. 2015
BMP7	BAT	NA	NA	Promotes browning; induces PRDM16, PGC1α, UCP-1, PPARϒ, C/EBPs.	Tseng *et al*., 2008
MTUS1	BAT and beige	−61.91	0.000	A mitochondria-localized protein. Required for beige adipocyte differentiation and thermogenic function.	Shinoda, Luijten *et al*. 2015
PGC1α	BAT and beige	−20.66	0.000	Promotes browning. Stimulates expression of FNDC5/Irisin.	Bostrom, Wu *et al*. 2012
HSPB7	BAT and beige	−5.22	0.002	Marker gene. Function in adipose tissue is unclear.	Wu, Bostrom *et al*. 2012
EBF3	BAT and beige	−2.18	0.015	Transcription factor.	De Jong and Larsson *et al*. 2015
PRDM16	BAT and beige	−1.74	0.449	Encodes a Zinc-finger protein. Induces browning via PGC1α, UCP-1 and other key regulatory genes.	Seale *et al*. 2007
KCNK3	BAT and beige	−1.40	0.819	A potassium channel. Required for beige adipocyte differentiation and thermogenic function.	Shinoda, Luijten *et al*. 2015
SIRT1	BAT and beige	1.90	0.024	Deacetylates PPARϒ to facilitate docking of PRDM16. Promotes browning.	Qiang, Wang *et al*. 2012
UCP1	BAT and beige	8.84	0.037	Uncouples respiration to dissipate chemical energy as heat.	Wu, Bostrom *et al*. 2012
PLIN5	BAT and beige	9.50	0.010	Expressed in highly oxidative tissues like heart and BAT. Possible regulation of ATGL-mediated lipolysis. Suggested protective role against excessive production of reactive oxygen species by controlling FA mitochondrial oxidation and their incorporation to LDs.	Barneda, Frontini *et al*. 2013
LHX8	BAT and beige	NA	NA	Transcription factor.	De Jong and Larsson *et al*. 2015
FGF21	BAT and beige	NA	NA	Stimulates glucose uptake in differentiated adipocytes via induction of GLUT1 expression.	De Jong and Larsson *et al*. 2015
CIDEA	BAT and beige	NA	NA	Promotes LD enlargement by LD–LD lipid transference. Cide (A, B or C) deficient mice are resistant to obesity.	Barneda, Frontini *et al*. 2013
PAT2	BAT and beige	NA	NA	An amino acid transmembrane transporter.	Ussar, Lee *et al*. 2014
P2RX5	BAT and beige	NA	NA	A purinergic receptor and ligand-gated ion channel.	Ussar, Lee *et al*. 2014
B3AR/ADRB3R	BAT and beige	NA	NA	Receptor which mediates catecholamine-induced browning.	Lidell *et al*. 2013
CD40	Beige	−6.62	0.002	Important in immune and inflammatory response pathways. Member of TNF-receptor superfamily.	Wu, Bostrom *et al*. 2012
EPSTI1	Beige	−4.18	0.011	Marker gene. Function in adipose tissue is unclear.	De Jong and Larsson *et al*. 2015
SP100	Beige	−2.44	0.007	Binds heterochromatin proteins; involved in gene regulation.	Wu, Bostrom *et al*. 2012
EAR2/NR2F6	Beige	1.85	0.000	Transcription factor.	Wu, Bostrom *et al*. 2012
SHOX2	Beige	2.03	0.063	Member of homeobox family.	Lidell 2013
SLC27A1	Beige	4.48	0.000	Component of lipid metabolism pathways.	Wu, Bostrom *et al*. 2012
TBX1	Beige	NA	NA	A developmental transcription factor	De Jong and Larsson *et al*. 2015
TNFRSF9/CD137	Beige	NA	NA	Member of TNF-receptor superfamily.	De Jong and Larsson *et al*. 2015
TMEM26	Beige	NA	NA	Marker gene. Function in adipose tissue is unclear.	De Jong and Larsson *et al*. 2015
PDGFRα	WAT and Beige	−8.03	0.000	A growth factor receptor expressed by bipotent preadipocytes.	Yun-Hee Lee 2012
HOXC9	WAT and Beige	−1.68	0.205	Developmental transcription factor with posteriorly restricted expression.	De Jong and Larsson *et al*. 2015
HOXC8	WAT and Beige	−1.27	0.599	Developmental transcription factor with posteriorly restricted expression. A negative regulator of the adipocyte browning process.	De Jong and Larsson *et al*. 2015
RBL1	WAT	−2.29	0.050	Represses PGC1α transcription, preventing browning.	Scime, Grenier *et al*. 2005
Leptin	WAT	−0.71	0.530	A secreted protein involved with regulation of body weight.	Ussar, Lee *et al*. 2014
NRIP1/RIP140	WAT	1.09	0.827	Binds and inhibits PGC1α.	Hallberg, Morganstein *et al*. 2008
RB1	WAT	1.34	0.380	Represses PGC1α transcription, preventing browning.	Hansen, Jorgensen *et al*. 2004
TCF21	WAT	2.94	0.026	Developmental transcription factor with posteriorly restricted expression.	De Jong and Larsson *et al*. 2015
LPL	WAT	13.47	0.001	Involved in the metabolism of fat.	Dani, Amri *et al*. 1990
NR1H3/LXRA	WAT	16.32	0.000	Blocks UCP1 expression by recruiting Rip140 and displacing PGC1α at an LXR binding site.	Wang, Zhang *et al*. 2008
ASC-1/slc7a10	WAT	17.47	0.000	A cell surface amino acid transporter.	Ussar, Lee *et al*. 2014
ADIPONECTIN	Ubiquitous in AT	12.44	0.003	Hormone that regulates lipid and glucose metabolism and modulates insulin sensitivity.	Zhang, Matheny *et al*. 2002
CITED1	Ubiquitous in AT	48.18	0.000	Involved with CBP/SMAD signaling.	De Jong and Larsson *et al*. 2015

Genes were pooled from published literature and their expression was analysed in the RNA sequencing data.

### SGBS and PHWSC adipocytes exhibit distinct gene expression patterns during differentiation

**Figure 2:**
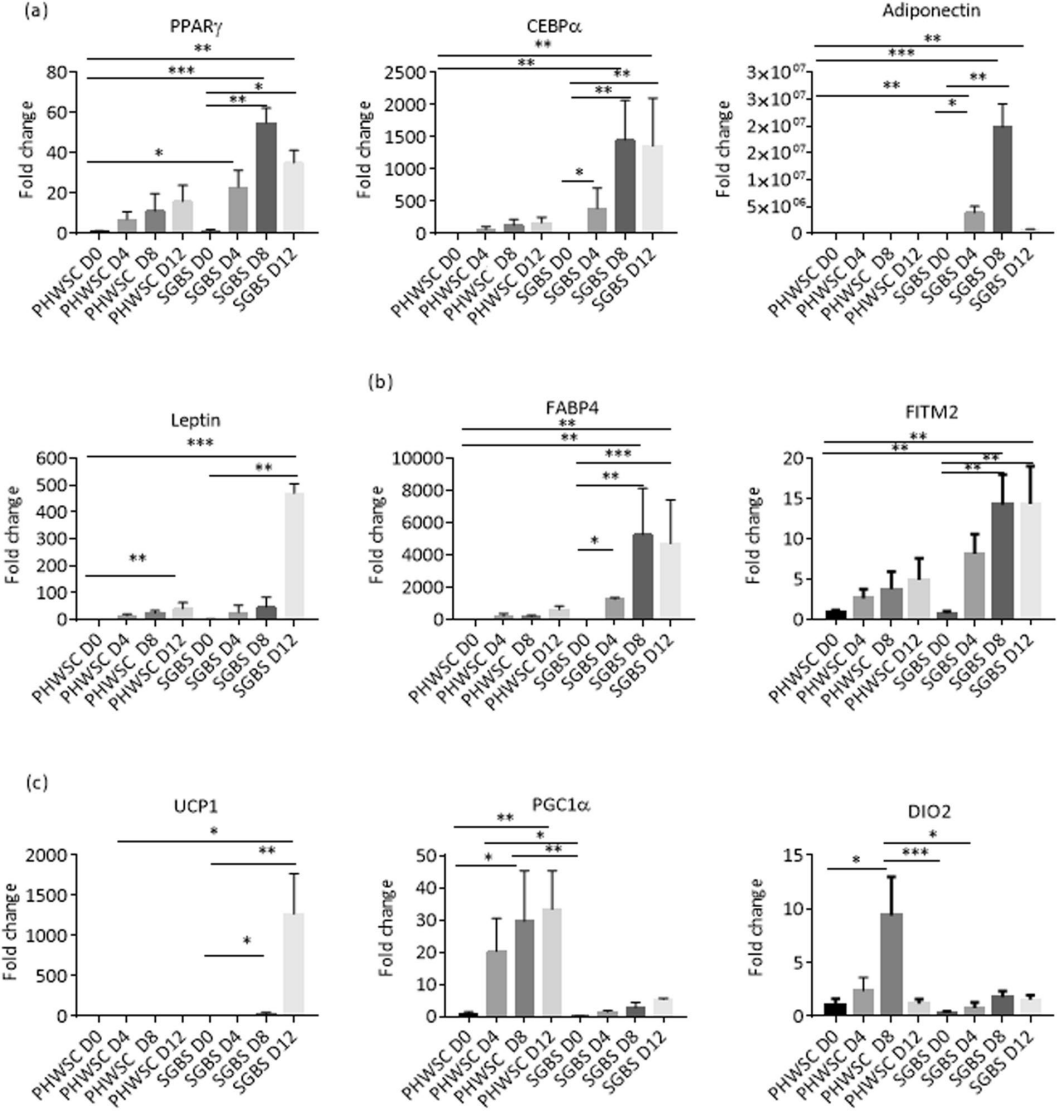
Expression profiling of differentiating SGBS and PHWSC adipocytes, for genes involved in (**a**) adipogenesis (**b**) lipid storage (**c**) browning. Days of differentiation are indicated as D0, D4, D8, D12 respectively. For experiments involving PHWSC adipocytes, experiments were performed in duplicates from cells derived from 3 different donors. For SGBS adipocytes, experiments were performed in duplicates for 3 independent experiments from the same SGBS adipocyte. Data represented as mean ± SD and was normalized to SC control D0 samples. Two-way ANOVA was used to test for statistical significance. *P < 0.05, **P < 0.01, ***P < 0.001, ****P < 0.0001.

We next analysed when these cellular models might diverge. Adipogenesis was initiated in SGBS and PHWSC adipocytes and RNA from both cell types was harvested on days 0, 4, 8, and 12 for gene expression studies. Nine genes-of-interest relating to adipogenesis, lipid storage and metabolism, and browning were chosen for real-time PCR validation. With increasing days of differentiation, both SGBS and PHWSC preadipocytes showed upregulation of genes involved in adipocyte differentiation but the magnitude of induction of PPARγ, CEBPα, adiponectin and leptin was 10–15 folds higher in SGBS compared to PHWSC adipocytes (Fig. 2a) suggesting they had facilitated differentiation. For instance, using PHWSC adipocytes at day 0 as a reference, the fold change for adiponectin was about 14 times lower in PHWSC adipocytes when compared to SGBS at D12. Lipid storage genes FABP4 and FITM2 also showed a significant upregulation over the course of adipocyte differentiation (Fig. 2b). FITM2 (fat storage-inducing transmembrane proteins 2) is involved in triglyceride partitioning and its gene expression levels has been previously reported to increase with differentiation in 3T3-L1 mouse adipocytes^24, 25^. PGC1α, a transcriptional co-activator of UCP1 expression and a key gene in cellular energy metabolism^26, 27^, was significantly higher in PHWSC adipocytes when compared to SGBS (P < 0.05). Interestingly, the expression of browning marker UCP1 was almost non-existent in PHWSC adipocytes with approximately 1200 fold higher expression in SGBS adipocytes (Fig. 2c). The discordance between UCP1 and PGC1α expression may be due to alternative transcriptional activators of UCP1 mRNA in SGBS adipocytes^27^. Gene expression data assessed using RNA-Seq and qRT-PCR methods showed good correlation (r = 0.8289, P < 0.0002, supplementary Fig. S3).

### SGBS adipocytes display some features consistent with a UCP1-driven thermogenic machinery

**Figure 3:**
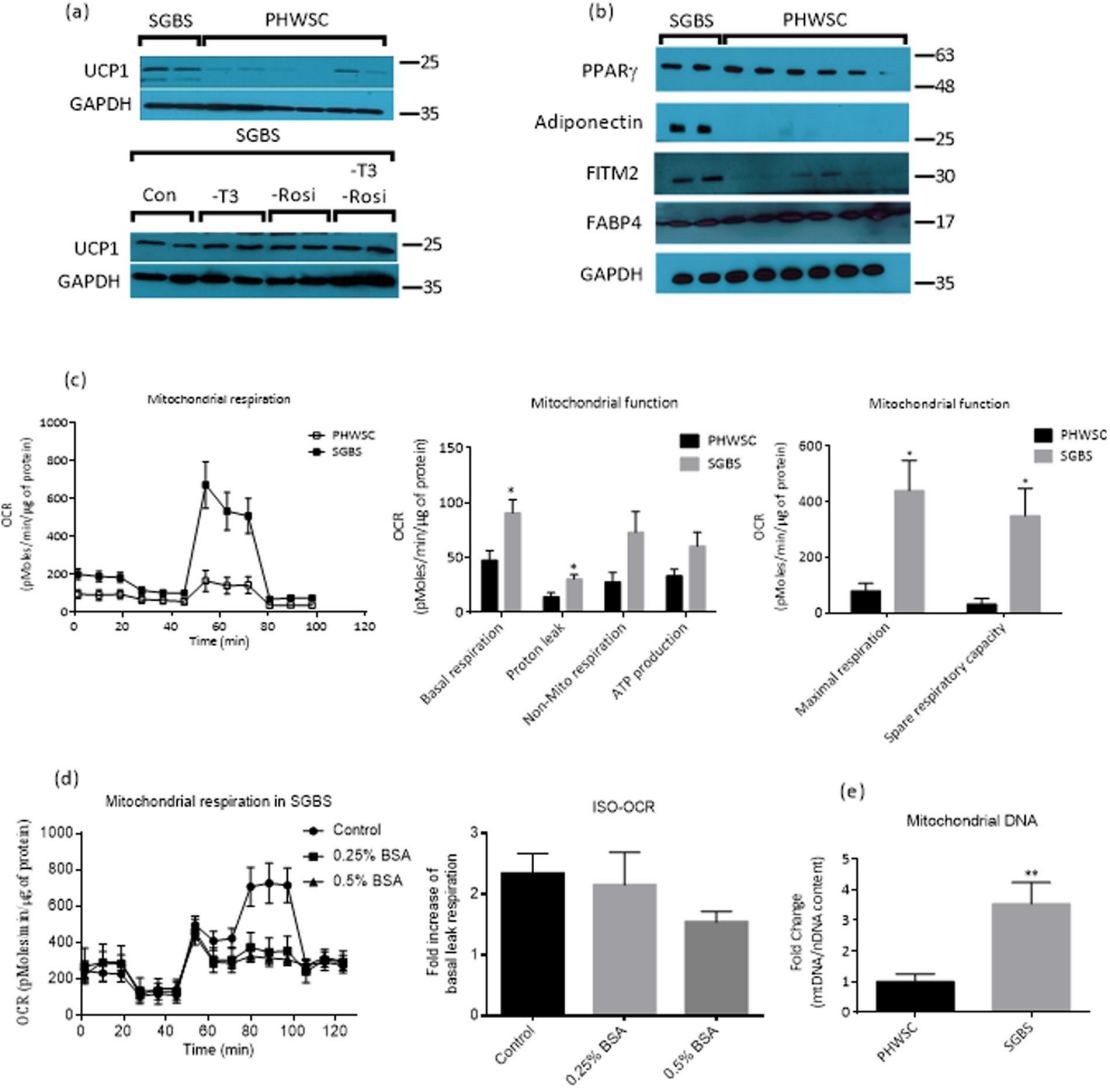
UCP1 protein expression and mitochondrial respiration in SGBS adipocytes display characteristics suggestive of browning capacity. (**a**) Top panel: UCP1 protein expression in differentiated SGBS and PHWSC adipocytes. Bottom panel: UCP1 protein expression in SGBS adipocytes differentiated in medium depleted with either T3 or rosiglitazone or both. (**b**) Protein expressions of adipogenic and lipid metabolism markers including PPARγ, adiponectin, FITM2 and FABP4 in differentiated SGBS and PHWSC adipocytes. (**c**) Mitochondrial respiratory function or oxygen consumption rate (OCR) measured by Seahorse mitochondrial flux analyser. Bar graphs denote functional readouts of mitochondrial oxidative capacity. (**d**) Representative time course of OCR of ISO-treated SGBS adipocytes in the presence of either 0%, 0.25% or 0.5% FAF BSA, as well as UCP1-mediated basal leak respiration in SGBS adipocytes. (**e**) Relative mitochondrial DNA content in SGBS and PHWSC adipocytes. UCP1 protein blots were cropped for a more concise presentation and the respective full-length blots are available in supplementary Fig. S4. For the experiments involving PHWSC adipocytes, experiments were performed in duplicates from cells derived from 3 different donors. For SGBS adipocytes, experiments were performed in duplicates during 3 independent experiments from the same SGBS adipocyte. Data are expressed as means ± SD. *P < 0.05.

Consistent with the gene expression data, Fig. 3a and supplementary Fig. S4 show that UCP1 protein expression was significantly higher in mature SGBS adipocytes as compared to PHWSC adipocytes. UCP1 protein expression persisted in SGBS adipocytes even after depletion of T3, rosiglitazone or both from the differentiation media (Fig. 3a). Several key adipogenic markers were also examined for their protein expression levels. While PPARγ and FABP4 appeared to be similar between the two cell types, the protein expression levels of adiponectin and FITM2 were found to be significantly higher in SGBS adipocytes (Fig. 3b). In order to assess whether upregulation of UCP1 gene and protein expression had functional significance in SGBS adipocytes, we measured mitochondrial DNA content and mitochondrial function in both adipocyte cell types. As shown in Fig. 3c, metabolic signatures of SGBS were clearly different from PHWSC adipocytes. Basal respiration, maximal respiration, and spare respiratory capacity were significantly higher in SGBS when compared to PHWSC adipocytes (P < 0.05). At a glance (Fig. 3c), the leak respiration, which represents uncoupling of oxidative phosphorylation, was significantly higher in SGBS adipocytes (P < 0.05). To test if the observation was UCP1-dependent, we measured OCR and leak respiration in adipocytes treated with isoproterenol (ISO) in the presence of increasing dose of fatty acid free (FAF)-BSA to sequester free fatty acids released during lipolysis (Fig. 3d). ISO treatment led to a sharp rise in OCR and as expected, introduction of FAF-BSA diminished the effect of ISO in a dose dependent manner. ISO-induced leak respiration, which is considered to be a better representation of UCP1 activity was nearly two fold higher than basal leak respiration in absence of FAF-BSA. The presence of 0.5% FAF-BSA significantly reduced ISO-induced leak respiration when compared to no-BSA or 0.25% FAF-BSA conditions. There was significantly higher ratio of mitochondrial DNA to nuclear DNA content (P = 0.0079) in SGBS adipocytes (FC = 3.54 ± 0.73) when compared to PHWSC adipocytes (Fig. 3e). When the mitochondrial respiration data was normalized to mitochondrial DNA content, the differences between SGBS and PHWSC adipocytes were no longer significant. Hence, the differences in mitochondrial respiration could be contributed by a combination of factors, including differences in mitochondrial DNA content, mitochondrial activity and the thermogenic activity in SGBS adipocytes, which are partially but not fully mediated by UCP1.

### Higher lipid accumulation and greater insulin stimulated glucose uptake in SGBS adipocytes

**Figure 4:**
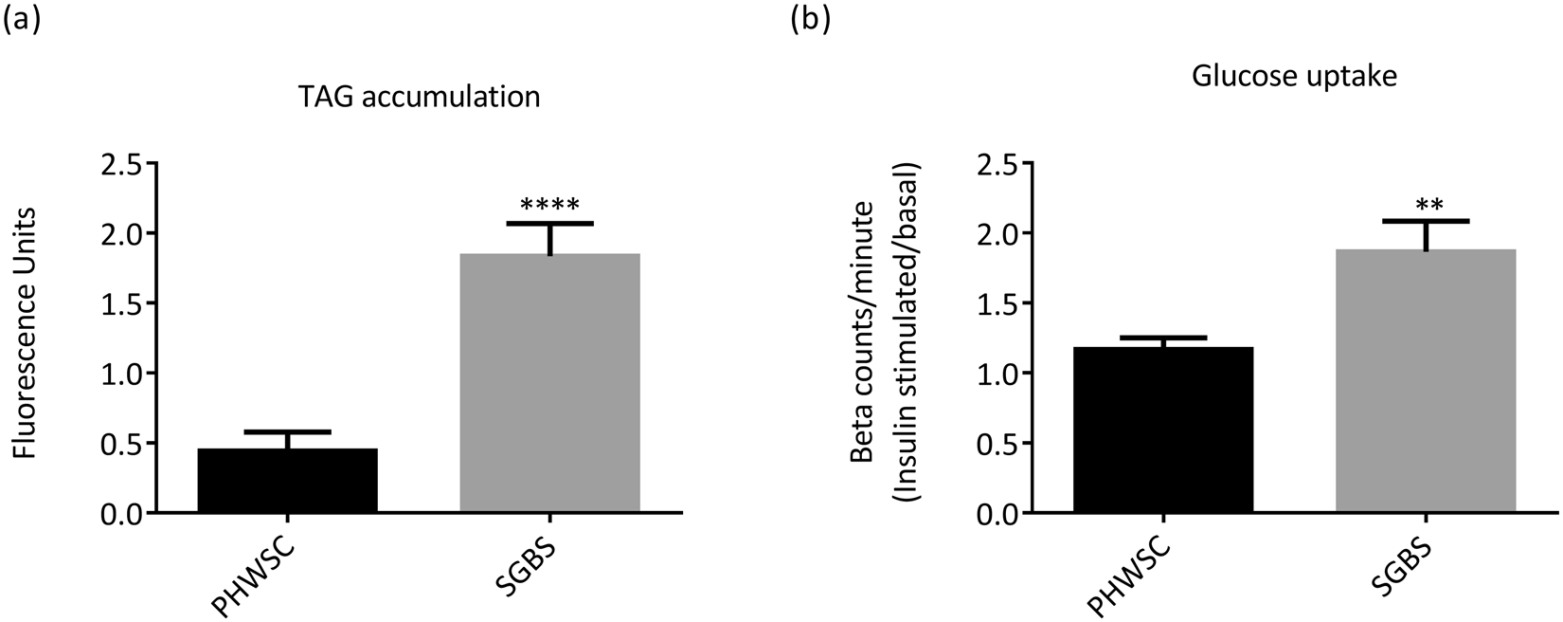
SGBS adipocytes have higher lipid accumulation and insulin stimulated glucose uptake. (**a**) Total lipid accumulation in SGBS and PHWSC adipocytes. Lipid accumulation was quantified as florescence after incubation of adipocytes with Nile red dye. (**b**) Relative insulin-stimulated glucose uptake assay in SGBS versus PHWSC adipocytes. Glucose uptake was measured using radiolabelled de-oxy glucose. For the experiments involving PHWSC adipocytes, experiments were performed in duplicates from cells derived from 3 different donors. For SGBS adipocytes, experiments were performed in duplicates during 3 independent experiments from the same SGBS adipocyte. Data represented as Means ± SD. **P < 0.01, ****P < 0.0001.

It is known that BAT activation plays a role in regulating glucose and lipid metabolism^28^. Intracellular lipid droplet accumulation was assessed using the hydrophilic stain Nile red (AdipoRed), which fluoresces in the presence of hydrophobic triglycerides. SGBS adipocytes were observed to have accumulated 4 times more triglycerides than PHWSC adipocytes at D12 post differentiation (P < 0.0001) (Fig. 4a). Finally, we performed insulin-stimulated radioactive glucose uptake assay to assess any potential differences in insulin action between the two cell types. SGBS adipocytes showed significantly higher (P < 0.0079) relative glucose uptake (1.87 ± 2.2) in response to insulin stimulation compared to PHWSC adipocytes (1.17 ± 0.08) (Fig. 4b). It is generally observed that mouse adipocytes, such as 3T3-L1 adipocytes, are more responsive to insulin stimulation, which would make it a more suitable model for studying glucose uptake^29–31^, when compared to both SGBS and PHWSC adipocytes.

## Discussion

The present study demonstrated that mature SGBS adipocytes and primary white human subcutaneous adipocytes derived from obese, non-diabetic individuals display characteristics that are metabolically distinct. Of note, the SGBS is an overgrowth disorder and although not all causes of SGBS have been identified, one cause of SGBS is a mutation of the glypican-3 gene (GPC3) on the X chromosome locus q26.1. Moreover, GPC3 is known to regulate Hedgehog signalling during development of tissues, including mesenchyme and progenitors of adipose tissue. However, a GPC3 mutation was not found in the seminal paper that described the characteristics of the SGBS cell line^1^. The precise mutation in the SGBS adipocytes widely used as a human derived *in vitro* model for adipocyte development and metabolism has not yet been characterised. However, transcriptomic analysis and the clustering of differentially expressed genes revealed several biological pathways enriched in SGBS adipocytes compared to the PHWSC adipocytes. The two most significant paths relate to extracellular matrix (ECM) and metabolic pathways.

ECM plays a critical role in maintaining the structural integrity and communication between adipocytes. ECM-receptor interactions are associated with pro-inflammatory changes and dysregulation of adipocyte metabolism; excessive accumulation of ECM is known to correlate positively with BMI levels^32, 33^ and obesity-related inflammation contributing to a adipose tissue dysfunction and reduced adipose tissue expandability^21^. Our results showed 32 genes involved in ECM-receptor interactions being significantly up-regulated in PHWSC adipocytes when compared to SGBS adipocytes. One confounding factor, however, could be the obese status of the subjects in this study. This could be potentially addressed by other studies that involve adipocytes derived from lean subjects in the future.

The expression of genes involved in metabolic pathways and mitochondrial respiration differed greatly between SGBS and primary adipocytes derived from obese adult individuals. Differences in protein expression levels of adipogenic markers such as adiponectin and FITM2 provided further evidence that the overall adipocyte regulation were clearly distinct between the two cell types. Interestingly, previous studies have shown that adiponectin could modulate UCP1 expression in rodent BAT and reducing visceral fat mass, preventing adipocyte hypertrophy, accelerating overall energy expenditure^34^ and also enhance cold-induced browning of SC adipose tissue by promoting M2 macrophage polarization^35^, suggesting that adiponectin may have a role in regulating UCP1 expression in SGBS adipocytes. 220 genes implicated in metabolism were significantly upregulated and 94 genes were significantly downregulated in SGBS as compared to PHWSC. The two metabolic genes that differed the most in their expression were ALDH3B2 and CYP19A1. ALDH3B2 is involved in the removal of aldehydes generated under conditions of oxidative stress within lipid droplets^36^, and it was found to be ~350 fold higher in SGBS adipocytes. CYP19A1, also known as aromatase, is responsible for the aromatization of A ring of C19 steroids. Typically, it is expressed at very low levels and hardly detectable in fully differentiated, lipid-laden adipocytes^37, 38^. In agreement with this, our data showed its expression to be more than 300 fold lower in SGBS adipocytes when compared to PHWSC adipocytes. Even though McInnes *et al*. reported that the aromatase expression in SGBS mimics that of isolated primary human adipocytes^14^, the changes in expression levels were not compared in parallel with primary human adipocytes and the origin of primary human adipocytes used was unclear. Further studies will be needed to verify the differences in observations and whether there is any functional significance in the regulation of genes mentioned above.

Our results showed that probably the most compelling difference between SGBS adipocytes and PHWSC lies in UCP1 mRNA and protein expression as well as its higher mitochondrial oxygen consumption profile. It is tempting to speculate that both UCP1-dependent and UCP1-independent leak respiration were involved in the energy metabolism of SGBS adipocytes. UCP1-independent leak respiration could be due to the inherently high mitochondrial content^39^, elevated oxygen consumption rate, alternatively increase fatty acid flux^40^, and even UCP1 being inserted incorrectly into the membrane, making it constantly leaky^41^. An alternative UCP1-independent futile thermogenic pathway, analogous to the futile creatine cycle described by Kazak *et al*.^42^, might also be involved in maintaining metabolic homeostasis in SGBS adipocytes. Future work to address these missing links would be warranted. The gene expression patterns in Table 2 confirmed that the more consistent patterns of SGBS involved white adipose tissue lineage markers, supported by upregulation of genes typically expressed in WAT, yet capable of displaying its thermogenic properties at the same time.

The fact that SGBS cells are derived from the white adipose tissue of a 3-month-old infant could also be a key contributing factor to the differences observed^1^. It is known that the function and composition of adipose tissue vary substantially between an early age and in adulthood. In infancy, it performs an active role in heat generation and principally comprises brown adipose tissue (BAT). By adulthood, it performs a more passive role in preventing heat loss and the composition shifts in favour of WAT. But even WAT from neonates is significantly different to that from adults. It is capable of responding to β-adrenergic stimulus and showed enhanced oxygen consumption rate when compared to adult adipocytes^43^. In addition, adipocytes derived from neonatal interscapular region expressed detectable levels of UCP1 mRNAs and showed molecular signatures of developmentally-programmed BAT (e.g. high expression of ZIC1)^44^. It would be interesting for future studies to fully characterise WAT from a healthy infant, and compare these characteristics to the SGBS cells.

Given their high differentiation capacity up to 50 passages^1, 20^, SGBS cells remain as a valuable *in-vitro* test model for studies related to human adipocyte metabolism. However, given the differences observed, we would caution against solely using SGBS to model primary human adult subcutaneous adipocytes. Understanding similarities and differences between the different adipocyte models can assist in the selection of appropriate models in future studies that examine human fat cell development and metabolism.

## Methods

### Cell culture

All subjects recruited for this study were patients from the National University Health System (NUHS, Singapore) who underwent bariatric surgery and had a body mass index (BMI) of ≥ 35 kg/m^2^. All participants provided informed consent and ethics approval was obtained from the National Healthcare Group Domain Specific Review Board (reference number 2014/00396, Singapore). All procedures were carried out in accordance with the approved guidelines and regulations. Fresh adipose tissue specimens were obtained laparoscopically from the subcutaneous (SC) abdominal region during the surgery, without any additional surgical procedures. Primary human SC preadipocytes derived from 4 different individuals were prepared by collagenase digestion using a previously established protocol^45^. Once the cells displayed fibroblastic morphology, adipogenesis was initiated using commercial preadipocyte growth media and differentiation cocktail (Lonza, MD, USA). SC adipocytes were harvested on Day 12 and are henceforth referred to as the PHWSC adipocytes. Human SGBS adipocytes were kindly donated by Professor David Silver from DUKE-NUS, Singapore. SGBS cell growth and differentiation were performed using the standard protocol^1, 20^. The optimal media recommended for differentiating SGBS adipocytes differ from the media generally used for PHWSC adipocytes. To confirm that the differences observed between the two adipocytes types were not attributable to the composition of the respective growth and differentiation cocktail, Lonza media was used to differentiate SGBS adipocytes on a separate occasion. Characteristics for SGBS adipocytes were found to be consistent using either type of media (data not shown).

### RNA-Seq experiment and analysis

Total RNA was extracted from SGBS adipocytes (in duplicates) and PHWSC adipocytes (n = 4 derived from 3 females and 1 male with an average age of 39.5 ± 15.9 years old) using Qiagen RNeasy plus kit (Qiagen Inc, CA, USA). Poly-A mRNA was then enriched with oligodT beads (Life Technologies) from approximately 5 µg of total RNA. 100 ng of poly-A mRNA recovered was used to construct multiplexed strand-specific RNA-Seq libraries as per manufacturer’s instructions (NEXTflexTM Rapid Directional RNA-Seq Kit (dUTP-Based) v2). Individual library quality was assessed with an Agilent 2100 Bioanalyzer and quantified with a QuBit 2.0 fluorometer before pooling for sequencing on a HiSeq 2000 (1 × 101 bp read). The pooled libraries were quantified using the KAPA quantification kit (KAPA Biosystems) prior to cluster formation. 20–26 million reads were mapped in all samples with a rate of >95%. Fastq-formatted reads were processed with Trimmomatic^46^ to remove adapter sequences and trim low quality bases (LEADING: 3 TRAILING: 3 SLIDINGWINDOW: 4:15 MINLEN: 36). Reads were aligned to the human genome (hg19) using Tophat version 2 (settings–no-coverage-search–library-type = fr-firststrand). Feature read counts were generated using htseq-count (Python package HTSeq^47^ default union-counting mode, strand = reverse). Differential Expression analysis was performed using the edgeR package in both ‘classic’ and generalized linear model (glm) modes to contrast the SGBS cell strain and primary human subcutaneous adipocytes (PHWSC adipocytes) derived from obese subjects. Genes with at least 1 count per million reads were included for edgeR analysis. Unsupervised clustering was performed using the heatmap.3 function from the GMD R package^48^. The raw htseq-counts were first normalized using variance stabilizing transformation from the DESeq2 package^49^ before cluster analysis. iPathwayGuide (Advaita bioinformatics) and DAVID Bioinformatics, web-based analysis tools were further employed to identify the top biological pathways and metabolic pathways respectively. KEGG pathway interaction map of metabolism related genes was built in Cytoscape version 3.3 using CluePedia and ClueGo plugin^50, 51^.

### Real-time PCR and mitochondrial DNA content

Total RNA was isolated using Qiagen RNeasy plus kit and quantified using nano-drop 2000 spectrophotometer (Thermo Scientific, IL, USA). cDNA was synthesized from 800 ng of RNA using ABI high capacity cDNA synthesis kit (Applied Biosystems, CA, USA). Real-time quantitative polymerase chain reaction (qPCR) using Qiagen QuantiFast SYBR Green PCR Kit was employed to quantify relative gene expression in samples. Primers involved in various pathways of interest such as adipogenesis (PPARγ, CEBPα, Adiponectin, Leptin), lipid storage (FABP4, FITM2) and browning (UCP1, PGC1α, DIO2) were prioritized in this study^1, 13, 19, 20, 25, 52–55^. NCBI primer blast and ABI primer express 3.0 were used to generate primer sequences (Supplementary Table S1).

Differences in mitochondrial DNA content between SGBS and PHWSC adipocytes was determined by real-time PCR using TaqMan probes specific for mitochondrial DNA (mtDNA) and nuclear DNA (nDNA or 18 s rRNA) as previously described (Supplementary Table S1)^56^. Taqman probes were labelled at the 5′ end with fluorescent reporter FAM and the 3′ ends were labelled with a quencher TAMRA. mtDNA content was measured by the ratio of relative expressions of mtDNA to that of nDNA.

### Western blotting

The cellular lysate was obtained using RIPA buffer (Thermo Scientific) supplemented with protease and phosphatase inhibitors. Briefly, 30 µg of protein from cell culture lysates was separated using 10% acrylamide gel, transferred to nitrocellulose membrane, blocked with 5% milk (0.1% Tween 20), and incubated overnight at 4 °C separately with UCP1 (Thermo Scientific), PPARγ (Santa Cruz, TX, USA), adiponectin (Abcam, MA, USA), FITM2 (antibody made in-house, kindly provided by Dr. David Silver, DUKE-NUS, Singapore), FABP4 (Santa Cruz, generously given by Dr. Sun Lei, DUKE-NUS, Singapore), and GAPDH (Cell Signalling) antibodies. Following overnight incubation, blots were incubated with HRP-conjugated secondary antibody for 2 h and exposed to x-ray films using chemiluminescent detection method (EMD Millipore, MA, USA).

### Mitochondrial respiration analysis

Seahorse XF^e^24 analyzer (Seahorse Biosciences, MA, USA) was used to measure mitochondrial respiration in adipocytes. SGBS and PHWSC adipocytes were seeded on XF^e^24 tissue culture plates at 25,000 cells/well and were differentiated as described above. On day 12, maintenance media was replaced by pH 7.4 XF assay medium (Seahorse Biosciences), containing 1 mM pyruvate and 25 mM glucose and adipocytes were kept for incubation at 37 °C ambient CO_2_ free incubator for 45 min; The concentration of dissolved oxygen above the cell monolayer was measured by the XF^e^24 analyzer solid state sensor probe and the change in dissolved oxygen concentration is known as the oxygen consumption rate (OCR). Baseline OCR (OCR Baseline) was measured 3 times for 4 min each separated by a 2 min mix and a 2 min wait. Following the measurement of basal respiration, ATPase inhibitor oligomycin (2 µM) (Sigma–Aldrich, Oakville, ON, Canada) was injected to measure proton leak (OCR oligomycin). A mitochondrial inner membrane uncoupler, carbonyl cyanide p-trifluoromethoxyphenylhydrazone (FCCP) (1.2 µM) (Sigma–Aldrich), was subsequently introduced to drive maximal respiration (OCR FCCP). Finally, a mixture of rotenone (1 μM) and antimycin A (1 μM) (OCR R/A) was added to block respiratory chain complexes I and III and thus check for non-mitochondrial respiration. Parameters of mitochondrial bioenergetics were expressed as a percentage of the basal OCR. For further evaluation of UCP1-dependent leak respiration, cells were replaced with assay medium in the presence or absence of 0.25% or 0.5% BSA to sequester free fatty acids and block intracellular fatty acid-mediated proton leakage. 1 µM isoproterenol (ISO) were added post-oligomycin injection to drive β-adrenergic stimulation of lipolysis^39, 57^. Oxygen consumption rates were normalized to protein concentration. ISO-induced leak respiration rates were deduced from means of 4 highest values and represented as fold increase of basal leak respiration.

### Lipid accumulation and radioactive glucose uptake assay

Differentiated adipocytes were washed with PBS and stained with AdipoRed (Lonza) according to the manufacturer’s protocol. After 30 min, fluorescence readings representing triglyceride accumulation were measured with excitation at 485 nm and emission at 572 nm. Insulin-stimulated glucose uptake assay was performed as previously described with some modifications^58^. Differentiated adipocytes were first incubated in serum-free media supplemented with 0.2% BSA for 6 h prior to the assay. Subsequently, they were washed to completely remove glucose and were incubated in PBS with 0.2% BSA for 30 min at 37 °C. Cells were subjected to insulin stimulation (100 nM) for 15 min and the assay was initiated by the addition of [^3^H] 2-deoxyglucose (5 µCi/mL) and deoxyglucose (5 mM). At the end of 20 min incubation, the activity was terminated by washing the cells three times with ice-cold PBS. Cells were then lysed using RIPA buffer and incubated on ice for 20 min. Finally, cell-associated radioactivity was determined using a liquid scintillation counter (Perkin Elmer).

### Statistics

For the RNA-Seq studies, PHWSC adipocytes derived from 4 independent donors were being used (n = 4); while for all other experiments, adipocytes from 3 independent donors were used (n = 3), in duplicates. Statistical differences were analysed accordingly using paired t-test and Kruskal–Wallis one-way ANOVA followed by Dunn’s test for multiple comparisons. Values were expressed as mean ± SD and P < 0.05 was considered statistically significant. Statistical tests used in RNA-Seq analysis were mentioned in respective Fig. legend.

## Electronic supplementary material

Supplementary information
