# Supplementary material for: SGBS cells as a model of human adipocyte browning: A comprehensive comparative study with primary human white subcutaneous adipocytes
Source: Sci Rep. 2017 Jun 22;7:4031. doi: 10.1038/s41598-017-04369-2 (PMC5481408; doi:10.1038/s41598-017-04369-2)
Supplement: Supplementary file 1 — Supplementary information [file 41598_2017_4369_MOESM1_ESM.pdf]

SGBS cells as a model of human adipocyte browning: A comprehensive comparative study with primary human white subcutaneous adipocytes

Chia Rou Yeo[1,*], Madhur Agrawal[1,*], Shawn Hoon[2], Asim Shabbir[3], Manu Kunaal Shrivastava[4], Shiqi Huang[5], Chin Meng Khoo[1,6], Vanna Chhay[1], M. Shabeer Yassin[1], E Shyong Tai[1,6], Antonio Vidal-Puig[4], Sue-Anne Toh[1,5]

[1]Department of Medicine, Yong Loo Lin School of Medicine, National University of Singapore, 117599, Singapore

[2]Molecular Engineering Laboratory, Biomedical Sciences Institutes, A*Star, 138668, Singapore

[3]Department of Surgery, National University Hospital, 119074, Singapore

[4]School of Clinical Medicine, University of Cambridge, Cambridge, CB2 0SP, United Kingdom

[5]Food Science and Technology Program, Department of Chemistry, National University of Singapore, 117542, Singapore

[6]Department of Medicine, National University Health System, 119228, Singapore

*These authors contributed equally to this work.

Correspondence and requests for materials should be addressed to S.A.T (email: mdcsates@nus.edu.sg)

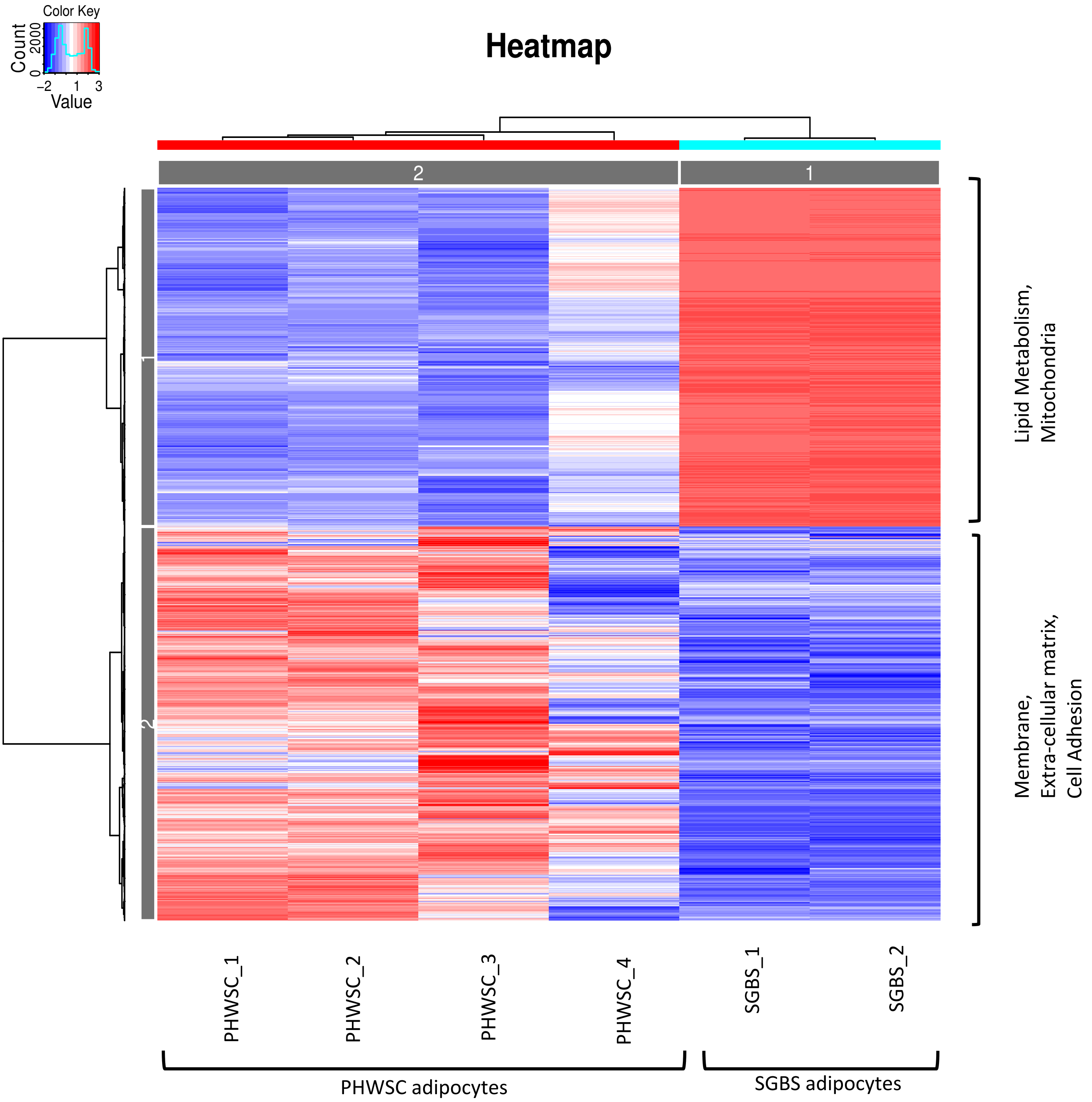

Supplementary figure S1: Heat map clustering differentially expressed genes either upregulated (red) or downregulated (blue) between SGBS and PHWSC adipocytes with FDR < 0.05. For PHWSC adipocytes in RNA-Seq, experiments were performed in cells derived from 4 different donors (PHWSC_1 to PHWSC_4, n=4). For SGBS adipocytes, experiments were performed in duplicates (SGBS_1 and SGBS_2) from the same SGBS adipocyte.

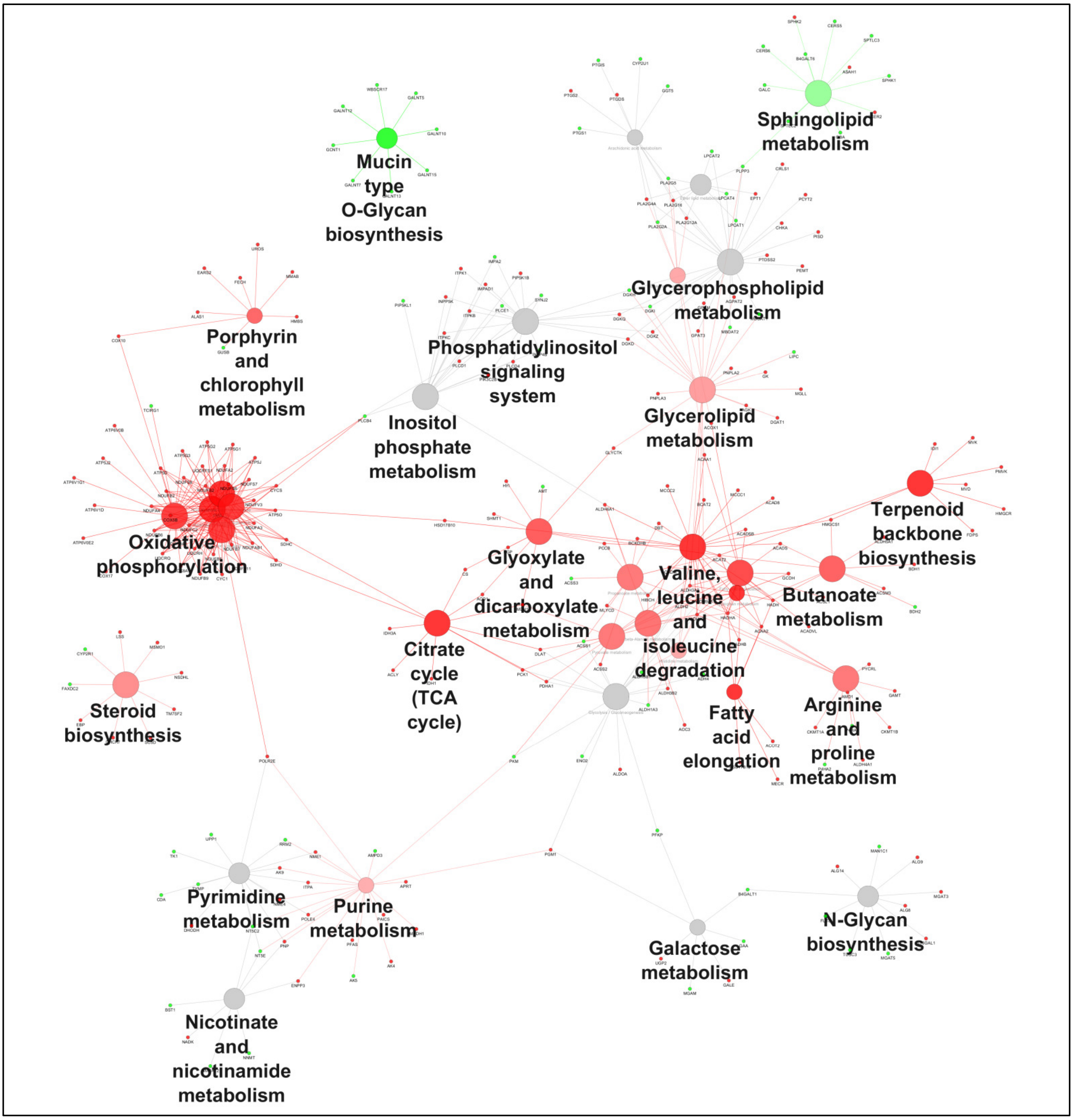

Supplementary figure S2: Enriched KEGG metabolic pathways in SGBS adipocytes compared to PHWSC adipocytes. Red colored nodes denotes pathways enriched in upregulated set of genes, green colored nodes denotes pathways enriched in downregulated set of genes and grey colored nodes denotes pathways enriched in both up and down regulated set of genes. Darker shade represents higher percentage of annotated genes in a particular pathway and larger size of the node shows term significance after Bonferroni correction. Pathway network was built in ClueGo and CluePedia Cytoscape apps. Kappa score was used to interconnect and functionally group the pathways. Significance was capped at $P < 0.05$ (post Bonferroni correction). Advanced settings used were as follows: selection criteria for the terms that have associated genes from cluster 1 and cluster 2: min 2 genes/term and minimum 4% from all the genes associated with the term, specificity set to 66%, GO term grouping, fix group coloring and Kappa Score grouping with 3 terms in initial group and 50% overlap for groups to merge.

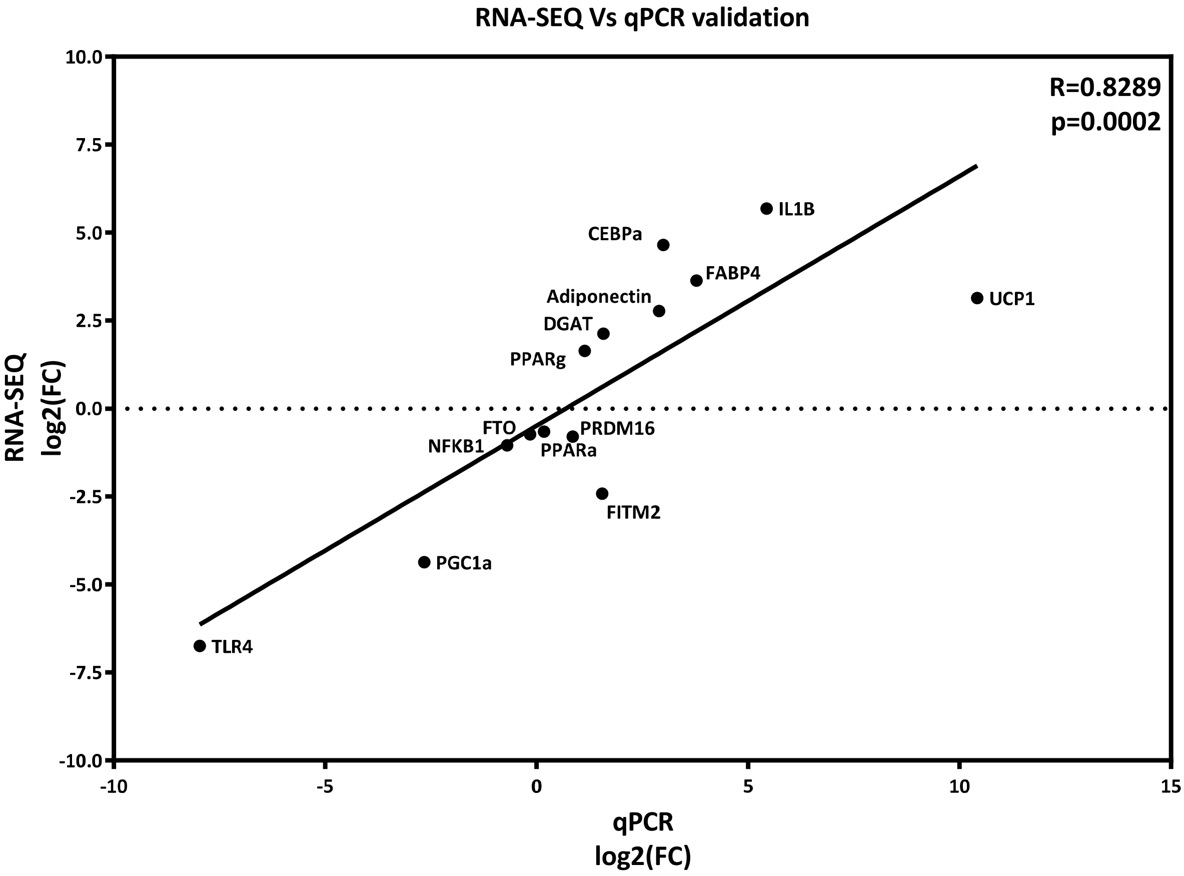

Supplementary figure S3: Correlation between gene expressions (SGBS vs PHWSC adipocytes) quantified using RNA-Seq and qRT-PCR methods in mature adipocytes. For the qPCR experiments involving PHWSC adipocytes, experiments were performed in duplicates from cells derived from 3 different donors. For SGBS adipocytes, qPCR experiments were performed in duplicates during 3 independent experiments from the same SGBS adipocyte. Sample size for RNA-Seq experiment have been mentioned in Fig 1. Pearson's correlation test was used to test for significance.

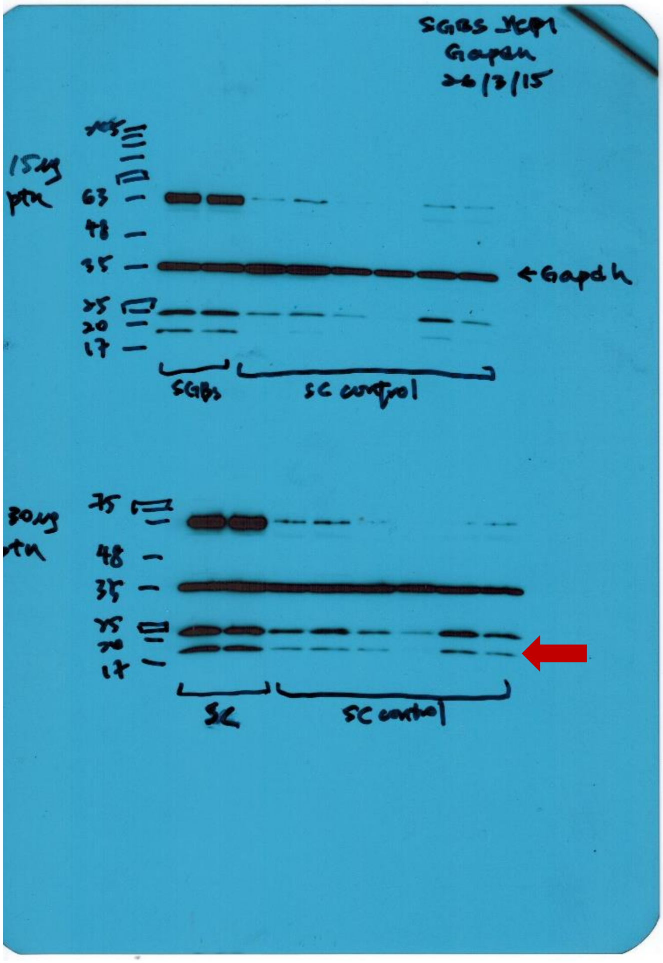

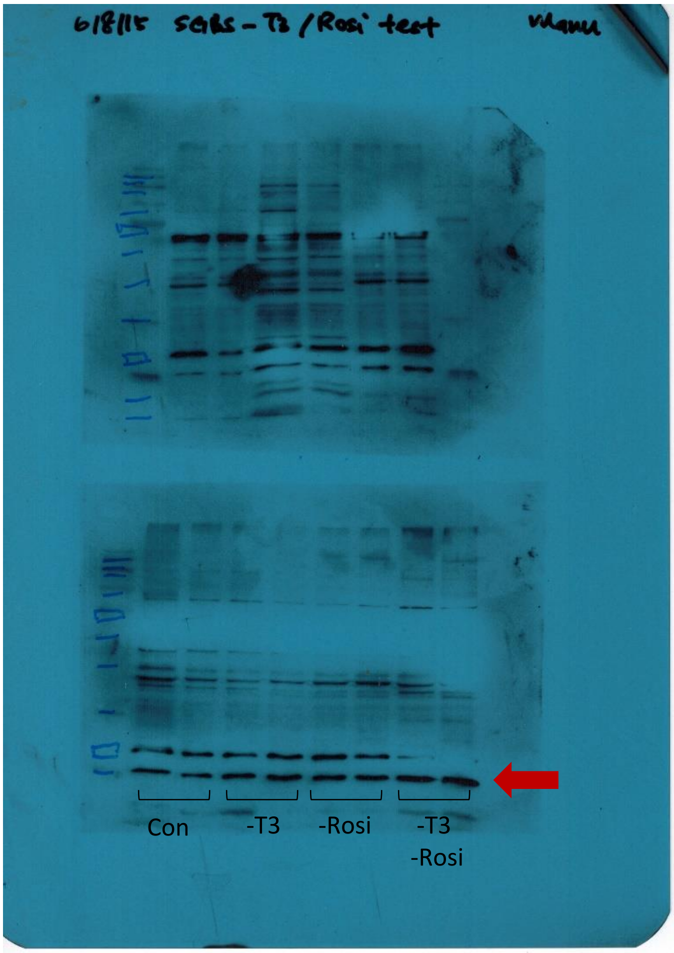

Supplementary figure S4: Full-length blots of UCP1 protein expression in SGBS and PHWSC adipocytes. Left panel: UCP1 protein expression in differentiated SGBS and PHWSC adipocytes. Right panel: UCP1 protein expression in SGBS adipocytes differentiated in medium depleted with either T3 or rosiglitazone or both.

Realtime qPCR	
**Gene**	**Primer sequence (5' – 3')**
FITM2	FP: CTGCCTTTCATTGCCCTCA
	RP: CTGCTTGCTCTGGTGTTCCTT
PPARγ	FP: AGACATTCAAGACAACCTGCTACAA
	RP: GGAGCAGCTTGGCAAACAG
PPARα	FP: CGATCTAGAGAGCCCGTTATCTGA
	RP: TGTCCCCGCAGATTCTACATT
CEBPα	FP: GAGGGACCGGAGTTATGACAAG
	RP: CGCACATTCACATTGCACAA
IL18	FP: ATCGCTTCCTCTCGCAACAA
	RP: ATCGCTTCCTCTCGCAACAA
IL1β	FP: TGAGCTCGCCAGTGAAATGA
	RP: AGATTCGTAGCTGGATGCCG
TLR4	FP: AGCGAGCCACGCATTCAC
	RP: GCCATGGCTGGGATCAGA
TNFα	FP: TCCCCTGCCCCAATCC
	RP: CCAATTCTCTTTTTGAGCCAGAA
NFκB1	FP: GGCTACACCGAAGCAATTGAA
	RP: CAGCGAGTGGGCCTGAGA
PGC1α	FP: GGTCTCTCCTTGCAGCACAAG
	RP: CTGGGATGACCGAAGTGCTT
Leptin	FP: CGGAGAGTACAGTGAGCCAAGA
	RP: CGGAATCTCGCTCTGTCATCA
DIO2	FP: TTTTCTCCAACTGCCTCTTCCT
	RP: CATGCGCCGCCACTCT
UCP1	FP: GGCTTCAGCGGCAAATCA
	RP: GAACTCCTGGACCGTGTCGTA
FTO	FP: CATGCCAGGTGCCAGTCA
	RP: GGCATCGAAGCATCATCCTT
ADIPOQ	FP: TGCCCAAAGAGGAGAGAGGAA
	RP: TCAGAAACAGGCACACAACTCA
FABP4	FP: CATAAAGAGAAAACGAGAGGATGATAAA
	RP: CCCTTGGCTTATGCTCTCTCA
DGAT1	FP: ACCTCATCTGGCTCATCTTCTTCTA
	RP: CCCGGTCTCCAAACTGCAT
PRDM16	FP: AGACACCTGAGGACGCACACT
	RP: GCTGGTCCCCAGGTGGTT
GAPDH	FP: GCCCCCGGTTTCTATAAATTG
	RP: GTCGAACAGGAGGAGCAGAGA
**Mitochondrial DNA content**	
**Gene**	**Sequence (5' – 3')**
Mitochondrial DNA	**Primers**
	FP: CACCCAAGAACAGGGTTTGT
	RP: TGGCCATGGGTATGTTGTTAA
	**Probe**
	TTACCGGGCTCTGCCATCT (5'-FAM, 3'-TAMRA)

**Nuclear DNA**	**Primers**
	FP: TAGAGGGACAAGTGGCGTTC
	RP: CGCTGAGCCAGTCAGTGT
	**Probe**
	AGCAATAACAGGTCTGTGATG (5'-FAM, 3'-TAMRA)

Gene symbol	Log FC (SGBS vs PHWSC)	Fold Change (SGBS vs PHWSC)	P value	FDR
NEUROG3	9.472	710.004	4.26E-20	3.87E-17
SLC22A12	9.378	665.564	6.00E-19	3.89E-16
NOTO	8.864	465.809	4.42E-26	8.08E-23
ALDH3B2	8.451	349.907	1.58E-50	2.15E-46
LY86-AS1	8.112	276.621	2.48E-28	8.43E-25
FCN1	8.106	275.447	4.03E-35	2.74E-31
MIR7162	7.273	154.637	3.35E-15	8.14E-13
LINC01070	7.262	153.524	4.16E-15	9.59E-13
OPRK1	7.218	148.855	7.90E-18	3.45E-15
KRT79	7.014	129.249	3.98E-10	2.92E-08
KLHDC7A	6.957	124.28	2.71E-13	4.39E-11
CD96	6.541	93.091	4.82E-29	2.19E-25
MC5R	6.536	92.774	7.40E-17	2.46E-14
NA	6.494	90.113	7.74E-14	1.51E-11
FOXE1	6.436	86.601	3.62E-11	3.60E-09
FFAR4	6.353	81.751	3.67E-18	1.85E-15
DEFB132	6.349	81.529	1.43E-12	1.96E-10
RAB19	6.287	78.087	1.83E-12	2.40E-10
NA	6.238	75.48	1.66E-12	2.21E-10
CA4	6.059	66.663	9.43E-16	2.52E-13
FAR2	6.037	65.652	5.35E-12	6.50E-10
CSF2RA	6.01	64.448	1.21E-17	4.75E-15
MGAT3	5.994	63.752	1.81E-16	5.37E-14
SLC6A12	5.97	62.694	2.55E-12	3.21E-10
CD52	5.958	62.18	5.33E-17	1.86E-14
ECHDC3	5.923	60.661	1.63E-18	9.24E-16
CTNNA3	5.788	55.255	1.05E-10	9.19E-09
IL1B	5.685	51.443	1.26E-15	3.29E-13
NA	5.681	51.307	8.55E-14	1.64E-11
LOC101929541	5.648	50.147	4.38E-10	3.20E-08
OASL	5.594	48.311	8.39E-17	2.72E-14
CITED1	5.591	48.185	2.71E-10	2.15E-08
PCK1	5.485	44.791	3.95E-07	1.08E-05
ABHD12B	5.467	44.219	2.85E-09	1.64E-07
FAM46C	5.453	43.793	1.82E-17	6.51E-15
LOC284837	5.446	43.603	5.27E-10	3.79E-08
LOC101926970	5.444	43.543	2.37E-27	6.46E-24
ESR2	5.405	42.369	2.96E-13	4.73E-11
NA	5.394	42.057	1.09E-16	3.45E-14
CDH22	5.391	41.964	3.56E-09	1.98E-07
MIR642B	5.353	40.881	1.15E-09	7.58E-08
LOC100129129	5.248	38.012	1.96E-11	2.07E-09
KRT77	5.235	37.649	5.35E-09	2.75E-07
ABCC6	5.217	37.202	1.80E-12	2.38E-10
AKAP5	5.2	36.768	5.62E-19	3.82E-16
STX11	5.186	36.406	9.45E-24	1.17E-20

LINC00278	5.131	35.03	8.16E-09	3.91E-07
GPR34	5.061	33.389	7.91E-07	1.97E-05
GIPR	5.021	32.472	2.44E-12	3.10E-10
NA	5.014	32.322	2.20E-09	1.33E-07
DBNDD1	4.98	31.553	7.25E-15	1.62E-12
PPP1R1B	4.944	30.781	4.03E-07	1.09E-05
DTX1	4.941	30.709	1.13E-12	1.59E-10
HEATR4	4.919	30.248	2.86E-11	2.95E-09
CPA2	4.911	30.083	1.14E-10	9.96E-09
RERGL	4.857	28.976	3.92E-10	2.90E-08
SEMA3G	4.805	27.957	3.00E-10	2.33E-08
SULT1A2	4.805	27.951	1.53E-08	6.74E-07
NA	4.765	27.181	4.50E-08	1.69E-06
NHLH1	4.732	26.582	1.15E-12	1.59E-10
CASZ1	4.694	25.89	9.69E-12	1.09E-09
NA	4.687	25.756	8.11E-06	0.000137031
BBOX1	4.676	25.561	1.22E-08	5.62E-07
CEBPA	4.652	25.135	8.67E-16	2.36E-13
GRAP	4.64	24.937	1.90E-07	5.79E-06
MAL2	4.578	23.885	5.74E-08	2.11E-06
GPR37	4.573	23.804	3.87E-10	2.88E-08
SLC29A4	4.52	22.937	2.40E-09	1.43E-07
PDE1B	4.478	22.284	1.13E-05	0.000181322
PKN2-AS1	4.429	21.538	3.07E-08	1.20E-06
SLC17A8	4.423	21.445	8.70E-07	2.14E-05
PPP1R1A	4.361	20.552	2.49E-07	7.28E-06
SSTR3	4.343	20.296	1.71E-08	7.31E-07
NA	4.341	20.267	6.09E-11	5.72E-09
FZD9	4.33	20.112	3.29E-08	1.28E-06
NA	4.284	19.477	2.07E-07	6.24E-06
EPHA5	4.256	19.103	6.07E-08	2.21E-06
CXCL14	4.253	19.066	3.97E-08	1.51E-06
LIPM	4.252	19.049	2.79E-10	2.19E-08
NMUR1	4.246	18.97	1.76E-05	0.000259739
PLEKHA7	4.243	18.932	1.96E-11	2.07E-09
JAG2	4.233	18.802	3.02E-10	2.34E-08
KAL1	4.223	18.673	5.03E-13	7.44E-11
SULT1A1	4.186	18.199	3.94E-12	4.87E-10
CKMT1B	4.184	18.181	7.87E-06	0.00013381
SCN7A	4.163	17.91	3.61E-05	0.000460423
RBM20	4.161	17.892	1.69E-08	7.29E-07
TINCR	4.154	17.805	9.26E-09	4.35E-07
SCAMP5	4.144	17.678	3.88E-08	1.49E-06
NA	4.141	17.644	0.000201636	0.001879357
RAB38	4.132	17.532	1.83E-11	1.96E-09
SLC7A10	4.127	17.467	4.79E-06	8.87E-05
SLC16A14	4.11	17.262	1.85E-14	3.88E-12
TSPAN13	4.099	17.136	1.03E-11	1.14E-09
EGFLAM	4.089	17.024	7.47E-08	2.67E-06
TPPP	4.064	16.731	6.82E-06	0.000118471

LINC00974	4.055	16.622	9.66E-07	2.33E-05
GSTM1	4.036	16.409	3.07E-05	0.00040352
DMTN	4.029	16.33	5.97E-16	1.66E-13
NR1H3	4.028	16.315	3.12E-06	6.23E-05
RP1	4	16.003	0.000191456	0.001804679
DUSP8	3.996	15.957	4.78E-09	2.53E-07
EYS	3.99	15.891	6.39E-07	1.65E-05
SORL1	3.985	15.839	6.44E-09	3.16E-07
NNAT	3.969	15.66	1.43E-06	3.27E-05
HLA-DMA	3.952	15.477	2.16E-10	1.78E-08
C14orf180	3.933	15.275	6.39E-05	0.000727544
RIMS4	3.921	15.144	3.09E-11	3.16E-09
AGPAT9	3.92	15.14	1.62E-12	2.19E-10
KCNIP2	3.868	14.601	9.96E-05	0.001050978
GAS2L2	3.862	14.542	0.003366379	0.017048634
C2orf72	3.85	14.417	7.70E-06	0.000131328
LINC01139	3.843	14.35	7.74E-07	1.95E-05
ZFP92	3.831	14.231	1.46E-06	3.31E-05
LOC101929513	3.82	14.119	0.000846726	0.005761121
LOC284825	3.812	14.049	5.33E-09	2.75E-07
RET	3.804	13.972	1.22E-05	0.000193927
ZNF295-AS1	3.778	13.715	2.82E-07	8.06E-06
LPL	3.752	13.473	4.55E-05	0.000554388
FLT1	3.742	13.383	5.99E-09	2.98E-07
IGFBPL1	3.742	13.378	3.31E-07	9.27E-06
NA	3.725	13.223	3.43E-06	6.75E-05
LOC102724699	3.714	13.12	2.62E-05	0.000358118
FCHO1	3.703	13.027	3.78E-05	0.00048006
NA	3.703	13.02	1.39E-09	8.98E-08
ITGA6	3.701	13.008	7.87E-19	4.87E-16
ACTG2	3.686	12.87	2.44E-06	5.08E-05
MESP1	3.655	12.596	3.41E-06	6.72E-05
NA	3.647	12.524	0.000244563	0.002180877
ADIPOQ	3.637	12.445	0.000410674	0.003297019
ITIH1	3.612	12.231	9.37E-05	0.001000858
ADRB2	3.608	12.19	6.00E-05	0.000690743
HILPDA	3.602	12.141	1.02E-12	1.45E-10
LOC728084	3.596	12.092	2.23E-06	4.72E-05
FAM151A	3.576	11.926	0.002241889	0.012441936
KRT14	3.574	11.911	2.18E-06	4.65E-05
MYEF2	3.566	11.841	3.43E-15	8.19E-13
LIPE	3.559	11.786	0.000609624	0.004464887
AQP3	3.559	11.782	1.35E-08	6.10E-07
PDE3B	3.558	11.777	0.000221417	0.002020822
NA	3.541	11.638	1.12E-06	2.65E-05
LGALS12	3.539	11.626	0.000307186	0.002615889
RASGRF2	3.538	11.616	2.84E-08	1.12E-06
PEMT	3.524	11.506	4.88E-11	4.71E-09
TMOD1	3.524	11.503	1.95E-06	4.20E-05
MMD	3.511	11.398	2.57E-07	7.46E-06

TRABD2A	3.502	11.328	9.40E-20	7.52E-17
STAR	3.499	11.31	0.000677713	0.004856407
RASD1	3.489	11.228	5.49E-09	2.81E-07
AQP11	3.463	11.026	8.02E-09	3.87E-07
CCDC85C	3.456	10.973	2.25E-09	1.36E-07
REEP6	3.45	10.926	1.01E-06	2.42E-05
GPBAR1	3.446	10.901	2.66E-05	0.000362272
MPP7	3.444	10.884	7.66E-08	2.72E-06
MAPK8IP2	3.438	10.839	2.75E-06	5.62E-05
ANGPTL4	3.433	10.802	9.01E-13	1.30E-10
ADORA1	3.433	10.802	1.02E-09	6.80E-08
SYN2	3.402	10.572	0.001795485	0.010456553
KCNK7	3.399	10.547	5.48E-06	9.92E-05
SERPINI1	3.397	10.535	1.52E-13	2.65E-11
LOC101929595	3.393	10.506	0.000511771	0.003888434
LOC100506368	3.393	10.502	2.36E-08	9.66E-07
PALMD	3.386	10.457	6.01E-07	1.58E-05
HRASLS5	3.384	10.439	0.000185531	0.001759376
TM7SF2	3.375	10.377	4.63E-05	0.00056169
MANEAL	3.372	10.35	7.13E-10	4.90E-08
NA	3.353	10.216	4.61E-05	0.00056042
PLP1	3.329	10.052	1.74E-06	3.81E-05
STBD1	3.328	10.044	1.68E-05	0.000250823
SYNGR2	3.328	10.044	2.03E-08	8.46E-07
GRB14	3.326	10.028	7.53E-06	0.00012881
PTGDS	3.315	9.954	3.74E-12	4.67E-10
C19orf80	3.309	9.911	0.003818537	0.018717425
ALDH1L1-AS2	3.309	9.907	4.34E-05	0.000537045
ADCY5	3.298	9.833	9.11E-08	3.16E-06
HOXB6	3.293	9.8	8.56E-15	1.88E-12
EPHB2	3.289	9.771	4.54E-11	4.47E-09
ICA1	3.263	9.597	0.000564892	0.004209774
NA	3.258	9.568	6.90E-05	0.000777841
RHOU	3.253	9.532	4.76E-11	4.64E-09
PLIN5	3.248	9.503	0.001602939	0.009583827
RNF165	3.244	9.476	0.000631807	0.004595208
CCNB1	3.235	9.413	8.81E-20	7.49E-17
NA	3.221	9.323	6.80E-05	0.00076702
CKMT1A	3.215	9.288	0.005822782	0.025936633
LRBA	3.213	9.275	5.66E-13	8.29E-11
HAVCR1	3.207	9.233	2.22E-05	0.000312599
PFKFB3	3.203	9.209	2.43E-06	5.08E-05
CX3CL1	3.199	9.181	1.61E-05	0.000243809
ONECUT2	3.197	9.171	4.37E-05	0.000540662
RBM24	3.193	9.142	4.03E-09	2.18E-07
HIST2H2BE	3.192	9.136	1.17E-13	2.12E-11
PLEKHG6	3.178	9.049	0.000224665	0.002044975
NA	3.168	8.985	4.52E-05	0.000552514
PCDH19	3.162	8.953	0.000685496	0.004896709
NA	3.157	8.92	7.90E-06	0.000133978

LETM1	3.155	8.908	8.92E-08	3.10E-06
RDH5	3.155	8.905	1.67E-05	0.000249279
AOC4P	3.154	8.904	5.00E-05	0.00059735
CITED4	3.152	8.889	6.51E-06	0.000113913
UCP1	3.144	8.843	0.00918729	0.036961891
ADIPOQ-AS1	3.143	8.832	0.001378731	0.0084665
MOGAT1	3.14	8.814	0.000673166	0.004831457
NA	3.139	8.809	7.00E-06	0.000121211
GPD1	3.137	8.794	0.000669435	0.004825038
HES6	3.135	8.785	1.48E-06	3.33E-05
ANO5	3.132	8.764	0.003422993	0.0172455
PIP5K1B	3.13	8.753	0.000268215	0.002356271
CYP4F12	3.129	8.749	2.98E-05	0.000395138
LOC100996635	3.122	8.709	0.001200181	0.007536714
KCNB1	3.118	8.685	0.000300403	0.002572615
HIST1H2BG	3.108	8.62	9.59E-06	0.000158786
MKNK2	3.104	8.597	7.99E-09	3.87E-07
KIAA0922	3.098	8.563	3.05E-15	7.55E-13
RNF125	3.098	8.56	0.000168752	0.001627485
TECR	3.095	8.542	8.93E-07	2.18E-05
STK26	3.093	8.535	1.91E-13	3.24E-11
PLCXD1	3.08	8.459	1.44E-06	3.27E-05
FAM213A	3.08	8.455	0.000645906	0.004680236
KLB	3.074	8.422	0.0003655	0.002998025
GLYAT	3.074	8.419	2.05E-05	0.000293444
DDIT4L	3.071	8.406	6.18E-12	7.25E-10
LOC286297	3.063	8.358	0.010690027	0.041488758
NA	3.061	8.348	1.00E-07	3.38E-06
GATA2	3.056	8.319	1.17E-05	0.000186712
SLC27A3	3.046	8.259	1.01E-07	3.38E-06
PLTP	3.043	8.244	6.24E-09	3.09E-07
MRAP	3.041	8.233	0.003430617	0.017277511
MVD	3.041	8.233	1.69E-05	0.000251925
PKD2L1	3.036	8.205	0.000868482	0.005862252
EPB41	3.036	8.203	1.05E-13	1.96E-11
OLAH	3.032	8.181	8.83E-08	3.08E-06
MMP15	3.031	8.173	7.41E-10	5.04E-08
HEYL	3.031	8.173	4.52E-05	0.000552514
AZGP1	3.03	8.166	0.003241568	0.016514885
MTRNR2L1	3.02	8.114	5.23E-05	0.000619239
CCDC85B	3.017	8.094	1.16E-16	3.59E-14
TST	3.008	8.044	1.43E-08	6.34E-07
PPM1H	3.006	8.032	2.53E-06	5.23E-05
LOC101929726	2.99	7.946	0.008876142	0.035989441
CXCR2	2.985	7.918	0.001161768	0.007353178
Mar-01	2.981	7.894	0.001039045	0.006742645
SH2D3C	2.98	7.89	0.000114404	0.001179404
NA	2.972	7.848	0.005654662	0.025395592
FERMT1	2.972	7.847	5.24E-10	3.79E-08
AP3B2	2.969	7.829	0.000499238	0.003805954

PLIN4	2.968	7.824	0.001665777	0.009859891
RGAG1	2.963	7.798	7.08E-09	3.45E-07
ALDH1L1	2.963	7.796	0.005126974	0.023475053
SV2A	2.962	7.791	1.39E-07	4.45E-06
NUDT14	2.959	7.776	6.23E-06	0.000109473
HCAR3	2.954	7.747	0.002250105	0.012467682
ARHGAP20	2.944	7.693	9.23E-09	4.34E-07
ABCA3	2.943	7.688	8.41E-11	7.52E-09
CASQ2	2.942	7.687	0.005731863	0.025666071
PKP2	2.939	7.669	1.08E-07	3.58E-06
SSC4D	2.93	7.621	1.81E-07	5.59E-06
TRHDE	2.929	7.615	1.78E-08	7.54E-07
CLMN	2.915	7.541	0.000705445	0.005020758
UBASH3B	2.908	7.504	3.15E-13	4.98E-11
NA	2.906	7.496	1.23E-05	0.00019482
FLVCR2	2.905	7.492	4.52E-05	0.000552514
KLHL25	2.902	7.474	5.94E-08	2.17E-06
HPGD	2.901	7.471	0.00717413	0.03053508
PANK1	2.891	7.418	1.88E-09	1.18E-07
BRINP2	2.886	7.39	0.000181531	0.001727888
MLYCD	2.885	7.389	1.67E-09	1.06E-07
NA	2.88	7.364	0.004457086	0.021023232
RAC3	2.879	7.358	2.32E-05	0.000324341
CALB2	2.877	7.348	0.000132503	0.001332665
C16orf89	2.877	7.348	4.67E-05	0.000565378
NA	2.873	7.324	0.003818456	0.018717425
FAM49B	2.872	7.322	1.08E-13	1.98E-11
ECHDC1	2.864	7.279	8.75E-06	0.00014656
STOX1	2.861	7.263	0.000339265	0.002820235
CAMK1	2.859	7.255	2.43E-06	5.08E-05
HIP1R	2.855	7.233	1.65E-08	7.13E-07
PLS1	2.853	7.223	2.31E-05	0.000323118
TSPAN15	2.851	7.214	3.04E-06	6.13E-05
MAP7	2.847	7.195	8.70E-07	2.14E-05
TSPAN33	2.843	7.175	1.53E-05	0.000234914
NA	2.839	7.154	2.74E-05	0.000370469
ADPGK-AS1	2.83	7.111	2.33E-05	0.000324986
SAMD5	2.83	7.11	0.000398794	0.003224477
CSPG5	2.828	7.099	0.002902752	0.015175049
FGR	2.819	7.057	0.006297321	0.027510094
MEST	2.815	7.037	9.00E-05	0.000966338
FRMD3	2.813	7.026	2.49E-06	5.16E-05
LSR	2.81	7.015	6.91E-06	0.000119982
AOC3	2.808	7.004	0.000756363	0.00529454
NA	2.801	6.971	1.12E-07	3.70E-06
VAT1L	2.801	6.969	1.49E-05	0.000229892
C11orf48	2.796	6.945	6.35E-07	1.65E-05
CNTFR	2.795	6.942	0.005895776	0.026150493
MAPK10	2.794	6.937	0.000136987	0.00136866
ALG9	2.793	6.931	1.72E-10	1.46E-08

GPT	2.793	6.929	0.002881727	0.01509994
LPCAT3	2.782	6.88	0.000495586	0.003784476
FABP4	2.774	6.842	0.005949489	0.02636296
SPTBN2	2.774	6.84	4.19E-06	7.97E-05
HOXB8	2.767	6.808	1.79E-09	1.13E-07
DTX4	2.767	6.808	1.37E-16	4.14E-14
NA	2.766	6.802	0.002986538	0.015517682
LOC101926941	2.765	6.798	3.90E-05	0.000492146
ALDH5A1	2.758	6.765	2.42E-07	7.10E-06
RPH3AL	2.752	6.737	1.58E-05	0.000241227
SLC25A1	2.751	6.733	0.000260954	0.002298419
LFNG	2.747	6.714	3.03E-08	1.19E-06
AQP7	2.747	6.711	0.003035312	0.015700862
SDPR	2.743	6.697	1.38E-08	6.17E-07
FZD5	2.737	6.667	2.18E-07	6.51E-06
LAIR1	2.735	6.66	0.0013072	0.008100353
PLEKHH1	2.725	6.611	0.001185232	0.007472285
THEMIS2	2.724	6.608	0.000540375	0.004060419
ENPP3	2.724	6.607	0.000489894	0.003751532
FAT2	2.721	6.594	0.000541155	0.004064035
NA	2.719	6.583	0.001908113	0.010960573
NA	2.716	6.571	1.90E-06	4.10E-05
GPER1	2.714	6.561	2.06E-05	0.000293818
KLHDC8B	2.71	6.542	4.45E-05	0.000547088
NA	2.71	6.542	0.013605897	0.049865082
TLE1	2.707	6.529	1.58E-11	1.72E-09
PDZD2	2.707	6.528	0.001615227	0.009622434
RASIP1	2.704	6.516	1.83E-05	0.000269618
LRRIQ3	2.702	6.507	8.42E-05	0.000913368
FYB	2.692	6.461	0.000231023	0.002086102
KANSL1-AS1	2.684	6.427	1.06E-05	0.000172853
SGK2	2.68	6.408	0.004057953	0.019577363
FADS1	2.67	6.365	0.000215849	0.001985985
GFOD1	2.668	6.358	1.53E-05	0.000234272
FABP5	2.668	6.355	0.001267577	0.007897983
NA	2.664	6.34	0.000398547	0.003224391
ZBTB42	2.662	6.328	3.02E-05	0.00039784
HADH	2.659	6.316	0.001547378	0.009298736
DGCR6	2.651	6.281	8.70E-05	0.000937113
THRSP	2.65	6.278	0.011653087	0.044229934
PDP2	2.644	6.249	2.38E-06	5.00E-05
TMEM56	2.641	6.239	2.03E-06	4.35E-05
CIDEC	2.641	6.239	0.00876131	0.035642422
SYT12	2.641	6.236	0.000346455	0.002860776
RBP4	2.627	6.176	0.006499325	0.028202428
TOX	2.626	6.171	0.000282583	0.002458691
NA	2.625	6.17	5.40E-05	0.000633726
GPLD1	2.62	6.148	8.53E-05	0.000924546
NA	2.62	6.147	2.40E-05	0.000333237
NA	2.617	6.136	0.00530009	0.024073308

ASS1	2.615	6.124	1.33E-07	4.27E-06
ACSM3	2.609	6.101	3.41E-06	6.72E-05
YBX2	2.607	6.094	0.00011761	0.001204019
PAX8	2.606	6.087	0.000684588	0.004894765
IQSEC3	2.605	6.086	1.82E-06	3.97E-05
PDE8B	2.602	6.07	0.012249579	0.045946052
HEBP2	2.6	6.064	3.56E-05	0.000455264
L2HGDH	2.599	6.057	8.02E-06	0.00013589
PRRT3-AS1	2.595	6.042	0.000445101	0.003505169
RHPN2	2.595	6.041	2.44E-05	0.000337623
TMEM132C	2.591	6.027	0.008033593	0.033394687
ACAP1	2.59	6.023	0.003836672	0.018780371
SOX6	2.587	6.01	0.000913489	0.00609351
TACC2	2.573	5.95	1.32E-13	2.37E-11
PPARGC1B	2.572	5.947	0.000578136	0.004282674
GLDN	2.565	5.918	5.58E-06	0.000100616
ITGA7	2.562	5.907	0.002299466	0.012627579
MFSD9	2.55	5.858	2.49E-11	2.60E-09
NEDD4L	2.549	5.852	2.99E-05	0.000395138
HYI	2.547	5.846	6.29E-11	5.86E-09
HIST1H1C	2.547	5.844	3.46E-10	2.63E-08
NA	2.544	5.831	4.24E-05	0.000526506
DBI	2.54	5.814	0.000401821	0.003241501
AKR1C2	2.538	5.809	0.000229336	0.002075765
HCAR2	2.527	5.765	0.008540148	0.034983243
ADAMTS15	2.527	5.764	2.78E-05	0.000374748
PREX1	2.522	5.744	2.39E-06	5.00E-05
ACER3	2.514	5.712	2.50E-05	0.000342978
PITPNC1	2.506	5.682	4.46E-14	8.80E-12
SYNGR1	2.505	5.675	8.31E-07	2.05E-05
GAS2L3	2.504	5.674	2.02E-05	0.000289388
PECR	2.504	5.671	0.001565609	0.009381245
ABHD15	2.495	5.638	1.91E-08	8.03E-07
PEX19	2.489	5.613	2.14E-05	0.00030346
NPTXR	2.489	5.613	4.38E-06	8.27E-05
HIST2H2AA4	2.486	5.603	0.000203742	0.001895091
C11orf87	2.484	5.593	0.009921421	0.039247294
ACLY	2.482	5.586	0.001812858	0.010519586
TMC6	2.479	5.576	0.007573448	0.03187735
ELOVL3	2.477	5.569	0.002298526	0.012627579
UCP2	2.477	5.567	0.011436432	0.043690896
FZD4	2.472	5.549	1.32E-05	0.000207097
BTG2	2.46	5.503	1.54E-08	6.76E-07
EBP	2.459	5.499	0.000453247	0.00354674
SLC25A33	2.458	5.495	1.26E-07	4.08E-06
THEM6	2.458	5.495	0.00020615	0.001914873
TDRKH	2.455	5.483	3.53E-05	0.000450896
CELSR2	2.453	5.475	0.00119567	0.007522273
HES4	2.452	5.47	0.000382309	0.003120856
P2RX6	2.447	5.452	0.000324059	0.002728837

ACAA2	2.446	5.447	0.000660525	0.004768395
ACSF2	2.441	5.429	0.005718484	0.025623027
PNPLA2	2.439	5.422	0.00459514	0.021479957
KCNAB1	2.435	5.409	0.009093468	0.036686602
NA	2.432	5.398	0.000302807	0.002589938
ADAMTS4	2.432	5.396	1.94E-09	1.20E-07
BCKDHB	2.425	5.372	1.51E-06	3.39E-05
SREBF1	2.419	5.347	0.000319975	0.002697778
AMPH	2.416	5.336	2.62E-09	1.53E-07
SLC19A1	2.411	5.318	8.69E-06	0.000146015
ALDOA	2.41	5.315	5.94E-06	0.000105352
LOC399491	2.406	5.301	0.000291632	0.002514911
PDXK	2.404	5.291	7.51E-07	1.90E-05
SORBS1	2.403	5.29	0.000524391	0.003968804
FAM195A	2.401	5.282	1.41E-06	3.23E-05
ACY1	2.4	5.279	4.80E-05	0.000578332
C17orf53	2.399	5.275	2.21E-05	0.000311606
ZNF77	2.397	5.268	1.40E-07	4.45E-06
TMEM120A	2.394	5.257	2.33E-07	6.87E-06
HIST1H2BG	2.393	5.253	7.73E-05	0.000850211
HOXB-AS1	2.39	5.243	8.19E-06	0.000138351
CAV2	2.39	5.242	2.20E-06	4.66E-05
EXOC6	2.387	5.231	9.04E-08	3.14E-06
P2RY12	2.386	5.228	0.005962519	0.02639491
ACOT2	2.385	5.224	0.000712174	0.005055434
NXPH4	2.385	5.224	2.06E-07	6.23E-06
PLA2G4A	2.38	5.206	1.59E-05	0.000241861
G0S2	2.379	5.201	0.001822474	0.010557781
DUSP6	2.378	5.199	2.62E-05	0.000358118
TUBB2A	2.372	5.178	1.41E-07	4.47E-06
SLC25A18	2.369	5.167	0.007055925	0.030127715
C12orf45	2.367	5.157	1.44E-07	4.55E-06
NMB	2.365	5.151	2.88E-05	0.000385312
C10orf90	2.364	5.147	0.000473771	0.003663112
LOC158960	2.362	5.139	0.000896334	0.006017423
SCD	2.356	5.119	0.004562053	0.021377555
AGPAT2	2.35	5.099	0.004137982	0.019855308
IDH1	2.347	5.089	0.000109997	0.001144375
RILP	2.343	5.075	1.28E-05	0.000202186
CDKN2C	2.341	5.067	0.000353106	0.002908637
GLYCTK	2.339	5.058	0.001583328	0.009479068
CDKN2A	2.337	5.052	2.09E-07	6.29E-06
TMEM164	2.329	5.026	7.55E-05	0.000835615
TMEM246	2.329	5.024	1.24E-06	2.90E-05
MAPT	2.327	5.018	0.000213831	0.001970086
IGF2BP3	2.326	5.013	4.77E-06	8.87E-05
XRCC6BP1	2.317	4.982	2.59E-06	5.32E-05
KIAA1958	2.315	4.977	5.69E-06	0.000102123
IDI1	2.315	4.976	7.34E-05	0.000814372
NSDHL	2.315	4.975	0.002225309	0.012370098

COL5A3	2.31	4.958	1.28E-06	2.98E-05
TXLNG	2.309	4.955	1.64E-06	3.62E-05
TLCD1	2.308	4.952	3.65E-07	1.01E-05
TSHR	2.308	4.95	0.000284597	0.002473048
SLC25A22	2.306	4.946	7.04E-06	0.0001216
APMAP	2.306	4.943	1.65E-05	0.000247459
MGST3	2.303	4.933	6.10E-06	0.000107675
HOXB5	2.3	4.923	5.92E-08	2.17E-06
ACSL1	2.293	4.902	0.002592581	0.013917096
GK	2.293	4.9	3.00E-06	6.06E-05
ISOC1	2.289	4.886	0.000332242	0.002785675
EFNB2	2.288	4.884	0.000328073	0.002754113
NA	2.285	4.873	0.007380807	0.031250163
HOXB9	2.285	4.873	0.00218955	0.012226263
ACADM	2.285	4.872	2.23E-05	0.000313283
CHP1	2.283	4.867	1.95E-05	0.000282423
RGL3	2.282	4.863	0.000774835	0.005374084
C20orf27	2.282	4.862	1.79E-05	0.000263688
KIF21A	2.279	4.853	1.37E-07	4.38E-06
KLHL10	2.275	4.84	0.00522137	0.02381308
TUBB2B	2.271	4.827	1.66E-05	0.000248982
MECR	2.269	4.82	3.67E-05	0.000467285
NA	2.266	4.81	0.0101136	0.039741803
LYPD1	2.265	4.806	0.005424354	0.024523125
C19orf12	2.263	4.8	4.49E-05	0.000551043
FAM89A	2.259	4.787	1.98E-05	0.000285517
OAS1	2.259	4.786	0.002257201	0.012492137
ANK3	2.256	4.778	3.49E-06	6.83E-05
BOK	2.256	4.775	4.55E-05	0.000554388
VKORC1L1	2.251	4.761	0.000308204	0.002622915
ADAM12	2.251	4.759	1.87E-07	5.75E-06
PGM1	2.248	4.752	4.07E-06	7.82E-05
TMEM256	2.247	4.748	8.77E-07	2.15E-05
ARL6IP1	2.245	4.739	7.78E-08	2.75E-06
ABHD5	2.244	4.735	4.12E-07	1.11E-05
GNG2	2.243	4.735	0.000230032	0.002078538
ALDH1B1	2.239	4.721	3.47E-05	0.000445088
HSDL2	2.236	4.712	0.000566066	0.004216214
KHK	2.236	4.709	0.000695421	0.004954601
ASIC1	2.234	4.704	1.63E-08	7.05E-07
MDH1	2.231	4.693	0.000115437	0.00118645
ZNF233	2.228	4.683	8.91E-05	0.000957132
NA	2.222	4.664	0.000542071	0.004066427
STOM	2.221	4.662	7.17E-05	0.000800304
ACSS2	2.22	4.66	0.011056123	0.042556784
ALDH9A1	2.217	4.648	1.68E-05	0.000250295
SAMM50	2.214	4.638	1.85E-05	0.000270131
TMED5	2.212	4.633	6.19E-07	1.61E-05
PRRT4	2.211	4.632	0.006038683	0.026662684
NA	2.208	4.621	0.000604005	0.004430892

ZNHIT1	2.206	4.613	8.46E-09	4.03E-07
WWC1	2.2	4.595	0.000644976	0.00467848
CCND3	2.197	4.585	5.62E-07	1.49E-05
PDK4	2.193	4.573	0.007024643	0.030012982
RETSAT	2.192	4.571	0.0022443	0.012450237
DHCR7	2.192	4.569	0.00055403	0.004135621
SLC47A1	2.189	4.561	0.001183255	0.007464872
LRRC8B	2.189	4.558	5.29E-06	9.63E-05
BAI2	2.175	4.517	0.000244956	0.002182949
NUDT8	2.174	4.512	0.003851451	0.018839159
HIVEP3	2.173	4.509	9.19E-05	0.000983435
APRT	2.17	4.5	2.25E-07	6.67E-06
TM4SF4	2.169	4.496	0.011640563	0.044206817
ITPKB	2.166	4.489	1.95E-09	1.20E-07
SLC27A1	2.164	4.481	1.02E-05	0.000167732
NA	2.164	4.481	0.000771046	0.005364213
NA	2.163	4.478	0.002487166	0.013457398
STAT5A	2.161	4.473	0.00030658	0.002615542
DIXDC1	2.157	4.459	5.46E-07	1.45E-05
ISPD	2.157	4.459	1.04E-05	0.000169654
GPR137C	2.154	4.449	0.000450863	0.003536223
GPAM	2.151	4.44	0.01092045	0.042145626
MCAM	2.15	4.437	0.004119073	0.019792495
RASL10B	2.15	4.437	0.004545435	0.021314361
PLEKHA8P1	2.138	4.403	0.000141258	0.001408236
PDHA1	2.137	4.398	0.000453937	0.003548058
CYCS	2.136	4.396	0.000294608	0.002535755
RBM38	2.135	4.394	3.26E-05	0.000424678
CD83	2.134	4.388	2.49E-05	0.000342862
HSD17B12	2.133	4.385	5.20E-05	0.000616392
DGAT1	2.128	4.371	0.000638063	0.004633274
PLA2G16	2.127	4.368	0.006706201	0.028970788
CASP7	2.126	4.365	2.78E-08	1.10E-06
ACADS	2.123	4.355	0.0044045	0.020832965
TPGS1	2.122	4.354	9.35E-07	2.27E-05
Mar-03	2.117	4.337	7.82E-05	0.000857938
HIST3H2A	2.116	4.336	0.001017017	0.006628145
LOC101926901	2.107	4.309	1.52E-06	3.41E-05
CD36	2.106	4.304	0.0013949	0.008549957
CRACR2B	2.105	4.303	0.004101595	0.019750354
BCYRN1	2.105	4.302	0.000235752	0.002117568
RCL1	2.105	4.301	2.67E-05	0.000363322
ITIH5	2.103	4.296	6.65E-07	1.71E-05
ETFA	2.103	4.295	7.04E-05	0.000788083
PPTC7	2.102	4.293	0.000119075	0.001216495
ABLIM2	2.1	4.288	0.006588499	0.028527584
CLUH	2.1	4.287	0.000932152	0.006196737
SCARB1	2.095	4.271	0.000896251	0.006017423
LDHD	2.093	4.265	0.010121928	0.039763047
CCNF	2.09	4.256	6.32E-06	0.000110895

LEPR	2.089	4.255	1.27E-06	2.97E-05
NEK8	2.087	4.25	1.10E-06	2.62E-05
WDR3	2.087	4.248	7.63E-07	1.92E-05
SNAI3-AS1	2.087	4.247	1.53E-05	0.000234272
SHMT1	2.085	4.242	0.011879201	0.044915856
ALDH3A2	2.082	4.234	6.65E-05	0.00075254
OSBPL11	2.082	4.234	0.002369908	0.012930915
ALAS1	2.078	4.222	8.09E-05	0.00088045
PDF	2.077	4.22	0.000453114	0.00354674
SH3RF2	2.072	4.203	0.001050974	0.006807072
CMTM7	2.071	4.201	2.48E-08	1.00E-06
NUDT19	2.07	4.198	1.10E-08	5.10E-07
TENM4	2.063	4.178	4.79E-06	8.87E-05
H19	2.061	4.174	5.59E-05	0.000650234
MVK	2.061	4.174	0.000481515	0.00371277
ETFB	2.055	4.156	0.000208476	0.001933894
MIEF1	2.054	4.152	5.11E-06	9.38E-05
FRMD4A	2.053	4.151	0.000135023	0.001352019
NAPRT	2.053	4.149	0.000183991	0.001747209
FITM2	2.052	4.148	0.00058609	0.004327459
TRIM6	2.052	4.148	5.70E-06	0.000102126
HMGCS1	2.052	4.147	0.000186683	0.001769068
NA	2.046	4.13	0.00611171	0.026897851
HIST1H2BJ	2.045	4.127	0.000205143	0.001906823
PEX11A	2.043	4.12	0.001797316	0.010456553
HMGCR	2.043	4.12	6.29E-05	0.000716474
HMGN5	2.042	4.119	1.01E-05	0.000165696
ECE2	2.038	4.107	0.000198901	0.001861515
ALDH4A1	2.035	4.097	0.000413363	0.0033147
SHBG	2.033	4.092	0.000443482	0.003497576
VILL	2.032	4.09	9.35E-07	2.27E-05
LPHN1	2.03	4.085	7.53E-05	0.000833695
LIMS1	2.028	4.078	1.14E-06	2.69E-05
BHMT2	2.028	4.078	5.66E-09	2.87E-07
CDKN1A	2.027	4.075	2.98E-05	0.000395084
ELOVL5	2.027	4.075	0.002798863	0.014762376
FAM171A2	2.026	4.074	8.63E-05	0.00093157
TMEM53	2.026	4.073	0.000804408	0.005539669
SLC1A3	2.023	4.064	0.000462392	0.003593695
PDK1	2.019	4.053	0.0060413	0.026665591
FHL1	2.019	4.052	3.96E-06	7.65E-05
MCCC2	2.018	4.051	0.000185043	0.001755976
PITX1	2.018	4.049	0.007988446	0.033274189
NUDT7	2.015	4.043	0.002108703	0.01186183
PRKACA	2.015	4.043	3.86E-06	7.47E-05
ZNF391	2.013	4.037	2.09E-07	6.28E-06
TYSND1	2.012	4.034	4.03E-06	7.77E-05
YARS2	2.011	4.031	1.18E-07	3.88E-06
ILVBL	2.011	4.03	4.81E-06	8.90E-05
HOXB-AS3	2.011	4.029	0.00162913	0.009689335

TJP2	2.008	4.022	0.000613114	0.00448562
ME2	2.008	4.022	8.83E-06	0.000147745
COL4A1	2.007	4.019	1.39E-05	0.000216383
GCSH	2.003	4.007	0.000332724	0.002786282
MRO	1.993	3.982	0.012980911	0.048066458
CPT2	1.992	3.977	4.68E-05	0.000566662
ABCC11	1.988	3.967	0.006484619	0.028156572
PHLDA3	1.986	3.962	4.27E-06	8.12E-05
PQLC1	1.985	3.959	4.99E-05	0.00059735
H2AFJ	1.984	3.957	3.47E-06	6.81E-05
GKAP1	1.983	3.952	0.000672266	0.004831457
CS	1.981	3.949	0.005783879	0.025822516
TXNDC16	1.981	3.947	9.33E-08	3.21E-06
HIBCH	1.979	3.941	3.48E-07	9.71E-06
AIFM2	1.976	3.934	0.002491742	0.013476799
DLAT	1.973	3.925	0.00834875	0.034395939
SGK223	1.972	3.923	0.001657401	0.009835988
TMEM135	1.969	3.916	0.004466209	0.021044753
RPP25	1.969	3.914	0.00010103	0.001064102
GLIPR2	1.966	3.906	7.52E-07	1.90E-05
ST6GAL1	1.966	3.906	0.000818775	0.005613042
SLC25A45	1.963	3.9	2.82E-07	8.06E-06
TOR4A	1.963	3.899	0.005817163	0.025928578
SH3BP1	1.963	3.898	0.000486639	0.003739236
NPR1	1.962	3.895	0.013073533	0.048340259
ETFDH	1.96	3.891	0.00084921	0.005774787
ACER2	1.957	3.882	0.000400956	0.003238107
PTPRF	1.955	3.877	0.011658787	0.044229934
GPATCH11	1.954	3.876	0.002888383	0.01512899
RAB20	1.953	3.871	0.008040961	0.033400913
SLC25A13	1.953	3.871	9.63E-06	0.000159185
MSMO1	1.95	3.865	0.000322143	0.002714375
TIPARP	1.948	3.857	3.14E-06	6.26E-05
HIST1H3A	1.943	3.846	0.002359749	0.012885818
ADAMTS12	1.935	3.823	5.91E-06	0.000105019
ACADVL	1.933	3.818	0.000424025	0.003386231
ACOX1	1.932	3.815	5.20E-05	0.000616052
CDC25A	1.931	3.813	0.003114903	0.01599532
LOC101930114	1.93	3.812	0.009170364	0.036909291
SDK1	1.93	3.81	0.011407791	0.043613814
MARS2	1.928	3.805	0.000200854	0.001873354
CRHR1-IT1	1.926	3.801	0.000294852	0.00253625
MPST	1.926	3.8	3.03E-06	6.11E-05
MRPL54	1.926	3.799	1.42E-06	3.26E-05
COA3	1.924	3.795	2.10E-07	6.30E-06
SYNM	1.923	3.793	0.000241678	0.002162236
MTHFD1	1.919	3.783	0.000682844	0.004888027
SLC29A1	1.919	3.782	0.000118779	0.001214382
PNPLA3	1.914	3.769	0.003325152	0.016883829
PIM3	1.911	3.762	2.90E-05	0.000386629

POT1	1.911	3.76	4.69E-09	2.49E-07
KCTD14	1.911	3.76	0.003477194	0.017473284
ACAT2	1.907	3.75	0.003517493	0.017572459
JUP	1.906	3.747	3.37E-05	0.000433682
RRP9	1.905	3.745	3.52E-05	0.000450403
OPLAH	1.904	3.742	9.46E-05	0.001008473
LOC100288181	1.901	3.733	0.001613107	0.009622434
GALE	1.899	3.73	0.000239632	0.002146749
MATN2	1.899	3.728	0.00073388	0.005171745
EFNB1	1.898	3.727	9.16E-08	3.16E-06
FLVCR1	1.898	3.726	0.000318528	0.002687247
CAT	1.895	3.72	0.002112733	0.011870382
HINT2	1.895	3.719	3.53E-05	0.000450896
PLCD4	1.894	3.717	0.001662642	0.009845621
CA5B	1.893	3.714	0.000602758	0.004424128
QPCT	1.89	3.706	1.95E-05	0.000282423
PEG10	1.889	3.703	0.002558284	0.013776463
MMAB	1.888	3.702	0.000581123	0.004299479
SORT1	1.888	3.701	0.000571915	0.004248155
PCYT2	1.886	3.697	0.004265451	0.020302293
DOCK11	1.884	3.691	0.000377323	0.003082003
CCDC69	1.882	3.687	0.001461326	0.008869637
SLC22A3	1.882	3.685	0.009979393	0.039396455
DARS2	1.88	3.681	1.70E-06	3.73E-05
HADHA	1.88	3.68	0.000553013	0.004130294
PQLC2L	1.876	3.671	4.37E-06	8.25E-05
PTPLB	1.874	3.666	6.66E-05	0.000752732
BEST1	1.873	3.663	9.87E-08	3.34E-06
FAM199X	1.872	3.661	2.01E-07	6.12E-06
NA	1.87	3.655	0.004225164	0.020141264
MAPK11	1.869	3.652	0.000384275	0.003131016
DCXR	1.868	3.651	0.001199798	0.007536714
PDHX	1.866	3.645	1.74E-05	0.000258034
HAS2	1.865	3.644	1.94E-06	4.17E-05
GRPEL1	1.865	3.643	6.95E-05	0.000781644
NA	1.865	3.641	0.005272917	0.023973889
PRADC1	1.864	3.641	7.58E-05	0.000836926
FRMD8	1.864	3.64	4.13E-06	7.91E-05
NA	1.862	3.636	0.003171462	0.016236739
PIK3C2B	1.857	3.624	2.39E-05	0.000332041
CENPW	1.857	3.622	0.000165183	0.001602143
NQO2	1.856	3.621	0.000438838	0.003480016
MRPL12	1.854	3.614	0.000124282	0.001261168
NR2F6	1.852	3.609	8.16E-07	2.03E-05
UQCRFS1	1.849	3.603	0.001431833	0.008721746
ASAH1	1.846	3.594	8.63E-05	0.000931711
ENOX1	1.841	3.581	0.000151131	0.001493306
TIMM8A	1.839	3.577	6.33E-05	0.000720895
ZCCHC3	1.838	3.575	0.003534015	0.017641554
TSKU	1.838	3.574	0.010577335	0.041159957

BDH1	1.837	3.572	0.00453665	0.021287837
RPS26	1.837	3.571	0.000405338	0.003261972
SLC25A4	1.835	3.568	3.92E-05	0.000494376
CADM3	1.834	3.565	2.40E-05	0.000333384
LOC100289098	1.831	3.557	0.008430279	0.034658379
SLC16A7	1.829	3.554	0.000470081	0.00364286
FANCE	1.829	3.553	5.55E-05	0.000647744
TALDO1	1.827	3.549	0.002313343	0.01268331
PYCRL	1.827	3.547	0.000131811	0.001326686
GPR146	1.825	3.544	0.001961301	0.011214025
PRDX2	1.822	3.536	0.001095993	0.007031715
AKAP1	1.818	3.525	5.48E-05	0.000642614
TMEM169	1.813	3.515	0.002650754	0.014156773
C17orf59	1.811	3.51	4.63E-05	0.000561814
PPIP5K1	1.81	3.506	4.49E-05	0.000551043
SCO2	1.808	3.502	1.96E-05	0.000283427
NA	1.807	3.5	0.003644769	0.018048766
ATP5J	1.804	3.491	2.00E-05	0.000287167
BPHL	1.803	3.49	0.000596338	0.004386467
SLC2A6	1.803	3.488	0.001217354	0.007627171
MTMR10	1.801	3.485	0.0001425	0.001419573
PPP1R9A	1.801	3.484	0.002128718	0.011936339
ECH1	1.801	3.483	0.004979545	0.022893557
HOMEZ	1.8	3.483	0.000433544	0.003446069
LACTB2	1.799	3.48	1.38E-06	3.17E-05
MMP24	1.797	3.474	0.00105581	0.006826682
CCDC167	1.796	3.472	3.29E-05	0.000427208
ATP10A	1.795	3.471	9.35E-06	0.000155323
MSRA	1.794	3.468	3.91E-05	0.000493345
NRCAM	1.793	3.466	0.000120113	0.001223424
C4orf32	1.793	3.465	9.09E-06	0.000151723
F11R	1.791	3.46	0.004918085	0.022677532
MGLL	1.788	3.452	0.000104022	0.001092229
SH2B2	1.787	3.452	0.004173192	0.019981986
KLF15	1.785	3.445	0.00113303	0.007218293
PRDX6	1.784	3.443	0.000931091	0.006192709
STXBP2	1.783	3.441	0.009609714	0.038303747
FBXO8	1.783	3.44	1.40E-07	4.46E-06
NA	1.782	3.44	0.004489381	0.021128183
RAVER2	1.782	3.438	3.47E-05	0.000445453
COL4A2	1.779	3.431	0.000160376	0.001564445
PREB	1.778	3.43	5.27E-05	0.00062171
MGST2	1.775	3.423	1.89E-06	4.10E-05
PTRH1	1.774	3.421	0.000128043	0.001293729
SLC5A6	1.772	3.416	0.001889878	0.010888003
PWWP2B	1.772	3.415	6.62E-05	0.000749316
SPTBN5	1.77	3.412	0.004592916	0.021479957
COA6	1.764	3.397	3.35E-05	0.000432353
HIST1H2AI	1.758	3.383	0.000971815	0.006400999
TRMT10C	1.758	3.382	1.96E-05	0.000283427

ADAP1	1.756	3.378	0.003580129	0.017819458
SVIL	1.756	3.377	4.45E-05	0.000547088
C16orf46	1.755	3.375	0.003745218	0.018458864
INSIG1	1.752	3.368	0.0038318	0.018763272
FBXO27	1.751	3.366	0.005779288	0.025818958
UROS	1.751	3.365	6.23E-06	0.000109473
DOCK6	1.747	3.357	0.000383499	0.003128693
MRPL34	1.747	3.356	1.61E-05	0.000243553
MRPL41	1.744	3.35	9.46E-06	0.000156932
IL17RE	1.744	3.349	0.000763796	0.005327388
CMBL	1.741	3.343	4.11E-05	0.000512557
DNAJC30	1.74	3.341	3.08E-06	6.17E-05
NA	1.737	3.334	0.001159417	0.007345131
MPC2	1.737	3.334	0.003456034	0.017392644
MCCC1	1.736	3.33	0.00201933	0.011463933
CECR1	1.736	3.33	0.001352518	0.008338448
TYRO3	1.734	3.327	0.01049025	0.040926412
PTHLH	1.729	3.315	0.002584467	0.013889981
DHRS4L2	1.728	3.314	0.000229573	0.002075765
NFU1	1.728	3.313	0.000172393	0.001656731
GAMT	1.727	3.31	0.000225288	0.002047905
ACO1	1.726	3.309	0.003751464	0.018469414
APLP1	1.726	3.309	0.009687921	0.038547727
SLC2A4RG	1.725	3.305	2.43E-05	0.00033668
EMC6	1.724	3.305	2.23E-06	4.72E-05
MRPS12	1.723	3.302	0.000127322	0.001289131
TMEM160	1.723	3.301	0.000814394	0.005594284
CENPV	1.722	3.299	0.00070688	0.005024518
STK32C	1.721	3.297	0.00073916	0.005195501
HMOX1	1.717	3.287	1.62E-05	0.000244075
ACYP2	1.717	3.287	0.000332623	0.002786282
ANKRD53	1.716	3.285	0.001688039	0.009965652
RAB3A	1.716	3.284	0.003505349	0.017549958
FGFRL1	1.715	3.283	0.000967212	0.006376854
HAVCR2	1.714	3.282	0.004426776	0.02092378
DDX28	1.714	3.281	0.000773617	0.005373658
PITPNA-AS1	1.712	3.275	0.000843495	0.005744886
Mar-02	1.709	3.269	0.012014528	0.045220674
RBMXL1	1.706	3.263	4.87E-06	9.00E-05
NCLN	1.702	3.253	1.94E-05	0.000281336
SEMA4G	1.701	3.252	0.005200644	0.023756416
MRAS	1.701	3.251	0.002539442	0.013691253
ANKDD1A	1.697	3.243	0.000197263	0.001851282
HMBS	1.696	3.241	0.000314337	0.002658481
HACL1	1.695	3.237	1.86E-05	0.00027222
AIG1	1.694	3.235	1.61E-05	0.000243809
COX18	1.693	3.233	3.62E-05	0.000461635
C6orf57	1.693	3.233	0.000315184	0.002663986
NA	1.686	3.217	0.006432318	0.027991998
AKAP12	1.685	3.215	0.002897905	0.015161357

GPR180	1.684	3.213	6.60E-07	1.70E-05
C1orf53	1.684	3.213	0.003104555	0.015966283
FAM173A	1.684	3.212	0.000103129	0.001084531
MPP1	1.682	3.209	0.000542375	0.004066467
SIX1	1.681	3.207	1.56E-06	3.49E-05
DUS2	1.68	3.205	0.000581668	0.004299479
ATP5D	1.68	3.204	3.03E-05	0.000399027
MTURN	1.679	3.201	0.00045474	0.003552297
HSPB6	1.676	3.195	0.00033148	0.002780999
POR	1.675	3.193	0.00474264	0.022019051
HSPA1B	1.674	3.191	2.57E-06	5.30E-05
GPR39	1.673	3.188	0.012256455	0.045959175
IMPAD1	1.672	3.187	0.000181576	0.001727888
ACAA1	1.672	3.187	0.000790924	0.005468949
SETD9	1.672	3.187	0.001682285	0.009935994
APOO	1.672	3.186	0.004956345	0.022816624
SPRY1	1.671	3.184	2.00E-05	0.000287499
MSR1	1.67	3.183	0.002927009	0.015284244
PODXL2	1.67	3.182	0.004720498	0.021923731
RRS1	1.67	3.181	4.62E-05	0.000561682
PPP2R5A	1.667	3.176	0.008794862	0.035746857
NA	1.667	3.174	0.009721223	0.038613185
DHDDS	1.666	3.173	0.00035905	0.002952238
ATP9A	1.665	3.172	0.000309753	0.002634453
CYC1	1.665	3.171	0.007132433	0.030392678
C2orf88	1.664	3.17	0.001408232	0.008608818
ACAD8	1.663	3.168	5.74E-05	0.000665586
HOXA7	1.663	3.167	3.78E-06	7.35E-05
SCAND1	1.663	3.167	1.61E-06	3.56E-05
EI24	1.661	3.163	8.16E-05	0.000887803
ESRRA	1.661	3.163	0.000303605	0.002593504
HSD17B10	1.661	3.163	0.001134517	0.007224385
ZYG11B	1.661	3.162	2.84E-05	0.000381413
COX8A	1.66	3.161	0.00023187	0.002092368
YWHAG	1.659	3.159	0.000551414	0.004120616
SEMA3A	1.658	3.157	0.000340598	0.002829498
FAHD2B	1.658	3.156	0.00279409	0.014748635
KIAA1324L	1.656	3.151	2.61E-06	5.36E-05
ISOC2	1.653	3.145	0.00058711	0.004332645
RNF150	1.65	3.138	1.60E-06	3.55E-05
CAPN5	1.647	3.131	0.000436036	0.003459815
CCDC109B	1.646	3.13	2.46E-05	0.000339908
HADHB	1.645	3.127	0.002257361	0.012492137
CCRN4L	1.645	3.127	0.000130137	0.001313726
EXOSC5	1.644	3.125	0.001612529	0.009622434
ZNF524	1.643	3.122	0.000104937	0.001099292
EXOSC4	1.64	3.118	0.00061558	0.004501243
ADAMTS18	1.64	3.117	0.011721756	0.044419284
ADIPOR2	1.633	3.101	0.003036587	0.015700862
LRIG1	1.631	3.098	0.001336541	0.008252107

PDSS2	1.631	3.098	0.00021018	0.001945663
PTPLA	1.63	3.096	0.002928491	0.015286119
ZNF589	1.629	3.094	0.000252756	0.002237806
CENPI	1.629	3.093	0.001102335	0.007059094
JARID2	1.626	3.086	1.11E-05	0.000179109
NANOS1	1.625	3.084	0.003793368	0.018621991
AKAP3	1.624	3.083	0.011780571	0.044592491
FAM149A	1.624	3.082	0.00623396	0.027338616
NDUFC2	1.624	3.082	1.19E-05	0.000190325
HSD17B1	1.623	3.08	0.006166431	0.027077376
LOC100379224	1.622	3.078	0.000631614	0.004595208
RTN3	1.621	3.077	0.001103826	0.007061995
SAC3D1	1.621	3.076	0.00169039	0.009970885
ITPA	1.621	3.075	9.10E-06	0.000151784
NDUFAB1	1.619	3.072	0.000162712	0.0015793
ATP5G3	1.618	3.068	0.002278848	0.01258035
EIF5A	1.616	3.065	0.00049457	0.003783082
NA	1.615	3.064	0.001404742	0.008595199
OSGIN1	1.615	3.063	0.000151849	0.001497855
CDC20	1.612	3.057	0.011006162	0.042404263
SPRYD4	1.612	3.057	0.000150818	0.0014926
NDUFB6	1.611	3.054	7.70E-06	0.000131328
RTN4IP1	1.61	3.053	0.005216737	0.02381308
GLRX2	1.61	3.052	0.000649719	0.004705363
LNPEP	1.609	3.051	0.000133877	0.001342517
DHODH	1.606	3.044	0.010849658	0.04195571
C8orf82	1.603	3.038	2.68E-05	0.000363551
UVRAG	1.603	3.037	4.43E-05	0.000545647
TRAP1	1.602	3.035	0.003176982	0.016258883
CSAD	1.601	3.034	0.003060827	0.01579512
ANKRD13B	1.601	3.033	2.48E-05	0.000341993
CASP1	1.6	3.032	0.000955867	0.00632261
AMBRA1	1.6	3.03	0.002535613	0.013676031
PAIP2B	1.599	3.03	0.005734656	0.025670131
FDPS	1.599	3.028	0.004121947	0.019799312
SLC25A11	1.598	3.027	0.000577582	0.0042809
ALDH2	1.596	3.023	0.009742084	0.038672778
SLC22A23	1.596	3.022	4.66E-05	0.00056498
PMVK	1.595	3.021	0.000261508	0.002301814
NDUFV3	1.595	3.02	5.07E-06	9.33E-05
SELPLG	1.594	3.019	0.001034262	0.00671481
IPO5P1	1.593	3.018	0.001082485	0.006961463
ADCK3	1.593	3.017	0.003585677	0.017834027
TRIM24	1.593	3.016	7.88E-06	0.00013381
NDUFAF2	1.592	3.014	1.03E-05	0.000167929
ZFP28	1.591	3.013	1.43E-05	0.000222645
GCDH	1.59	3.01	0.002017294	0.011457151
ANKRD9	1.587	3.005	0.003374376	0.017070076
BYSL	1.587	3.005	3.74E-05	0.000475661
TTC38	1.587	3.004	0.001522146	0.009177385

BLVRB	1.585	3.001	0.00654577	0.028376822
TMEM170B	1.584	2.998	0.006232599	0.027338616
EPT1	1.584	2.997	5.06E-06	9.31E-05
RHOD	1.583	2.996	0.000111934	0.001159696
ANO3	1.582	2.995	0.01284817	0.047665728
DHRS4	1.579	2.987	0.000460588	0.003585633
CALCRL	1.577	2.983	0.009030325	0.036475115
RPP40	1.576	2.982	0.000270354	0.002368952
PGRMC1	1.574	2.977	0.000253167	0.002239981
ANKRD33B	1.573	2.976	0.00375051	0.018469414
TFB2M	1.571	2.971	1.46E-05	0.000226234
MRPS18B	1.57	2.968	0.00019723	0.001851282
MTCH2	1.568	2.964	0.002274738	0.012564791
UBTD1	1.567	2.963	1.34E-05	0.000210516
COA7	1.564	2.956	1.44E-05	0.000224089
AP1B1	1.563	2.955	0.000432911	0.003444791
NRN1	1.561	2.951	0.000109072	0.001136491
MPV17L	1.561	2.95	0.011085302	0.042648796
FAM219B	1.56	2.949	0.000774041	0.005373658
MRPS15	1.559	2.947	0.000334842	0.002793707
NDUFAF3	1.559	2.946	4.11E-05	0.000512361
LSS	1.558	2.944	0.00906419	0.036601036
CKS1B	1.557	2.943	0.000820135	0.005616706
TCF21	1.555	2.938	0.005858328	0.026035313
SCML2	1.553	2.934	0.009520425	0.038014653
C19orf60	1.553	2.933	9.73E-05	0.001030059
AK4	1.551	2.931	0.000571655	0.004248155
YRDC	1.551	2.93	0.000472616	0.003658341
FAM222B	1.55	2.928	3.16E-06	6.27E-05
CIB2	1.547	2.922	0.004579926	0.021431788
CD320	1.546	2.921	6.06E-05	0.000696595
CIRH1A	1.546	2.921	0.000105541	0.001104775
SRGAP3	1.545	2.919	0.008265256	0.03410358
SPTBN1	1.544	2.917	0.000434308	0.003450121
PSKH1	1.544	2.916	0.000923377	0.006150422
ANKS1A	1.544	2.916	2.48E-06	5.14E-05
MMACHC	1.544	2.916	0.000226643	0.002056102
PCED1B	1.544	2.915	0.008105001	0.033595145
ICAM3	1.542	2.912	0.011107641	0.042704786
TWF2	1.541	2.91	3.01E-05	0.000396361
PDE8A	1.54	2.907	0.000132835	0.001334038
PSEN2	1.539	2.906	0.000585029	0.004321976
VLDLR	1.539	2.906	0.000177536	0.001700649
TBX3	1.539	2.905	7.29E-06	0.000124975
PNP	1.538	2.904	3.23E-05	0.000421882
E2F5	1.538	2.904	0.005441251	0.024558722
PHOSPHO2	1.537	2.903	0.002413199	0.013125024
EIF4EBP2	1.537	2.901	0.000485781	0.003736862
GRPEL2	1.535	2.897	1.12E-05	0.000180829
MID1IP1	1.534	2.896	0.005053529	0.023162152

ATL2	1.534	2.896	0.000216956	0.001989442
CSTB	1.53	2.888	0.001216806	0.007627171
ELMOD3	1.529	2.886	6.53E-05	0.000741939
ABHD6	1.527	2.882	0.001182892	0.007464872
C15orf39	1.524	2.875	7.73E-06	0.000131725
C1orf115	1.522	2.873	0.007940374	0.033109136
PRKCD	1.521	2.871	0.002746681	0.014549174
SPRY4	1.521	2.87	2.92E-05	0.000388681
TOPORS-AS1	1.521	2.869	0.004978195	0.022893557
FAM107B	1.52	2.867	0.001023902	0.006663443
LRRN4CL	1.519	2.865	0.001217388	0.007627171
UQCRQ	1.517	2.863	0.001399455	0.008566706
NA	1.517	2.861	5.27E-05	0.00062171
STK39	1.516	2.859	0.000421199	0.003365635
BCR	1.515	2.859	2.32E-05	0.000324733
LINC00467	1.512	2.852	0.002384422	0.013004898
LINC01270	1.511	2.851	0.002329849	0.012753252
ANKRD46	1.511	2.85	0.000249213	0.002212193
ABHD10	1.509	2.846	2.73E-05	0.000368744
LENG9	1.508	2.845	0.003085278	0.015891167
SEZ6L2	1.507	2.842	0.003570869	0.017779871
ABHD8	1.506	2.84	0.000220994	0.00201831
TP53INP2	1.505	2.839	0.001193893	0.007518047
ASPH	1.504	2.837	0.001805654	0.010487128
PCCB	1.504	2.837	0.010022736	0.039485062
TIMM13	1.504	2.836	0.000178667	0.001708572
SLC25A42	1.501	2.831	0.001539773	0.009263144
CLPB	1.501	2.831	0.002364588	0.012907064
XPNPEP3	1.499	2.827	0.000202404	0.001885224
PRR5L	1.497	2.823	0.004204895	0.020084315
NDUFB5	1.497	2.823	0.001491327	0.00901554
PIK3R3	1.497	2.823	0.006570641	0.028466502
SLC9A6	1.496	2.82	5.49E-06	9.92E-05
SLC22A5	1.494	2.816	0.000302208	0.002586441
NA	1.493	2.815	0.002765649	0.01460984
GSTZ1	1.491	2.811	0.004110955	0.019760463
UBXN1	1.49	2.81	5.69E-05	0.000661252
C6orf226	1.49	2.809	0.006378065	0.027782558
HEXIM2	1.489	2.807	0.002876862	0.015080252
GPX4	1.488	2.804	0.007096746	0.030283012
BOLA3	1.485	2.8	0.001661776	0.009845491
PRKAR2A-AS1	1.484	2.797	0.006996016	0.029900058
PIEZO1	1.482	2.793	0.001273079	0.007928631
TMEM104	1.48	2.79	0.000139426	0.001392008
ZHX1	1.48	2.79	0.000219561	0.002006569
MCAT	1.479	2.788	0.000410079	0.003294189
NA	1.477	2.784	0.002830874	0.014879312
TRUB2	1.477	2.783	0.000110539	0.001148255
PPP1CC	1.476	2.781	0.000442656	0.003495439
DDT	1.475	2.779	9.71E-05	0.001029685

SNHG9	1.475	2.779	0.009990199	0.039405967
NA	1.474	2.778	0.012797229	0.047515605
THNSL1	1.474	2.777	0.000672537	0.004831457
GCAT	1.473	2.777	0.000344465	0.002851267
NME4	1.473	2.777	0.001124103	0.007168134
BCAP31	1.472	2.774	0.002129735	0.011936339
MVB12A	1.468	2.767	4.13E-05	0.000513985
RHOB	1.468	2.766	0.001999926	0.011401337
UGP2	1.467	2.765	0.00091038	0.006078212
USP6NL	1.467	2.764	2.63E-05	0.00035866
SDHC	1.466	2.764	0.001019713	0.006642533
NME1	1.466	2.763	0.000235489	0.002117568
BTD	1.465	2.761	0.000736053	0.005184373
VWA1	1.465	2.76	0.005697105	0.025544053
C9orf16	1.465	2.76	2.10E-05	0.000299106
TMEM14B	1.463	2.757	5.07E-05	0.000604091
MRPS2	1.463	2.756	0.000581104	0.004299479
KBTBD11	1.463	2.756	0.001311395	0.008122648
HIGD2A	1.461	2.753	5.57E-05	0.000650123
COX17	1.459	2.749	0.000922107	0.006144969
ALG14	1.459	2.749	0.000839915	0.005726236
FADS2	1.458	2.746	0.004021216	0.019459712
POLR2E	1.455	2.742	0.00080062	0.005521964
C12orf66	1.455	2.741	0.001788126	0.010425371
NA	1.455	2.741	0.012712233	0.047290342
PRKAR2A	1.455	2.741	0.001839524	0.010633917
CCNH	1.453	2.738	0.002177977	0.012171627
FDXR	1.452	2.736	0.000822173	0.005622173
MRPL44	1.451	2.734	2.42E-05	0.000335285
MOSPD2	1.45	2.733	9.27E-05	0.00099055
IMPDH1	1.45	2.731	0.002088232	0.011759011
AURKAIP1	1.449	2.731	3.36E-05	0.000433682
QPCTL	1.448	2.729	0.00021285	0.001963701
PISD	1.447	2.727	0.000550285	0.004114439
MAP6D1	1.445	2.723	0.00650754	0.028220078
NOTCH1	1.445	2.722	0.000273255	0.002391288
TP53I13	1.442	2.716	0.000183584	0.001744557
NA	1.441	2.716	3.58E-05	0.000456822
TIMM17B	1.441	2.715	0.000505135	0.003846599
FAM86A	1.439	2.712	0.000284162	0.002470844
CCDC86	1.439	2.711	5.70E-05	0.00066238
BSG	1.438	2.71	0.001549106	0.009298736
FBXO33	1.438	2.709	5.41E-05	0.000634864
DTWD2	1.434	2.702	0.00053844	0.004052596
DHTKD1	1.433	2.699	0.0020341	0.011514155
CEBPB	1.432	2.698	0.000103913	0.001091933
COQ2	1.431	2.697	0.000891874	0.005996353
SPR	1.431	2.697	0.00021881	0.002003746
LGALS3BP	1.431	2.696	0.002589435	0.013905694
XPNPEP1	1.431	2.696	0.000495268	0.003784167

BRI3BP	1.43	2.694	0.001033896	0.00671481
MIR7112	1.429	2.693	0.004768483	0.0221164
ENC1	1.428	2.69	0.004594331	0.021479957
RMND1	1.426	2.688	5.99E-05	0.000689904
VANGL1	1.426	2.686	4.16E-05	0.000517866
PGAM5	1.425	2.685	0.000159268	0.001555866
EXTL3	1.423	2.681	0.000158582	0.001550278
DMGDH	1.421	2.678	0.001417313	0.008648789
PCBD1	1.421	2.677	0.00393721	0.019150323
CHRDL1	1.42	2.677	0.008460048	0.034717832
PPIL1	1.42	2.675	0.000445829	0.003505205
PLCD1	1.42	2.675	0.000269927	0.002366729
ALDH6A1	1.419	2.674	0.002084072	0.011743291
MYOM2	1.419	2.674	0.008034325	0.033394687
NCOA4	1.418	2.672	0.002189195	0.012226263
HSPD1	1.415	2.667	0.002217499	0.01234686
MPND	1.415	2.666	0.001628881	0.009689335
NDUFAF1	1.413	2.663	0.00056711	0.004221679
TBRG4	1.413	2.663	0.000353852	0.002913014
UFSP1	1.413	2.663	0.01308495	0.048359588
GPX1	1.41	2.657	0.003687964	0.018216264
HIST1H4H	1.409	2.656	0.003552024	0.017698991
CHCHD3	1.409	2.656	0.001377488	0.008462691
ASH2L	1.409	2.656	0.000279506	0.002433474
GRAMD1A	1.408	2.654	2.32E-05	0.000324341
ARHGEF37	1.408	2.654	0.006183499	0.027143567
BCL7A	1.406	2.65	0.001456095	0.008841832
EZR	1.405	2.649	0.000289706	0.002503063
PRKX	1.401	2.641	0.000490633	0.003755078
GLRX5	1.4	2.638	0.000404838	0.003261712
COQ3	1.399	2.636	0.004514186	0.021219015
IGSF8	1.398	2.635	0.00562997	0.025309757
C17orf89	1.397	2.634	0.000428249	0.003413949
SARS2	1.397	2.634	0.011620205	0.044169763
PEBP1	1.397	2.633	0.009153499	0.036892755
TBC1D24	1.397	2.633	7.78E-05	0.000854938
YWHAZ	1.396	2.632	0.00104335	0.006767352
LYSMD2	1.396	2.632	0.007630323	0.03205725
TBC1D7	1.396	2.631	0.000747976	0.005243922
ZNF697	1.391	2.622	0.000110092	0.001144486
ABCD1	1.391	2.622	0.000743426	0.005217402
TOB1	1.39	2.621	0.000581641	0.004299479
BRAT1	1.39	2.621	0.000219177	0.002005621
SOX9	1.39	2.621	0.003517591	0.017572459
C9orf72	1.39	2.621	0.004051603	0.019565014
WFS1	1.385	2.612	0.002386984	0.013013651
FPGS	1.38	2.603	0.000853539	0.005784339
C1QBP	1.377	2.598	0.00385671	0.018851334
MADD	1.377	2.597	3.38E-05	0.000435049
COX5B	1.375	2.593	0.002076273	0.011713898

ZSCAN16-AS1	1.375	2.593	0.009544569	0.03809988
BLCAP	1.374	2.592	0.001880147	0.010845716
APOL6	1.374	2.591	7.82E-05	0.000857778
BHLHE41	1.373	2.59	0.000161355	0.001570618
PLA2G12A	1.373	2.59	0.00066991	0.004825909
TIMM8B	1.372	2.588	0.002644425	0.014134065
HACE1	1.372	2.588	5.80E-05	0.000671748
PNPO	1.371	2.587	0.000628	0.004579753
FAM214B	1.368	2.58	0.001699535	0.010009281
NDUFS7	1.365	2.576	0.003116756	0.015998799
GATB	1.365	2.575	0.001490088	0.00901205
DNAH10OS	1.364	2.575	0.004677245	0.021767424
DGKQ	1.364	2.575	0.000115203	0.001185342
LINC00847	1.361	2.569	0.002943142	0.015344932
LAGE3	1.36	2.568	0.001488924	0.009009018
TMEM141	1.358	2.564	0.00065942	0.004765472
MRPL37	1.357	2.562	0.002006368	0.011423704
TBC1D10A	1.357	2.562	0.001788015	0.010425371
SNX16	1.353	2.555	0.000716997	0.005084362
BLVRA	1.353	2.555	0.000162186	0.001575323
PGP	1.352	2.553	0.001056832	0.006828762
NUDT16L1	1.351	2.551	0.002029351	0.011501626
ARSG	1.35	2.55	0.00544071	0.024558722
STRBP	1.35	2.549	0.003180067	0.016262441
LGR4	1.348	2.545	0.004215331	0.020120038
MRPS7	1.347	2.544	0.003076975	0.015860405
UQCC1	1.346	2.542	0.001977061	0.011299391
NA	1.344	2.538	0.012483407	0.046630305
MARK2	1.341	2.533	5.54E-05	0.000647744
MME	1.34	2.531	0.002672373	0.014249864
PTGS2	1.34	2.531	0.006755047	0.029117097
UHMK1	1.335	2.523	0.002252585	0.012475853
MRPL23	1.334	2.521	0.001387086	0.008513968
NOP16	1.334	2.521	0.001148683	0.007294112
MEF2C	1.333	2.52	0.002282477	0.012590172
CACUL1	1.333	2.519	0.000310683	0.002637415
PIK3CA	1.332	2.518	0.000242016	0.002163833
TMTC2	1.332	2.518	0.002033648	0.011514155
NDUFB7	1.331	2.515	0.000529734	0.004004789
CCDC18	1.329	2.512	0.003623692	0.017977104
AK3	1.329	2.511	0.00110456	0.007063367
SCMH1	1.328	2.51	0.000107817	0.001126
GXYLT2	1.327	2.509	0.000376097	0.00307568
RAP1GDS1	1.325	2.506	0.000655098	0.004741795
WBP2	1.325	2.505	0.007578967	0.031890717
MRPS11	1.324	2.503	0.00142678	0.008694858
BANK1	1.324	2.503	0.008625706	0.035259419
STXBP1	1.323	2.502	0.002461798	0.013345073
PSAP	1.322	2.501	0.003205171	0.016366215
TRMT2B	1.321	2.498	0.000645367	0.004678828

SLIRP	1.32	2.497	0.000620681	0.004532927
AVPI1	1.319	2.495	0.003925958	0.019121685
ATP5G1	1.319	2.494	0.001025016	0.006667501
MLF2	1.318	2.493	0.002051027	0.011595501
CDC34	1.317	2.492	0.000191502	0.001804679
CACYBP	1.317	2.491	0.00020047	0.001872336
RPP25L	1.317	2.491	0.011623597	0.044170319
CDK5	1.316	2.49	0.001392963	0.008542332
PSMA7	1.314	2.486	0.008109018	0.033601557
DTD2	1.313	2.485	0.000365938	0.002999806
TMEM245	1.313	2.484	0.003952676	0.019210004
MGMT	1.311	2.482	0.001275907	0.007942612
WDR18	1.311	2.482	0.000216099	0.001986313
COX7A2	1.311	2.481	0.004998209	0.022962737
PLEKHH3	1.311	2.481	0.000958054	0.00632874
NDUFB11	1.311	2.481	0.00161576	0.009622434
NA	1.31	2.48	0.005973888	0.026436642
FURIN	1.31	2.48	0.003473918	0.01746327
NDUFB9	1.31	2.48	0.004919465	0.022677532
GAB3	1.31	2.479	0.00882815	0.035850033
DGKD	1.309	2.477	0.000306761	0.002615542
PPP2R1B	1.308	2.476	0.004281949	0.020373695
NDUFA4	1.308	2.476	0.003257284	0.016588742
ECSIT	1.307	2.475	0.004462266	0.021040372
NATD1	1.307	2.475	0.000292257	0.00251711
MARCKSL1	1.306	2.472	0.011058259	0.042556784
VWA8	1.304	2.469	0.001982943	0.011318743
MLX	1.304	2.469	0.001066103	0.006878868
TTLL12	1.303	2.468	0.000448995	0.003527669
GRB2	1.303	2.467	0.000208598	0.001933894
ARHGAP31	1.303	2.467	0.000723892	0.00512258
IFI6	1.301	2.464	0.001886667	0.010877239
RAB40C	1.301	2.464	0.000450287	0.003533741
TMEM177	1.299	2.461	0.008757594	0.035637959
ATR	1.298	2.458	0.001147913	0.007292626
SH3GLB1	1.298	2.458	0.002091088	0.011768206
FBXL15	1.297	2.458	0.002932073	0.015298945
MRPS16	1.297	2.457	0.000663224	0.004785343
ATG4D	1.296	2.455	0.009731513	0.038642085
ALG8	1.295	2.454	0.000107382	0.00112232
SC5D	1.295	2.454	0.005916785	0.026235128
FAM195B	1.295	2.454	0.002080029	0.011729216
RNMTL1	1.294	2.452	0.004199649	0.020071014
LYPLA1	1.293	2.451	0.000851756	0.005783781
TMLHE	1.293	2.45	0.002341197	0.012794782
HIST1H2BD	1.293	2.45	0.009234741	0.03708066
SLMO2	1.292	2.448	0.000732113	0.005164644
NECAB3	1.292	2.448	0.001896399	0.010904305
ATP5G2	1.292	2.448	0.001770012	0.010350808
IFRD2	1.291	2.447	0.000729144	0.005154388

ACP2	1.29	2.446	0.000977782	0.006434068
FKBP4	1.288	2.442	0.001021868	0.006653385
ANP32A	1.284	2.435	0.000254	0.002244439
CETN2	1.283	2.433	0.002173905	0.012153864
MRPL50	1.282	2.432	0.002462484	0.013345073
CPNE2	1.282	2.431	0.000672491	0.004831457
NDUFS2	1.279	2.427	0.010689325	0.041488758
C21orf33	1.279	2.427	0.011779786	0.044592491
BZW2	1.279	2.426	0.000547514	0.004098649
MRPS18C	1.278	2.425	0.000591719	0.00435956
HOXB2	1.277	2.423	0.001032064	0.006706938
CCDC106	1.276	2.422	0.00053894	0.004054117
C19orf48	1.276	2.421	0.001195011	0.007521603
NDUFB4	1.275	2.42	9.72E-05	0.001029685
ITPK1	1.272	2.414	0.002319452	0.012701451
RPUSD1	1.271	2.413	0.001817395	0.010532842
NADK	1.271	2.413	0.002614716	0.014013807
DUS3L	1.271	2.413	0.00376364	0.01851613
IDH3A	1.268	2.408	0.005455956	0.024600611
DGCR6L	1.268	2.408	0.000816534	0.0056005
TIMM21	1.268	2.408	0.001395463	0.008549957
ATAD3A	1.267	2.407	0.000406261	0.003265443
EHBP1	1.265	2.403	0.002142697	0.011999102
ING1	1.261	2.397	0.002723238	0.014459539
TCAIM	1.261	2.397	0.000439516	0.003483365
CENPL	1.261	2.397	0.002119941	0.011901053
FAM212B	1.258	2.392	0.001717313	0.010094683
CAV1	1.258	2.391	0.006384282	0.027800739
FBXL17	1.254	2.385	0.000167359	0.001615246
SLC50A1	1.253	2.384	0.000316018	0.002669379
GPC1	1.253	2.384	0.00971854	0.038613185
NUBP2	1.252	2.382	0.000415315	0.003326435
NDUFAF4	1.252	2.382	0.004398318	0.020810956
ATP5J2	1.252	2.382	0.000806316	0.005550001
TOM1L2	1.252	2.381	0.00350004	0.017536284
MSI2	1.251	2.381	0.004814385	0.022300776
LIAS	1.251	2.381	0.00999297	0.039405967
ATG10	1.251	2.381	0.003055323	0.015772697
ACOT13	1.251	2.38	0.003374366	0.017070076
UQCRH	1.251	2.38	0.013469235	0.049457462
MRPL19	1.25	2.378	0.000310354	0.002636268
NT5DC1	1.247	2.374	0.000346181	0.002860248
MRPL45	1.247	2.374	0.00078947	0.005461673
NDUFA3	1.247	2.373	0.009985289	0.039405967
MRPL40	1.245	2.371	0.000757099	0.005296967
CYB5B	1.245	2.37	0.003054999	0.015772697
FEM1A	1.244	2.369	0.000514138	0.003902062
PPP1R15B	1.244	2.369	0.000288274	0.002497769
TMEM261	1.244	2.369	0.003202713	0.016359806
SLC25A16	1.244	2.368	0.001705554	0.010034234

NOC4L	1.239	2.361	0.002237846	0.012429639
RRN3	1.239	2.361	0.000461382	0.003589759
GFM1	1.239	2.361	0.008228558	0.033983069
B3GNT2	1.238	2.358	0.000511443	0.003888113
TXNRD2	1.237	2.357	0.006153241	0.027036906
AK9	1.236	2.356	0.005988314	0.026483256
CHKA	1.236	2.356	0.003610245	0.017930007
EFS	1.235	2.353	0.000862393	0.005829828
TLCD2	1.234	2.353	0.009593584	0.038250655
SERGEF	1.234	2.352	0.011965974	0.045118309
ST3GAL4	1.233	2.351	0.00231274	0.01268331
C1orf50	1.233	2.351	0.009803279	0.038881672
PEX14	1.232	2.349	0.004260619	0.020293489
FARSA	1.23	2.345	0.000768321	0.005347982
TARS2	1.229	2.344	0.004654999	0.021701005
ZNF593	1.228	2.342	0.004430275	0.020925784
FLAD1	1.227	2.341	0.002422155	0.013163211
SERPINB6	1.225	2.338	0.004651778	0.021701005
LYRM1	1.223	2.335	0.002802971	0.01477259
VDAC2	1.223	2.335	0.010096179	0.039696273
GPX7	1.222	2.332	0.000740976	0.005202893
WHAMM	1.222	2.332	0.000474149	0.003663952
GADD45GIP1	1.222	2.332	0.000387627	0.003145394
PTGFRN	1.221	2.332	0.003509007	0.017561811
SF3B5	1.221	2.331	0.002473506	0.013389778
TMEM187	1.221	2.331	0.008610817	0.035209134
FXN	1.221	2.33	0.006439675	0.028015056
FECH	1.22	2.329	0.001549957	0.009299743
APEX2	1.219	2.327	0.001462587	0.008873332
MRPL47	1.219	2.327	0.000803529	0.005536415
NDUFB2	1.217	2.325	0.002299271	0.012627579
CDC42EP4	1.217	2.325	0.010410601	0.040697344
MRPL1	1.217	2.324	0.001290964	0.008021663
PPA1	1.215	2.321	0.008117779	0.033627282
ZNF106	1.215	2.321	0.008718827	0.035548952
C19orf70	1.214	2.32	0.002853218	0.014979395
TRPC4AP	1.214	2.32	0.00280049	0.014765235
BTG3	1.213	2.318	0.000852802	0.00578383
TCF7L1	1.213	2.318	0.000736871	0.005187451
ALKBH7	1.213	2.318	0.002816623	0.014827311
STARD4	1.211	2.315	0.005235079	0.023849665
ZNF787	1.21	2.313	0.001240682	0.007751701
TSPAN6	1.209	2.313	0.000954241	0.006315812
TRAF4	1.209	2.312	0.001092446	0.007016918
TOMM40	1.209	2.311	0.013463399	0.049457462
C6orf106	1.208	2.31	0.005770001	0.025785936
STARD7	1.206	2.307	0.012768747	0.047461651
EHD1	1.205	2.305	0.004109428	0.019760463
SLC9A3R1	1.204	2.304	0.007647078	0.032107817
DECR2	1.204	2.303	0.001474742	0.008938283

DCTN4	1.202	2.3	0.004979786	0.022893557
TOMM70A	1.202	2.3	0.002893348	0.015143336
LENG1	1.201	2.299	0.011317083	0.043368873
CEP170B	1.201	2.299	0.00096634	0.006374187
ECI1	1.2	2.297	0.001345166	0.008301598
SCUBE3	1.2	2.297	0.009834806	0.038961293
LARS2	1.2	2.297	0.003135902	0.016084944
FBXO46	1.199	2.296	0.003152944	0.016166261
RSL1D1	1.199	2.296	0.00262742	0.014065275
FAM53B	1.198	2.294	0.004177615	0.019996126
TPST2	1.195	2.29	0.002731706	0.01449242
ABHD11	1.194	2.288	0.011375861	0.043545069
TNFAIP8	1.191	2.284	0.000684038	0.004894002
POLDIP2	1.191	2.283	0.006836471	0.029375024
THAP3	1.189	2.279	0.003923256	0.019121685
TNK2	1.188	2.278	0.001789425	0.010428475
SLC25A39	1.187	2.277	0.003591617	0.017857043
NDUFAF5	1.184	2.272	0.009291119	0.037252077
BAMBI	1.183	2.27	0.006344956	0.027673771
MRPS34	1.181	2.267	0.001832398	0.010597225
RREB1	1.181	2.267	0.001377344	0.008462691
GCLC	1.179	2.265	0.005959029	0.02639491
PEX16	1.177	2.262	0.005819852	0.025932069
MRPL28	1.176	2.26	0.001782207	0.010404237
HIST1H2AC	1.176	2.259	0.002158621	0.012078337
PMEPA1	1.175	2.259	0.000537672	0.004049054
STYX	1.173	2.255	0.000570557	0.004245022
ZNF253	1.173	2.254	0.005623924	0.025290929
ATP6V0E2	1.171	2.251	0.007794627	0.03258657
NKIRAS2	1.17	2.25	0.000996916	0.00652527
PNKD	1.17	2.249	0.013364412	0.049192027
POLRMT	1.169	2.248	0.001237279	0.007733988
MRPS6	1.168	2.247	0.001726701	0.010145486
ATP6V0B	1.167	2.246	0.000417144	0.003339112
NA	1.167	2.245	0.009893043	0.039157803
DCUN1D5	1.163	2.24	0.000714127	0.005066652
ITM2C	1.162	2.238	0.003789239	0.018608434
LONP1	1.161	2.237	0.004884715	0.022563205
TBC1D10B	1.161	2.237	0.001322623	0.008181024
DBT	1.161	2.236	0.002335052	0.012771456
USP30	1.16	2.235	0.000916595	0.006111232
LAMTOR1	1.16	2.234	0.003683977	0.018203181
TSFM	1.159	2.234	0.003924618	0.019121685
HSPA8	1.159	2.234	0.012158242	0.045641204
CHST3	1.159	2.234	0.004049928	0.019565014
MRPS27	1.159	2.233	0.003583214	0.01782829
LYRM7	1.158	2.232	0.001248818	0.007795375
NA	1.158	2.232	0.01058961	0.041184172
UHRF1BP1	1.158	2.231	0.002292476	0.012614646
LSM4	1.158	2.231	0.000414133	0.003318915

MPV17L2	1.158	2.231	0.010395803	0.040662857
ORAI1	1.158	2.231	0.01159693	0.044105931
TOB2	1.157	2.229	0.003178884	0.016262441
SPHK2	1.154	2.225	0.002297875	0.012627579
SCAMP3	1.153	2.225	0.00229117	0.012612559
EIF2B3	1.153	2.224	0.010767693	0.041697997
TIMM44	1.15	2.218	0.000664511	0.004792086
TNIP1	1.149	2.218	0.003200505	0.016354663
RTN4	1.149	2.218	0.01197534	0.04512813
CD97	1.149	2.217	0.000471489	0.003651695
PICALM	1.148	2.216	0.00754441	0.031774787
C21orf2	1.147	2.215	0.008263435	0.03410358
LSM10	1.146	2.213	0.003543517	0.017675759
DMKN	1.145	2.212	0.012346264	0.046232241
CLPTM1	1.143	2.209	0.010573758	0.041157807
SIRT2	1.143	2.209	0.00521928	0.02381308
HN1L	1.143	2.208	0.00051519	0.003907861
DENND4A	1.142	2.207	0.000589617	0.004346431
ATP5O	1.142	2.207	0.008289428	0.034182587
NDUFS8	1.142	2.206	0.005405903	0.02447223
HOXB4	1.141	2.206	0.005134522	0.023494489
ABCB10	1.141	2.205	0.000948442	0.006286606
FOXP4	1.139	2.202	0.006758195	0.029121441
C7orf43	1.137	2.2	0.005841667	0.025990837
SNX8	1.137	2.199	0.001080512	0.006955349
RCOR1	1.137	2.199	0.002054983	0.011613044
PARS2	1.135	2.196	0.01259261	0.046935151
MRPL24	1.135	2.196	0.010984032	0.042330986
TMEM11	1.133	2.194	0.002473685	0.013389778
ISCA1	1.133	2.193	0.00245177	0.013302907
PIGM	1.133	2.193	0.002949392	0.01537141
ANKRD35	1.132	2.192	0.005382819	0.024383955
C2orf47	1.131	2.191	0.007712105	0.032321013
TMEM120B	1.131	2.19	0.0026063	0.013985225
ZBTB7B	1.131	2.189	0.003002415	0.015582328
CNTRL	1.13	2.189	0.004069824	0.019611248
CCDC124	1.129	2.187	0.001199477	0.007536714
RGS2	1.127	2.184	0.011982607	0.04512813
SMDT1	1.127	2.184	0.009443298	0.037773193
LYPLA2	1.127	2.183	0.001397569	0.008559009
RPRD1A	1.126	2.183	0.001328212	0.008211859
MPDU1	1.126	2.182	0.003334639	0.016919379
MRPS18A	1.126	2.182	0.013223618	0.048739707
GRWD1	1.124	2.18	0.001612778	0.009622434
TMEM201	1.124	2.179	0.00416456	0.019947669
COMMD1	1.124	2.179	0.002021275	0.011470187
KIF5B	1.123	2.178	0.007450554	0.031486691
NFS1	1.123	2.177	0.012144876	0.045603606
DUS1L	1.116	2.168	0.00222501	0.012370098
POLE4	1.116	2.167	0.002636275	0.014101583

ZNF436	1.114	2.164	0.002620448	0.014033476
COX10	1.113	2.163	0.008055415	0.03344054
BCAR3	1.111	2.16	0.001406984	0.008605051
RFXANK	1.11	2.159	0.002652434	0.014160188
SLC9A3R2	1.11	2.159	0.003453233	0.017384977
AGRN	1.11	2.158	0.010496499	0.040939053
MYLIP	1.109	2.156	0.007537273	0.031754554
KPNA5	1.108	2.156	0.00684075	0.02938413
CLMP	1.108	2.156	0.008143032	0.033691207
MRPS35	1.108	2.155	0.001299147	0.008054118
RHOT1	1.108	2.155	0.003274428	0.016651129
NMT2	1.107	2.153	0.002957244	0.015394867
PLEKHG3	1.106	2.153	0.0100819	0.039651588
NDUFA2	1.106	2.152	0.011309886	0.043354757
RPAP1	1.105	2.152	0.00244807	0.013293432
POMGNT1	1.105	2.151	0.003805354	0.018674091
GFM2	1.105	2.151	0.010033011	0.039504981
THYN1	1.105	2.15	0.003306765	0.016796737
MRPL4	1.104	2.15	0.007484739	0.031577775
ELOVL1	1.103	2.149	0.008954604	0.036223025
AKIRIN2	1.102	2.147	0.001416706	0.008648789
ELP5	1.102	2.146	0.003073291	0.015848352
PIR	1.102	2.146	0.007298805	0.030980082
PLGRKT	1.1	2.144	0.011445521	0.043713344
C10orf2	1.1	2.144	0.006875961	0.029507435
OGFR	1.098	2.141	0.001613052	0.009622434
RPS19BP1	1.098	2.141	0.002041198	0.011549533
NIT2	1.098	2.14	0.002402824	0.013079054
ICT1	1.096	2.138	0.010550691	0.041079773
DOLPP1	1.096	2.137	0.00401031	0.01941384
C19orf52	1.096	2.137	0.005814331	0.025924447
SNX21	1.095	2.136	0.003383774	0.017104901
VARS	1.094	2.134	0.003517289	0.017572459
RPUSD4	1.094	2.134	0.003092168	0.015920628
COPE	1.093	2.133	0.001787648	0.010425371
DYNLL1	1.092	2.132	0.009507485	0.037974127
AMD1	1.092	2.132	0.007103763	0.030303451
AGK	1.091	2.13	0.002064174	0.011656163
PSMB5	1.09	2.129	0.01068208	0.041485134
PFAS	1.089	2.128	0.00363401	0.018020153
TTI2	1.087	2.124	0.00597951	0.026452915
AIMP2	1.086	2.123	0.001101069	0.007054305
ARID3A	1.086	2.123	0.002711494	0.014424555
MRPL39	1.086	2.122	0.010717404	0.04156239
ARMC6	1.085	2.121	0.004810472	0.022295946
ARHGAP42	1.084	2.12	0.011959594	0.045107027
SCAF1	1.084	2.12	0.0022898	0.012610116
CALM1	1.083	2.118	0.010866198	0.041984967
SLC39A11	1.082	2.118	0.002453904	0.013309179
CYSTM1	1.082	2.117	0.004263029	0.020297863

DESI1	1.081	2.116	0.004828758	0.022357856
APIP	1.081	2.115	0.006329847	0.027625578
MRPL35	1.081	2.115	0.0067085	0.028971521
TAF6L	1.08	2.115	0.011173852	0.042916673
DGKZ	1.077	2.11	0.001444808	0.008781129
PHPT1	1.076	2.108	0.003517087	0.017572459
EEFSEC	1.076	2.108	0.008607551	0.035206358
TIMM50	1.076	2.108	0.005159334	0.0235994
PHYH	1.075	2.106	0.006123497	0.026932304
NPC1	1.074	2.106	0.009297388	0.037266231
RAD23A	1.074	2.105	0.006632691	0.02868054
C14orf2	1.073	2.104	0.011112408	0.042704786
C11orf95	1.072	2.102	0.007321487	0.031049317
ARL4A	1.072	2.102	0.008083267	0.03352548
STRA13	1.072	2.102	0.00988052	0.039127234
NLE1	1.071	2.101	0.005450352	0.02458349
SNHG16	1.07	2.099	0.010869976	0.041986553
FAHD1	1.069	2.098	0.006252265	0.027374781
ITPKC	1.068	2.096	0.004848379	0.022433436
SDHD	1.068	2.096	0.009466503	0.03783339
TFE3	1.065	2.093	0.009280021	0.037218546
GFPT1	1.065	2.092	0.010516343	0.040992951
RACGAP1	1.064	2.09	0.003009388	0.015612564
PIN1	1.063	2.09	0.002497705	0.013498321
LMO4	1.062	2.088	0.00770419	0.032297787
NANP	1.055	2.078	0.006730001	0.029038727
MLST8	1.054	2.076	0.008381403	0.034488701
GLCCI1	1.054	2.076	0.005862587	0.026045735
QKI	1.054	2.076	0.012563789	0.046866237
URM1	1.053	2.075	0.00239759	0.013055781
TOMM22	1.053	2.074	0.00414386	0.019876507
PEX3	1.053	2.074	0.003671177	0.018153117
WSB2	1.051	2.072	0.010024706	0.039485062
MAP3K11	1.051	2.072	0.0058445	0.025990837
HFE	1.051	2.071	0.001941742	0.011120885
BANF1	1.05	2.071	0.001439026	0.008753808
BAG5	1.05	2.07	0.005343161	0.024236579
KLC2	1.05	2.07	0.003109472	0.015985531
TSR3	1.049	2.07	0.002451451	0.013302907
MED8	1.048	2.068	0.00226264	0.012506097
JAGN1	1.048	2.067	0.008389709	0.034512443
ACADSB	1.047	2.066	0.00827811	0.034146261
FKBP1A	1.047	2.066	0.011689352	0.044321175
PPP1R14B	1.047	2.066	0.003132426	0.016073175
NUP88	1.047	2.066	0.004374855	0.020728768
CDKN2AIPNL	1.046	2.065	0.006890936	0.029553058
ADRM1	1.045	2.064	0.00570205	0.025557806
MALSU1	1.045	2.063	0.011340542	0.043422085
ZNF672	1.044	2.062	0.004519269	0.02122824
HAUS2	1.044	2.062	0.004633972	0.021634081

GGCT	1.044	2.061	0.008897739	0.036057304
B3GAT3	1.043	2.061	0.006377818	0.027782558
SLC25A25	1.043	2.06	0.004448258	0.020996147
MFSD3	1.043	2.06	0.010849427	0.04195571
ULK3	1.042	2.059	0.004081123	0.019658733
DERL1	1.041	2.057	0.011036871	0.042510542
CRLS1	1.041	2.057	0.010834504	0.041920934
USP13	1.039	2.054	0.004652131	0.021701005
SDC2	1.038	2.053	0.012918753	0.047862344
COA4	1.038	2.053	0.010827857	0.041907131
ERAL1	1.037	2.052	0.007700108	0.032290624
STUB1	1.036	2.051	0.003073472	0.015848352
HSPA4L	1.036	2.051	0.011109284	0.042704786
EARS2	1.036	2.051	0.008575519	0.035117563
GTDC1	1.035	2.049	0.003081969	0.015880133
MAP3K1	1.033	2.046	0.013454247	0.049442449
ATP6V1D	1.033	2.046	0.003168484	0.016227601
URB2	1.032	2.045	0.010524979	0.041014867
TRAF3	1.031	2.044	0.002688532	0.014324799
COMMD9	1.031	2.043	0.00301095	0.015614711
WBSCR16	1.031	2.043	0.003997519	0.019379493
TBX15	1.03	2.042	0.002544073	0.01371079
DNAJA3	1.029	2.041	0.00683189	0.029364609
SCCPDH	1.028	2.04	0.006780766	0.029181739
LRFN4	1.028	2.039	0.007133614	0.030392678
GTF3C6	1.027	2.037	0.002297341	0.012627579
TMEM132A	1.026	2.037	0.0113358	0.043416147
PIAS4	1.026	2.036	0.008366812	0.034458201
INF2	1.026	2.036	0.008721797	0.035548952
SGTA	1.026	2.036	0.001994885	0.011377364
PRDX5	1.024	2.034	0.01209182	0.045429455
PDE12	1.023	2.032	0.004855092	0.022449231
CAP2	1.023	2.032	0.011057922	0.042556784
PAICS	1.022	2.03	0.012337037	0.046210405
UBE2E2	1.02	2.029	0.00220322	0.01228243
TBCA	1.02	2.027	0.003607177	0.017921311
CINP	1.019	2.027	0.003472316	0.017461669
HSBP1L1	1.018	2.025	0.007934364	0.033099578
HSPB8	1.017	2.024	0.012908939	0.047852042
GUF1	1.016	2.023	0.00385587	0.018851334
PTPN1	1.016	2.023	0.00365821	0.018102152
NOC2L	1.016	2.022	0.004923357	0.022687788
MRS2	1.016	2.022	0.009624441	0.038351212
GM2A	1.012	2.017	0.006730463	0.029038727
PLEKHO2	1.012	2.017	0.005528622	0.024903505
SLC36A4	1.012	2.016	0.00282726	0.014871805
INPP5K	1.01	2.014	0.003971574	0.019281189
NHP2	1.009	2.013	0.003784734	0.018593016
KLHL21	1.007	2.01	0.012481556	0.046630305
SYPL2	1.007	2.009	0.010673967	0.041476683

TSSC1	1.006	2.008	0.008743542	0.035612725
TRAF3IP2	1.005	2.006	0.004218054	0.020125975
HYAL2	1.003	2.005	0.006554688	0.028406432
MRPS10	1.003	2.004	0.007130698	0.030392678
LRP3	1	2	0.003238886	0.016507402
NOL6	1	2	0.004147393	0.019886442
NDUFS6	1	2	0.008081544	0.03352548
FARSB	1	2	0.006969834	0.029806885
MICALL1	0.999	1.998	0.009649908	0.038441435
CCNC	0.999	1.998	0.004200181	0.020071014
LYRM5	0.999	1.998	0.008171817	0.033789754
HEATR5B	0.997	1.996	0.006588933	0.028527584
C2orf43	0.995	1.993	0.006078506	0.026795046
POP7	0.994	1.992	0.010073867	0.039631448
GJC1	0.993	1.991	0.008578255	0.035118198
SOWAHC	0.993	1.99	0.005306961	0.024091888
RERE	0.993	1.99	0.01340463	0.049326719
MIER2	0.992	1.989	0.006464856	0.028108168
UBE2A	0.99	1.986	0.010277218	0.040280064
SIL1	0.989	1.985	0.007745548	0.032421229
DDX49	0.984	1.978	0.006788792	0.029207043
FEM1C	0.981	1.973	0.0034056	0.01717695
SLC52A2	0.98	1.973	0.003889619	0.018998541
GDF11	0.98	1.972	0.006736244	0.029054457
OLA1	0.979	1.971	0.008293387	0.034188552
RNPEPL1	0.979	1.97	0.013135333	0.048519438
TTC7B	0.978	1.97	0.004674978	0.021764316
MRPL57	0.977	1.968	0.008368927	0.034458201
ATP6V1G1	0.973	1.964	0.008139305	0.033691207
MRPL15	0.973	1.962	0.013142748	0.048523497
SCO1	0.971	1.96	0.0086993	0.035505398
DPH2	0.966	1.953	0.013423267	0.049381947
VPS25	0.964	1.951	0.008520082	0.03492207
TMEM218	0.963	1.95	0.009721397	0.038613185
PTDSS2	0.963	1.949	0.00594472	0.026350407
COPS3	0.961	1.946	0.007257637	0.030824571
SSNA1	0.958	1.943	0.007206187	0.030653888
PTPRK	0.958	1.943	0.013633172	0.049924705
COASY	0.957	1.941	0.00859333	0.035159302
TBC1D4	0.956	1.94	0.011860759	0.044880467
PSMB6	0.955	1.938	0.012996356	0.048110558
ZNF354A	0.953	1.936	0.009158122	0.036892755
TMCC1	0.953	1.936	0.007941521	0.033109136
TOP1	0.952	1.934	0.010942815	0.042219593
BCAT2	0.951	1.934	0.007425323	0.03140092
CSK	0.951	1.933	0.00501502	0.023024425
GLRX3	0.95	1.932	0.004067562	0.01960729
RPTOR	0.95	1.932	0.006372898	0.027777834
MON1A	0.948	1.929	0.010179647	0.039955187
PHF23	0.947	1.927	0.007664633	0.032171599

NSMCE4A	0.946	1.927	0.013468802	0.049457462
NLRX1	0.945	1.926	0.004219545	0.020126031
MRPL51	0.94	1.919	0.010612999	0.04126334
TXNL4A	0.937	1.915	0.005766346	0.025785936
LBR	0.936	1.914	0.008434528	0.034665375
PPA2	0.935	1.912	0.011985087	0.04512813
NBPF3	0.935	1.912	0.008379848	0.034488701
C9orf91	0.932	1.907	0.005237004	0.023850453
CCM2	0.931	1.907	0.009405484	0.037644068
TPMT	0.931	1.907	0.009708258	0.038606071
SNTB1	0.931	1.906	0.007998335	0.033305185
GTF3A	0.929	1.904	0.012263324	0.045972261
MBD3	0.929	1.903	0.005436112	0.024551813
NCALD	0.928	1.903	0.011620092	0.044169763
TMEM189	0.926	1.9	0.006221556	0.027301819
NAA25	0.926	1.899	0.005636525	0.025330855
CEBPZOS	0.925	1.899	0.007536468	0.031754554
LRRC47	0.925	1.899	0.006882214	0.029524956
HOOK3	0.924	1.898	0.00970032	0.038585781
SIRT1	0.924	1.898	0.005276959	0.023976248
TRPS1	0.921	1.894	0.00761757	0.032023445
NLN	0.92	1.892	0.007307877	0.031008916
DAZAP1	0.919	1.891	0.008701536	0.035505398
MRPL42	0.918	1.89	0.010787432	0.041762555
BAG4	0.918	1.889	0.005841899	0.025990837
UBQLN4	0.918	1.889	0.006490663	0.028173826
HLTF	0.917	1.888	0.00880656	0.035783715
CXorf56	0.917	1.888	0.010211632	0.040069173
EBNA1BP2	0.917	1.888	0.008050643	0.03343093
PSMD10	0.915	1.886	0.012293872	0.046074088
PNO1	0.915	1.885	0.007686942	0.032255292
FLOT2	0.913	1.883	0.008495602	0.034842723
AMMECR1L	0.912	1.881	0.012423905	0.046459053
CLPP	0.909	1.878	0.012733927	0.047345157
HEATR2	0.907	1.875	0.010682239	0.041485134
C6orf203	0.906	1.874	0.012848019	0.047665728
MRPS30	0.906	1.873	0.007330138	0.031064629
TMEM69	0.903	1.87	0.00778216	0.032544447
PTPN4	0.9	1.866	0.01094582	0.042219593
NOP2	0.9	1.866	0.013202045	0.048686567
SNX11	0.9	1.866	0.011451252	0.043722962
TMEM161A	0.9	1.866	0.010392305	0.040660863
FBXO22	0.899	1.865	0.008593462	0.035159302
MMGT1	0.899	1.865	0.010321141	0.04041729
APOL2	0.898	1.864	0.01241987	0.046456731
ERI3	0.893	1.857	0.008854189	0.03593433
NPRL3	0.891	1.855	0.012692405	0.04724241
DUSP1	0.891	1.854	0.009371552	0.037530335
TP53I3	0.891	1.854	0.009466688	0.03783339
CSRNP2	0.889	1.851	0.00878886	0.035733135

DKC1	0.888	1.851	0.009579923	0.038218583
FASTK	0.887	1.85	0.011910919	0.044998276
RRP36	0.887	1.849	0.01207985	0.045397018
CES2	0.886	1.847	0.01305136	0.048274778
REPIN1	0.885	1.846	0.00872266	0.035548952
H3F3A	0.884	1.846	0.010236675	0.040144285
RRP1B	0.884	1.845	0.007497028	0.031614366
RNF126	0.881	1.842	0.011320525	0.04336985
GTF3C3	0.88	1.841	0.010451065	0.040804928
CDK8	0.877	1.837	0.012386268	0.046343781
DCUN1D3	0.876	1.836	0.010445899	0.040800171
ZNF189	0.873	1.831	0.010759125	0.041676681
INTS4	0.872	1.83	0.012774053	0.047468408
UTP11L	0.87	1.828	0.011107756	0.042704786
MINA	0.87	1.828	0.008742587	0.035612725
SMYD5	0.866	1.823	0.012432619	0.046478867
RRP12	0.864	1.82	0.012913339	0.047855315
MIR4656	0.853	1.807	0.011395921	0.043597326
NEU1	0.853	1.806	0.010706318	0.041531234
HIST1H2BK	0.848	1.8	0.013301728	0.048987797
C12orf4	0.816	1.761	0.011958719	0.045107027

Gene symbol	Log FC (SGBS vs PHWSC)	Fold Change (SGBS vs PHWSC)	p value	FDR
PDZRN4	-10.792	-1772.564	3.19E-10	2.46E-08
STEAP4	-10.709	-1674.061	1.52E-07	4.79E-06
ISM1	-10.55	-1499.044	5.59E-17	1.90E-14
NA	-10.493	-1440.67	5.05E-18	2.37E-15
SMOC2	-10.139	-1127.336	8.12E-18	3.45E-15
PRKG2	-9.91	-962.18	6.70E-10	4.68E-08
ENPP5	-9.758	-866.033	2.56E-10	2.06E-08
LOC100129940	-9.719	-843.005	2.44E-10	1.98E-08
ELMO1	-9.416	-683.302	5.29E-05	0.000624
NA	-9.401	-676.192	1.82E-10	1.53E-08
CP	-9.287	-624.593	2.57E-10	2.06E-08
CCDC3	-9.2	-588.276	2.33E-07	6.87E-06
NA	-9.101	-548.955	5.92E-11	5.63E-09
TMEM176B	-9.088	-544.185	1.91E-06	4.12E-05
PROK1	-8.982	-505.609	8.07E-05	0.00088
CACNA1G	-8.913	-481.987	1.22E-09	7.98E-08
XIST	-8.901	-478.15	2.19E-06	4.66E-05
PPFIA4	-8.747	-429.784	1.79E-08	7.57E-07
NA	-8.726	-423.345	2.45E-10	1.98E-08
CNTNAP4	-8.692	-413.468	2.27E-06	4.79E-05
SFRP2	-8.589	-385.029	1.34E-17	4.93E-15
IL7R	-8.53	-369.617	4.34E-09	2.34E-07
PLXDC2	-8.508	-363.988	1.23E-17	4.75E-15
TMEM176A	-8.455	-350.899	1.61E-05	0.000244
CYP19A1	-8.416	-341.578	6.50E-10	4.59E-08
A2M	-8.411	-340.324	2.49E-20	2.42E-17
SLC14A2	-8.394	-336.308	4.62E-08	1.73E-06
SERPINB7	-8.309	-317.238	1.38E-08	6.17E-07
PDPN	-8.273	-309.375	3.10E-09	1.76E-07
LRRC15	-8.27	-308.597	1.53E-05	0.000235
COL4A4	-8.2	-294.106	5.13E-08	1.91E-06
CNTN1	-8.141	-282.226	1.44E-06	3.27E-05
CNGA3	-8.123	-278.854	0.003351	0.016977
SALL1	-8.123	-278.825	9.01E-06	0.000151
COL6A6	-8.107	-275.693	0.000166	0.001607
FAIM2	-8.045	-264.181	1.14E-05	0.000183
IL32	-8.024	-260.259	6.65E-06	0.000116
GABRA5	-8.021	-259.711	2.12E-08	8.78E-07
HTR2B	-8.008	-257.419	2.00E-08	8.38E-07
NA	-7.956	-248.359	1.90E-07	5.79E-06
LOC101929579	-7.927	-243.358	2.17E-05	0.000308
SERPINA5	-7.923	-242.721	2.87E-05	0.000385
BDKRB1	-7.862	-232.634	1.70E-05	0.000252
MKI67	-7.844	-229.736	6.77E-11	6.22E-09
ESM1	-7.838	-228.871	0.000122	0.001236
NTNG1	-7.811	-224.559	9.29E-13	1.33E-10

SYNDIG1	-7.717	-210.394	1.04E-07	3.47E-06
NA	-7.685	-205.798	1.35E-06	3.13E-05
ZFP57	-7.662	-202.495	1.89E-07	5.79E-06
CCNE2	-7.656	-201.709	9.56E-06	0.000158
COL4A3	-7.576	-190.801	0.000223	0.002033
GALNT13	-7.568	-189.704	1.86E-10	1.55E-08
NGFR	-7.466	-176.774	0.000118	0.001204
FAM155A	-7.461	-176.174	1.22E-05	0.000194
COMP	-7.456	-175.608	4.75E-26	8.08E-23
WISP2	-7.42	-171.269	3.21E-25	4.86E-22
NA	-7.417	-170.874	0.007229	0.030732
CYP39A1	-7.413	-170.454	1.25E-06	2.93E-05
MMP3	-7.397	-168.597	3.99E-15	9.36E-13
NA	-7.396	-168.476	1.13E-06	2.67E-05
RTP4	-7.328	-160.67	2.14E-06	4.56E-05
PRRG4	-7.317	-159.509	3.82E-06	7.41E-05
GPR56	-7.317	-159.421	0.000873	0.005885
THBD	-7.306	-158.235	5.38E-05	0.000633
ALDH1A1	-7.229	-150.049	3.66E-14	7.33E-12
WNT2	-7.211	-148.186	4.45E-05	0.000547
BRINP1	-7.209	-147.947	3.51E-09	1.96E-07
COL10A1	-7.204	-147.402	1.47E-05	0.000226
FGF9	-7.199	-146.913	0.000119	0.001217
STMN2	-7.182	-145.203	2.37E-19	1.79E-16
GPSM3	-7.166	-143.628	5.48E-06	9.92E-05
LOC101929484	-7.156	-142.587	4.76E-06	8.87E-05
BDKRB2	-7.14	-141.008	1.56E-08	6.78E-07
AMZ1	-7.129	-139.979	0.000544	0.004075
TACSTD2	-7.1	-137.222	2.88E-06	5.84E-05
DAB1	-7.1	-137.218	1.74E-15	4.47E-13
TRH	-7.1	-137.156	7.21E-05	0.000804
ANKFN1	-7.094	-136.664	8.70E-05	0.000937
PRCD	-7.065	-133.885	2.65E-05	0.000361
HTRA4	-7.033	-130.931	3.82E-05	0.000484
ALX1	-7.011	-129.023	4.55E-05	0.000554
GPR133	-7.003	-128.231	6.54E-10	4.59E-08
COL11A1	-6.983	-126.541	2.48E-09	1.46E-07
KRTAP2-3	-6.977	-125.969	0.000113	0.001171
HS3ST2	-6.954	-124.015	0.002201	0.012279
IFITM1	-6.946	-123.321	1.34E-13	2.37E-11
DKK2	-6.935	-122.34	3.20E-06	6.35E-05
RORB	-6.929	-121.845	1.78E-06	3.88E-05
PTPRE	-6.923	-121.354	0.00018	0.001718
CHL1	-6.884	-118.095	3.84E-09	2.11E-07
LDLRAD4	-6.859	-116.105	7.56E-11	6.86E-09
USP32P2	-6.85	-115.392	1.23E-09	8.04E-08
NOVA1	-6.786	-110.35	1.50E-11	1.64E-09
SCN2A	-6.783	-110.146	0.0003	0.002567
POM121L9P	-6.769	-109.032	1.89E-18	1.03E-15
TLR4	-6.748	-107.503	9.70E-15	2.09E-12

FLG	-6.734	-106.438	0.003549	0.01769
RAD54L	-6.726	-105.883	0.000276	0.002408
DOCK2	-6.718	-105.26	0.000149	0.001481
LINC01305	-6.634	-99.338	1.39E-05	0.000217
CD163	-6.505	-90.805	7.63E-10	5.16E-08
WBSCR17	-6.497	-90.3	4.61E-06	8.65E-05
IGSF3	-6.476	-89.039	2.20E-05	0.000311
CLEC2B	-6.466	-88.38	6.32E-21	6.61E-18
CNR1	-6.461	-88.118	5.40E-05	0.000634
HLA-F	-6.353	-81.713	1.40E-08	6.20E-07
SERPINB2	-6.348	-81.452	4.37E-05	0.000541
PTGIR	-6.304	-79.007	8.16E-12	9.41E-10
SAA2	-6.276	-77.486	0.000213	0.001964
PIEZO2	-6.204	-73.733	8.49E-06	0.000143
AQP1	-6.152	-71.11	1.96E-13	3.26E-11
NA	-6.142	-70.626	1.30E-08	5.87E-07
MMP1	-6.139	-70.455	0.000296	0.002545
NR4A1	-6.132	-70.113	2.84E-07	8.11E-06
PRL	-6.126	-69.822	2.82E-08	1.12E-06
ZNF521	-6.099	-68.544	2.65E-19	1.90E-16
ADCY4	-6.076	-67.444	6.21E-05	0.000711
SUSD2	-6.067	-67.043	8.52E-27	1.93E-23
SEPP1	-6.035	-65.579	1.83E-13	3.16E-11
MTUS1	-5.952	-61.909	5.42E-12	6.52E-10
NA	-5.922	-60.639	0.000289	0.002501
TNXB	-5.879	-58.841	7.21E-06	0.000124
COL13A1	-5.855	-57.868	0.000171	0.001646
CLIC3	-5.809	-56.07	1.76E-09	1.11E-07
EVI2A	-5.806	-55.954	2.30E-07	6.81E-06
RRM2	-5.798	-55.622	2.75E-08	1.09E-06
SAMD11	-5.797	-55.618	6.02E-07	1.58E-05
HECW1	-5.757	-54.096	3.77E-09	2.09E-07
NA	-5.742	-53.524	4.12E-13	6.23E-11
GIPC2	-5.714	-52.502	3.75E-07	1.03E-05
CHI3L1	-5.697	-51.86	1.26E-17	4.75E-15
DNASE1L3	-5.692	-51.71	4.08E-05	0.00051
NPNT	-5.684	-51.413	0.001186	0.007472
PLA2G5	-5.67	-50.901	1.11E-07	3.67E-06
GREM1	-5.667	-50.807	2.40E-08	9.78E-07
SORCS2	-5.644	-50.005	2.29E-16	6.63E-14
LOC101928188	-5.633	-49.641	4.41E-13	6.60E-11
MEGF6	-5.619	-49.132	3.72E-07	1.03E-05
EGFL6	-5.603	-48.612	0.001267	0.007898
CEMIP	-5.57	-47.509	1.04E-06	2.49E-05
GRID1	-5.568	-47.454	2.96E-07	8.37E-06
IL1RL1	-5.562	-47.229	1.89E-07	5.79E-06
IL20RB	-5.548	-46.784	5.81E-09	2.94E-07
LINC01436	-5.547	-46.756	0.000108	0.001129
GABBR2	-5.536	-46.398	4.10E-06	7.86E-05
MXRA5	-5.52	-45.891	1.99E-24	2.71E-21

IL13RA2	-5.509	-45.541	7.18E-07	1.83E-05
KCNJ15	-5.448	-43.639	7.99E-07	1.99E-05
NA	-5.443	-43.511	3.68E-06	7.16E-05
SP9	-5.43	-43.11	3.13E-05	0.000409
HMCN1	-5.427	-43.011	3.62E-11	3.60E-09
NA	-5.425	-42.958	3.56E-06	6.94E-05
CNKSR2	-5.422	-42.869	8.32E-08	2.92E-06
PTPRQ	-5.421	-42.833	7.68E-11	6.93E-09
LOC101927943	-5.419	-42.773	2.33E-08	9.56E-07
RAB27B	-5.411	-42.534	0.000709	0.005035
RNF144A	-5.406	-42.399	9.16E-09	4.33E-07
GUCY1B3	-5.404	-42.336	4.92E-09	2.56E-07
FAM111B	-5.387	-41.848	1.66E-05	0.000249
SLC25A27	-5.381	-41.679	2.34E-12	3.01E-10
RARRES2	-5.359	-41.038	2.81E-09	1.62E-07
CYP7B1	-5.359	-41.027	1.02E-07	3.43E-06
RAPGEF5	-5.357	-40.97	7.60E-05	0.000839
GPR68	-5.346	-40.687	0.000864	0.005834
CNTNAP3B	-5.341	-40.532	2.07E-07	6.24E-06
IGJ	-5.319	-39.92	1.51E-05	0.000233
FAM180A	-5.318	-39.886	2.32E-14	4.79E-12
DMRT2	-5.297	-39.31	5.69E-05	0.000662
DIRAS2	-5.295	-39.265	0.011982	0.045128
SRRM3	-5.294	-39.224	6.69E-11	6.20E-09
FNDC1	-5.279	-38.827	1.64E-07	5.11E-06
TRPC6	-5.257	-38.252	0.000115	0.001185
FAM153B	-5.256	-38.224	0.000335	0.002794
CMKLR1	-5.255	-38.198	7.39E-09	3.59E-07
MYH1	-5.237	-37.706	0.000737	0.005188
FRAS1	-5.198	-36.719	1.39E-08	6.18E-07
EMP1	-5.187	-36.42	1.37E-18	8.08E-16
ANKRD29	-5.17	-35.999	4.77E-05	0.000575
RAI2	-5.149	-35.478	6.00E-09	2.98E-07
JAM2	-5.128	-34.971	2.08E-09	1.27E-07
C7	-5.119	-34.752	1.21E-06	2.84E-05
PSG5	-5.116	-34.682	1.78E-08	7.54E-07
SEC14L5	-5.112	-34.58	0.000131	0.001318
MUSK	-5.108	-34.481	5.26E-06	9.60E-05
MKX	-5.099	-34.277	2.71E-05	0.000367
FAM225B	-5.095	-34.171	1.99E-06	4.28E-05
GDF6	-5.094	-34.165	2.89E-08	1.14E-06
PTGIS	-5.091	-34.073	3.24E-11	3.29E-09
OMG	-5.09	-34.07	2.32E-09	1.39E-07
GREM2	-5.064	-33.449	8.59E-12	9.82E-10
RFX8	-5.017	-32.382	0.000296	0.002543
ERAP2	-5.004	-32.1	1.24E-21	1.40E-18
TNFAIP6	-4.975	-31.45	1.15E-05	0.000184
ITGA10	-4.969	-31.319	5.41E-07	1.44E-05
KCNT2	-4.96	-31.13	3.61E-07	1.00E-05
SKA3	-4.96	-31.115	1.07E-05	0.000175

KRTAP1-5	-4.959	-31.098	3.10E-06	6.20E-05
FMN1	-4.952	-30.955	2.20E-06	4.66E-05
RXFP1	-4.924	-30.362	0.000239	0.002142
SETBP1	-4.92	-30.279	7.80E-18	3.45E-15
SAA1	-4.918	-30.232	1.63E-06	3.61E-05
FLG-AS1	-4.914	-30.138	2.27E-08	9.35E-07
EYA4	-4.899	-29.837	4.75E-08	1.78E-06
FMO1	-4.897	-29.79	0.001673	0.009896
PAMR1	-4.889	-29.637	9.71E-08	3.32E-06
NA	-4.884	-29.534	2.00E-05	0.000287
NOV	-4.884	-29.53	1.06E-07	3.53E-06
LINC00954	-4.883	-29.498	3.88E-05	0.000491
STC1	-4.87	-29.252	0.002698	0.014364
ITPR3	-4.855	-28.94	1.43E-10	1.23E-08
EYA1	-4.839	-28.623	1.11E-05	0.000179
RASL11B	-4.818	-28.205	7.66E-06	0.000131
IFI44L	-4.806	-27.97	7.08E-10	4.89E-08
IL17RD	-4.805	-27.949	0.000225	0.002046
ARSI	-4.794	-27.752	0.000264	0.002322
NA	-4.79	-27.669	0.000209	0.001938
NA	-4.754	-26.989	0.00073	0.005158
MCM10	-4.751	-26.922	0.000441	0.003487
UNC5C	-4.743	-26.773	7.77E-08	2.75E-06
OLFML1	-4.727	-26.489	9.56E-08	3.28E-06
MASP1	-4.726	-26.459	4.48E-18	2.18E-15
FAM84A	-4.726	-26.456	0.000488	0.00374
PEG3	-4.725	-26.455	3.41E-09	1.91E-07
TFPI2	-4.71	-26.166	2.02E-09	1.24E-07
PLCB4	-4.694	-25.889	9.24E-07	2.25E-05
GALNT15	-4.691	-25.825	1.26E-17	4.75E-15
TM4SF1	-4.676	-25.563	2.52E-06	5.21E-05
DKK1	-4.672	-25.489	2.91E-15	7.34E-13
RIPK3	-4.658	-25.255	3.16E-05	0.000413
C10orf10	-4.657	-25.223	6.26E-07	1.63E-05
LINC01085	-4.646	-25.039	3.87E-05	0.000489
KCNS3	-4.637	-24.89	6.08E-07	1.59E-05
CACNB2	-4.637	-24.878	1.68E-05	0.00025
PPP2R2C	-4.627	-24.706	0.00063	0.004587
TNFSF10	-4.623	-24.636	1.99E-13	3.26E-11
GAS7	-4.621	-24.601	1.48E-12	2.02E-10
FLJ12825	-4.614	-24.496	0.000943	0.006259
TINAGL1	-4.602	-24.286	3.50E-11	3.53E-09
CDC45	-4.6	-24.258	0.000642	0.004661
P4HA3	-4.599	-24.229	2.89E-09	1.65E-07
TMEM150C	-4.597	-24.196	0.000629	0.004582
CXCL8	-4.59	-24.082	9.05E-05	0.000969
WISP1	-4.586	-24.011	3.93E-08	1.50E-06
CHN2	-4.583	-23.966	8.08E-05	0.00088
LBP	-4.57	-23.755	0.000226	0.00205
DIRAS1	-4.566	-23.694	1.29E-08	5.86E-07

CXCL5	-4.566	-23.688	5.89E-06	0.000105
FAM225A	-4.563	-23.645	3.15E-06	6.26E-05
ROBO4	-4.563	-23.631	0.001073	0.006917
VIT	-4.559	-23.579	3.77E-13	5.76E-11
SCARA3	-4.549	-23.405	3.47E-13	5.37E-11
ADH4	-4.548	-23.394	3.08E-05	0.000404
PARM1	-4.535	-23.187	1.18E-07	3.88E-06
FER1L4	-4.527	-23.048	1.09E-05	0.000177
FGF7	-4.518	-22.91	9.69E-14	1.83E-11
MIR214	-4.515	-22.868	4.84E-09	2.55E-07
GSC	-4.515	-22.858	0.000179	0.001709
TMEM30B	-4.507	-22.743	4.24E-09	2.29E-07
ZNF208	-4.502	-22.659	6.75E-05	0.000762
PRRT2	-4.496	-22.567	0.000131	0.001318
GDF10	-4.49	-22.468	0.002167	0.01212
FAM167A	-4.488	-22.434	1.68E-07	5.21E-06
SLC2A5	-4.482	-22.35	3.93E-05	0.000495
AVPR1A	-4.468	-22.137	0.000949	0.006287
MCTP2	-4.46	-22.005	5.80E-06	0.000104
SERTAD4	-4.458	-21.979	0.000218	0.001995
MAP2	-4.458	-21.973	5.32E-07	1.42E-05
KCNMA1	-4.452	-21.886	6.23E-08	2.26E-06
LGR5	-4.45	-21.86	0.001349	0.008321
CCDC136	-4.425	-21.483	4.22E-08	1.61E-06
ASPN	-4.42	-21.402	3.07E-06	6.17E-05
FAM65B	-4.413	-21.299	6.99E-06	0.000121
ERICH2	-4.41	-21.26	0.000153	0.001505
PHEX	-4.398	-21.078	0.001642	0.009754
TNC	-4.396	-21.058	9.70E-07	2.34E-05
ELN	-4.396	-21.05	3.60E-10	2.69E-08
CRISPLD2	-4.387	-20.925	5.74E-07	1.51E-05
KCNJ2	-4.386	-20.905	5.85E-06	0.000104
PROS1	-4.374	-20.742	3.96E-09	2.16E-07
PPARGC1A	-4.369	-20.657	1.22E-07	3.99E-06
FGF18	-4.366	-20.625	0.00072	0.005101
LOC101930611	-4.366	-20.619	0.000153	0.001503
CFI	-4.362	-20.568	1.04E-05	0.00017
DNM3OS	-4.355	-20.469	5.79E-12	6.91E-10
NA	-4.347	-20.345	7.12E-05	0.000796
PLXNC1	-4.335	-20.178	0.000574	0.004261
PPL	-4.333	-20.152	8.99E-09	4.26E-07
NA	-4.317	-19.926	4.14E-06	7.91E-05
NA	-4.317	-19.925	0.000325	0.002739
CTHRC1	-4.312	-19.864	3.19E-08	1.24E-06
NA	-4.31	-19.831	7.16E-07	1.83E-05
HHIP	-4.303	-19.738	3.28E-05	0.000426
SLC4A8	-4.299	-19.678	0.000406	0.003262
LTB4R2	-4.297	-19.662	6.27E-05	0.000715
SCG2	-4.295	-19.634	1.31E-05	0.000207
PLAT	-4.292	-19.594	1.01E-10	8.95E-09

NA	-4.286	-19.508	4.49E-08	1.69E-06
PINLYP	-4.272	-19.326	1.62E-05	0.000244
DSG2	-4.269	-19.279	0.005828	0.025951
MYEOV	-4.269	-19.277	0.000254	0.002242
MGP	-4.261	-19.167	8.31E-07	2.05E-05
CCL13	-4.26	-19.165	5.39E-05	0.000634
ITGA4	-4.253	-19.064	7.30E-08	2.61E-06
NR4A3	-4.252	-19.05	0.000523	0.00396
CCDC144B	-4.246	-18.981	0.000235	0.002118
NA	-4.219	-18.622	0.000388	0.003149
NA	-4.219	-18.619	8.03E-10	5.38E-08
PTGFR	-4.216	-18.582	1.19E-09	7.83E-08
EFHD1	-4.213	-18.54	2.84E-06	5.78E-05
EGR1	-4.211	-18.524	2.13E-07	6.36E-06
PRSS30P	-4.21	-18.51	0.002284	0.012591
CYS1	-4.209	-18.496	0.004289	0.020391
ANK1	-4.208	-18.487	0.004574	0.021421
MPP4	-4.201	-18.39	0.000217	0.001989
NA	-4.196	-18.331	1.75E-05	0.000259
MALL	-4.188	-18.223	0.000114	0.001176
SHANK2	-4.187	-18.212	9.29E-06	0.000155
CDA	-4.173	-18.033	0.000112	0.001163
PNMAL2	-4.166	-17.953	1.24E-08	5.67E-07
RERG	-4.163	-17.919	1.60E-09	1.02E-07
NA	-4.158	-17.85	0.003155	0.016169
C1RL-AS1	-4.151	-17.759	1.58E-07	4.95E-06
P2RX7	-4.15	-17.758	0.006268	0.027431
IQCA1	-4.146	-17.701	0.006018	0.026588
NFASC	-4.142	-17.659	5.96E-12	7.05E-10
VDR	-4.142	-17.651	2.46E-09	1.46E-07
SLC43A3	-4.141	-17.648	3.34E-09	1.89E-07
PDE1A	-4.141	-17.644	3.44E-18	1.80E-15
SLITRK4	-4.139	-17.621	0.001423	0.008677
LOC101928245	-4.13	-17.51	0.000658	0.00476
ADAMTSL1	-4.126	-17.461	1.60E-11	1.73E-09
LTF	-4.123	-17.428	0.007217	0.030689
CLDN11	-4.123	-17.422	5.22E-08	1.93E-06
SERPINE1	-4.118	-17.362	9.87E-08	3.34E-06
NOX4	-4.112	-17.291	5.83E-07	1.53E-05
STAC	-4.104	-17.191	1.21E-06	2.84E-05
GREB1L	-4.102	-17.173	2.63E-08	1.05E-06
RCAN2	-4.1	-17.149	1.58E-05	0.000241
NA	-4.099	-17.136	0.000239	0.002145
NA	-4.099	-17.135	8.01E-05	0.000874
ABCC2	-4.08	-16.907	9.69E-07	2.34E-05
NA	-4.079	-16.904	0.000152	0.001498
IL7	-4.076	-16.861	3.26E-05	0.000424
FAM19A5	-4.066	-16.745	1.86E-05	0.000271
PI16	-4.054	-16.612	0.000426	0.0034
SEMA3D	-4.051	-16.576	7.72E-05	0.000849

SEMA3E	-4.044	-16.495	0.000151	0.001493
NA	-4.044	-16.493	0.00201	0.011434
COL8A2	-4.043	-16.483	2.31E-06	4.87E-05
NEFM	-4.037	-16.419	7.69E-05	0.000847
LINC00312	-4.035	-16.398	3.29E-05	0.000427
LMO7-AS1	-4.018	-16.199	0.000247	0.002195
FAM20A	-3.997	-15.968	2.94E-16	8.34E-14
GRIN2D	-3.991	-15.902	9.27E-12	1.05E-09
APCDD1L-AS1	-3.99	-15.893	0.001695	0.009991
PDE1C	-3.983	-15.817	2.16E-09	1.31E-07
NA	-3.983	-15.81	2.69E-05	0.000366
LINC00856	-3.98	-15.784	0.000157	0.001538
APOL1	-3.958	-15.539	5.28E-15	1.20E-12
KIAA1644	-3.946	-15.417	1.61E-05	0.000244
FGF11	-3.94	-15.344	9.81E-05	0.001037
KCNK1	-3.938	-15.33	6.93E-05	0.00078
KLHL4	-3.935	-15.292	0.001319	0.008162
LOC100506123	-3.931	-15.252	1.01E-06	2.42E-05
THBS2	-3.926	-15.201	9.89E-09	4.59E-07
EVI2B	-3.909	-15.019	7.57E-05	0.000836
HOXC10	-3.906	-14.991	2.98E-14	6.05E-12
APOD	-3.904	-14.973	1.48E-11	1.63E-09
CLSPN	-3.902	-14.946	0.00017	0.001638
LAMC2	-3.899	-14.914	7.60E-12	8.84E-10
CORIN	-3.885	-14.779	0.000181	0.001728
STK32B	-3.885	-14.772	0.000534	0.004031
FRZB	-3.879	-14.713	1.47E-06	3.32E-05
FOSB	-3.875	-14.675	0.000452	0.003547
ABI3BP	-3.87	-14.621	1.50E-06	3.37E-05
SLC38A5	-3.863	-14.555	3.59E-10	2.69E-08
CYP1B1-AS1	-3.863	-14.554	0.000167	0.001615
DPT	-3.863	-14.552	3.11E-05	0.000408
TSPAN8	-3.861	-14.533	0.000214	0.00197
CXCL1	-3.855	-14.467	3.76E-07	1.03E-05
LAMA3	-3.855	-14.466	4.15E-06	7.91E-05
OAS2	-3.851	-14.434	3.27E-06	6.48E-05
NA	-3.848	-14.395	8.33E-05	0.000904
TMEM255B	-3.839	-14.31	3.81E-05	0.000482
TMEM130	-3.81	-14.022	0.000189	0.001786
NA	-3.809	-14.015	3.01E-05	0.000396
XAF1	-3.798	-13.912	1.47E-06	3.32E-05
CACNA1C	-3.796	-13.89	5.99E-11	5.66E-09
IL6	-3.77	-13.645	2.43E-05	0.000336
NA	-3.77	-13.644	0.001182	0.007465
PAPPA2	-3.767	-13.617	4.56E-06	8.57E-05
NA	-3.766	-13.605	0.000147	0.001459
MAPK13	-3.752	-13.471	9.84E-08	3.34E-06
SMPDL3A	-3.734	-13.304	1.54E-10	1.32E-08
EGR2	-3.734	-13.302	0.00098	0.006444
LIPC	-3.727	-13.243	0.004154	0.019905

GPRC5A	-3.725	-13.219	1.36E-06	3.14E-05
INPP4B	-3.723	-13.204	2.33E-07	6.87E-06
SULF1	-3.714	-13.123	1.15E-06	2.72E-05
NA	-3.71	-13.089	0.000526	0.003976
CLSTN2	-3.704	-13.03	0.000248	0.002203
CH25H	-3.697	-12.968	0.000117	0.001204
DOK6	-3.692	-12.928	5.69E-11	5.45E-09
MEG8	-3.69	-12.906	0.000468	0.003632
PPP1R13L	-3.688	-12.889	1.66E-07	5.17E-06
FOXF2	-3.688	-12.886	1.62E-08	7.04E-07
CELSR1	-3.686	-12.872	0.007478	0.031563
SLC9A7P1	-3.678	-12.801	0.000501	0.003818
AHNAK2	-3.676	-12.782	8.22E-05	0.000893
EXO1	-3.67	-12.73	0.000876	0.005904
NEBL	-3.655	-12.596	8.56E-08	3.00E-06
TRPV2	-3.65	-12.554	2.64E-11	2.74E-09
NA	-3.648	-12.533	0.000177	0.001694
NA	-3.645	-12.511	2.02E-06	4.34E-05
ITGBL1	-3.637	-12.437	2.60E-08	1.04E-06
ARMC9	-3.633	-12.405	2.54E-08	1.03E-06
TMEM59L	-3.632	-12.396	1.98E-13	3.26E-11
GPR150	-3.629	-12.374	0.000534	0.00403
LOC101927780	-3.628	-12.363	5.07E-05	0.000604
C1QTNF3	-3.626	-12.35	5.30E-06	9.65E-05
ACE	-3.615	-12.249	0.000289	0.002498
SECTM1	-3.607	-12.186	3.59E-10	2.69E-08
IFI27	-3.605	-12.169	1.70E-08	7.29E-07
ENPP4	-3.602	-12.144	3.94E-08	1.50E-06
PLCXD3	-3.6	-12.128	0.001981	0.011311
EDN1	-3.6	-12.128	0.005427	0.024529
NKD2	-3.599	-12.116	0.004675	0.021764
ANGPT2	-3.596	-12.091	0.007744	0.032421
CREB5	-3.593	-12.069	0.000343	0.002843
DAPK1	-3.592	-12.06	3.73E-06	7.24E-05
RNF112	-3.59	-12.046	4.53E-05	0.000553
ANKRD1	-3.587	-12.016	2.94E-07	8.34E-06
CENPF	-3.581	-11.969	2.71E-05	0.000367
CHST15	-3.581	-11.965	6.53E-08	2.36E-06
CYP1B1	-3.579	-11.948	2.75E-06	5.61E-05
PREX2	-3.573	-11.904	0.003115	0.015995
SLC2A3	-3.573	-11.904	1.93E-09	1.20E-07
TMEM119	-3.566	-11.845	8.55E-10	5.71E-08
CYP3A5	-3.565	-11.837	8.68E-05	0.000936
DCHS2	-3.565	-11.835	0.003622	0.017977
TMEM155	-3.559	-11.786	1.31E-05	0.000207
DNM1P46	-3.553	-11.733	0.000183	0.00174
ODF3B	-3.552	-11.725	2.88E-05	0.000385
TIAF1	-3.551	-11.72	4.94E-05	0.000594
BMPR1B	-3.548	-11.696	3.25E-05	0.000424
PODN	-3.543	-11.653	2.31E-12	3.00E-10

CHI3L2	-3.539	-11.628	0.000353	0.002909
MIR503HG	-3.539	-11.622	0.00128	0.007964
MUC1	-3.533	-11.577	1.85E-06	4.03E-05
RGS4	-3.532	-11.564	4.51E-09	2.41E-07
NA	-3.528	-11.536	1.09E-06	2.59E-05
ZC3H12B	-3.525	-11.508	4.99E-05	0.000597
VCAN	-3.524	-11.504	1.91E-08	8.03E-07
GPR1	-3.52	-11.469	0.000147	0.001461
ITGA11	-3.517	-11.45	6.82E-05	0.000769
NA	-3.515	-11.43	0.00631	0.027546
CEP55	-3.512	-11.405	0.000369	0.003022
SLC16A4	-3.51	-11.389	3.74E-07	1.03E-05
YPEL4	-3.505	-11.353	0.000406	0.003262
GAS6	-3.497	-11.288	3.03E-07	8.56E-06
TNFRSF11B	-3.481	-11.168	7.73E-10	5.21E-08
NDC80	-3.48	-11.159	0.00012	0.001222
LSAMP	-3.479	-11.151	1.60E-06	3.54E-05
FERMT3	-3.476	-11.127	0.000462	0.003594
NA	-3.465	-11.044	0.001183	0.007465
FBLN5	-3.454	-10.957	5.42E-10	3.88E-08
TGFBI	-3.453	-10.951	3.43E-10	2.62E-08
LRRC66	-3.452	-10.942	0.000298	0.002553
VWF	-3.451	-10.938	0.007804	0.032609
CRLF1	-3.444	-10.88	4.75E-10	3.46E-08
PCBP3	-3.444	-10.88	9.64E-09	4.49E-07
DGKH	-3.438	-10.84	2.50E-05	0.000343
ROBO2	-3.438	-10.837	0.000128	0.001293
BMF	-3.436	-10.826	5.34E-08	1.98E-06
ADAMTS10	-3.435	-10.815	9.20E-08	3.17E-06
NFATC4	-3.43	-10.78	4.85E-09	2.55E-07
SYNJ2	-3.429	-10.772	1.32E-06	3.06E-05
IER3	-3.429	-10.772	2.40E-06	5.01E-05
NCAPG	-3.429	-10.767	1.84E-05	0.00027
ABLIM1	-3.428	-10.765	1.81E-07	5.59E-06
PENK	-3.423	-10.726	2.29E-10	1.88E-08
AEBP1	-3.416	-10.676	1.28E-07	4.14E-06
DNM1	-3.412	-10.646	4.18E-06	7.97E-05
DDX47	-3.411	-10.634	0.000336	0.002799
CLSTN3	-3.404	-10.583	4.78E-11	4.64E-09
ABCA10	-3.396	-10.528	2.95E-05	0.000392
TMEM102	-3.391	-10.488	0.000334	0.002791
TK1	-3.387	-10.458	0.000864	0.005834
MATN3	-3.386	-10.458	2.86E-07	8.14E-06
ATP1B1	-3.386	-10.456	0.000188	0.001777
KCNQ1OT1	-3.385	-10.45	3.46E-06	6.81E-05
SNCAIP	-3.38	-10.411	0.001591	0.009522
AURKB	-3.379	-10.405	0.005413	0.024487
ADRA2C	-3.378	-10.396	4.87E-05	0.000585
CPXM1	-3.376	-10.382	0.008927	0.036145
RAB3B	-3.375	-10.372	1.11E-05	0.00018

GALNT12	-3.373	-10.359	0.000132	0.001327
STC2	-3.372	-10.356	1.52E-05	0.000233
CSRP2	-3.372	-10.351	1.54E-06	3.44E-05
RAB33A	-3.369	-10.335	9.71E-05	0.00103
AFF2	-3.368	-10.325	0.000385	0.003133
GPM6B	-3.368	-10.324	0.000194	0.001824
GNRH1	-3.367	-10.318	5.43E-06	9.86E-05
RARB	-3.364	-10.299	0.001716	0.010089
CLGN	-3.359	-10.258	0.001637	0.009727
KRT34	-3.354	-10.223	0.004675	0.021764
DCLK1	-3.347	-10.175	2.95E-09	1.68E-07
DTNA	-3.345	-10.163	1.27E-08	5.78E-07
HERC6	-3.344	-10.156	0.000508	0.003867
ARAP3	-3.344	-10.153	4.78E-06	8.87E-05
PKMYT1	-3.343	-10.149	0.007511	0.031665
SGIP1	-3.341	-10.136	0.001013	0.006618
SCNN1A	-3.339	-10.12	4.41E-05	0.000544
C2	-3.336	-10.096	0.000157	0.001544
SLC17A9	-3.336	-10.095	3.30E-05	0.000428
MAF	-3.335	-10.094	3.85E-08	1.48E-06
CENPK	-3.335	-10.091	0.001056	0.006827
NA	-3.334	-10.086	4.40E-05	0.000543
IGFBP3	-3.334	-10.082	3.08E-06	6.17E-05
OLFML2B	-3.33	-10.056	1.12E-07	3.71E-06
PRR15	-3.329	-10.051	0.000733	0.005171
NEGR1	-3.329	-10.05	2.78E-09	1.61E-07
LINC00578	-3.323	-10.006	0.001814	0.01052
MT2A	-3.318	-9.974	5.86E-09	2.95E-07
EVA1C	-3.317	-9.968	2.99E-05	0.000395
HRH1	-3.314	-9.945	2.96E-05	0.000393
TNFRSF1B	-3.313	-9.941	1.65E-06	3.64E-05
SPINT2	-3.313	-9.94	0.000633	0.004601
UBE2C	-3.308	-9.905	0.002191	0.012227
PRR16	-3.308	-9.903	6.41E-09	3.16E-07
THBS1	-3.304	-9.879	4.81E-05	0.000579
ZNF365	-3.298	-9.837	0.010367	0.040573
SERPINA3	-3.295	-9.814	6.61E-09	3.24E-07
ARL4C	-3.293	-9.799	0.000343	0.002843
KIAA1755	-3.291	-9.786	0.000815	0.005598
CCL28	-3.285	-9.746	7.20E-05	0.000803
LINC01018	-3.284	-9.743	0.000679	0.004863
ABCA9	-3.281	-9.721	1.53E-14	3.25E-12
LTBP2	-3.279	-9.709	6.00E-09	2.98E-07
OLFML2A	-3.276	-9.687	2.06E-06	4.40E-05
CACNB4	-3.275	-9.682	0.00078	0.005407
PRELID2	-3.27	-9.645	0.002316	0.012687
PRSS23	-3.268	-9.633	7.78E-07	1.95E-05
SCRG1	-3.262	-9.595	4.36E-08	1.65E-06
PRRX2	-3.262	-9.59	1.57E-07	4.91E-06
CLCF1	-3.257	-9.56	2.15E-05	0.000305

KCTD16	-3.254	-9.542	0.000162	0.001574
GGT5	-3.253	-9.536	7.13E-11	6.51E-09
SMAD9	-3.245	-9.48	8.58E-11	7.63E-09
NA	-3.241	-9.456	0.001444	0.008781
MEOX2	-3.239	-9.443	0.001728	0.010151
NA	-3.238	-9.433	0.000167	0.001615
NOSTRIN	-3.234	-9.408	0.00923	0.037071
UACA	-3.232	-9.395	2.88E-06	5.85E-05
BTN2A3P	-3.23	-9.383	6.08E-06	0.000108
MEG3	-3.225	-9.348	1.64E-05	0.000247
MRC2	-3.221	-9.326	1.04E-07	3.48E-06
LSP1	-3.22	-9.319	0.000312	0.002646
LOC344887	-3.22	-9.317	0.000198	0.001857
MSX2	-3.217	-9.297	0.000254	0.002245
SVEP1	-3.217	-9.296	3.27E-07	9.20E-06
KIF18B	-3.213	-9.272	0.005889	0.026136
TPTEP1	-3.21	-9.256	2.94E-10	2.30E-08
ALCAM	-3.21	-9.251	2.62E-06	5.36E-05
LBH	-3.204	-9.215	0.001286	0.007998
LRCH2	-3.203	-9.207	6.40E-06	0.000112
LMO7	-3.197	-9.168	6.16E-07	1.61E-05
KIRREL3	-3.196	-9.165	0.006109	0.026895
C2orf27A	-3.194	-9.154	4.71E-06	8.81E-05
CCL2	-3.194	-9.153	2.69E-06	5.51E-05
BMP6	-3.193	-9.146	4.98E-05	0.000597
ACTC1	-3.188	-9.112	8.55E-05	0.000925
PDCD1LG2	-3.186	-9.103	8.72E-07	2.14E-05
SAMD3	-3.185	-9.095	0.00569	0.025522
NA	-3.182	-9.074	6.09E-05	0.0007
NA	-3.176	-9.04	0.006301	0.027516
SBSPON	-3.175	-9.033	0.00382	0.018717
GRIA1	-3.169	-8.992	7.90E-07	1.97E-05
SPATA13	-3.165	-8.971	3.97E-06	7.65E-05
WEE2-AS1	-3.164	-8.961	0.000597	0.004387
ATL1	-3.163	-8.959	1.73E-06	3.79E-05
CCDC68	-3.163	-8.955	0.000279	0.002433
AADAC	-3.158	-8.925	1.94E-05	0.000281
BIRC3	-3.157	-8.922	4.07E-05	0.000509
BST2	-3.157	-8.918	0.002979	0.01549
HYDIN	-3.156	-8.913	3.46E-05	0.000445
SYTL2	-3.155	-8.906	6.97E-05	0.000783
CCDC74B	-3.15	-8.876	0.000114	0.001174
MUC20	-3.147	-8.857	0.00109	0.007009
PELI2	-3.144	-8.842	1.38E-06	3.18E-05
PODNL1	-3.143	-8.835	3.06E-08	1.20E-06
RNF139-AS1	-3.143	-8.835	0.00044	0.003485
APCDD1L	-3.143	-8.834	0.001183	0.007465
GDPD3	-3.143	-8.834	0.002004	0.011421
TTLL3	-3.143	-8.834	5.36E-09	2.75E-07
RUNX1	-3.139	-8.807	5.37E-08	1.98E-06

PDGFRL	-3.138	-8.805	7.29E-06	0.000125
COL12A1	-3.138	-8.802	9.42E-05	0.001005
SERPINE2	-3.133	-8.775	0.002551	0.013744
SLC8A1	-3.132	-8.764	5.71E-06	0.000102
FAT3	-3.13	-8.754	0.000278	0.002427
FMNL1	-3.127	-8.736	5.49E-05	0.000643
NA	-3.12	-8.695	3.63E-06	7.07E-05
LOC100289283	-3.119	-8.689	0.004201	0.020071
CLEC2D	-3.114	-8.659	0.000104	0.001095
HEG1	-3.113	-8.653	2.69E-07	7.75E-06
KIF14	-3.106	-8.611	0.00128	0.007964
TMTC1	-3.102	-8.588	6.82E-10	4.74E-08
MYO1D	-3.099	-8.569	1.03E-06	2.45E-05
IRF4	-3.096	-8.553	0.001109	0.007081
IFI16	-3.093	-8.533	6.51E-10	4.59E-08
DAW1	-3.091	-8.523	0.002724	0.01446
DUSP5	-3.091	-8.519	3.14E-06	6.26E-05
CDON	-3.078	-8.444	2.19E-08	9.06E-07
FLJ22447	-3.077	-8.44	0.000522	0.003957
LOC101930107	-3.076	-8.432	0.000229	0.002072
DPP4	-3.071	-8.402	0.000928	0.006178
KCNJ12	-3.07	-8.4	0.000992	0.006506
PAQR8	-3.07	-8.398	5.14E-05	0.000611
OSR2	-3.066	-8.375	3.22E-13	5.04E-11
KCNC4	-3.066	-8.373	2.20E-05	0.00031
SNED1	-3.063	-8.357	2.15E-10	1.78E-08
FHL2	-3.057	-8.321	4.91E-09	2.56E-07
CFHR1	-3.054	-8.305	0.000365	0.002995
ZNF385D	-3.053	-8.302	7.32E-05	0.000813
PRTFDC1	-3.052	-8.293	3.28E-07	9.22E-06
NA	-3.046	-8.259	0.002644	0.014134
XG	-3.039	-8.216	2.48E-09	1.46E-07
PLSCR1	-3.038	-8.215	7.29E-06	0.000125
KIAA1324	-3.038	-8.213	0.003346	0.016961
OGN	-3.035	-8.197	1.96E-07	5.98E-06
PTX3	-3.03	-8.169	1.14E-05	0.000183
LRRC32	-3.029	-8.163	9.89E-06	0.000163
FAM227B	-3.029	-8.162	0.003422	0.017245
PLA2G2A	-3.028	-8.155	0.000497	0.003793
PDGFD	-3.023	-8.126	7.03E-06	0.000122
CCDC180	-3.021	-8.116	0.00114	0.007252
ADCY3	-3.02	-8.112	1.20E-08	5.53E-07
CDH11	-3.017	-8.096	2.61E-06	5.36E-05
ROR2	-3.017	-8.096	8.66E-08	3.03E-06
LRRC6	-3.016	-8.089	6.05E-06	0.000107
ACVR2A	-3.015	-8.085	3.41E-09	1.91E-07
LINC01503	-3.012	-8.066	1.99E-05	0.000286
CHRD	-3.01	-8.057	0.000314	0.002658
MTMR11	-3.006	-8.035	3.00E-05	0.000396
PDGFRA	-3.005	-8.028	3.66E-07	1.01E-05

HMGB2	-3.004	-8.024	8.05E-09	3.87E-07
CLU	-3.004	-8.024	1.45E-05	0.000225
FAT4	-3.002	-8.011	3.81E-07	1.04E-05
EFHC1	-3.001	-8.007	5.02E-08	1.87E-06
STARD9	-3.001	-8.005	8.07E-07	2.01E-05
TMEM171	-2.999	-7.992	0.00258	0.013872
SOCS2	-2.994	-7.968	7.07E-07	1.81E-05
CYR61	-2.994	-7.964	1.28E-06	2.98E-05
NA	-2.993	-7.964	7.84E-05	0.000859
ZNF704	-2.991	-7.95	9.59E-09	4.48E-07
RUNX2	-2.985	-7.919	2.81E-07	8.06E-06
HERC2P3	-2.984	-7.913	0.000334	0.002794
CYP27A1	-2.983	-7.905	7.30E-10	5.00E-08
ALPK2	-2.978	-7.881	9.86E-06	0.000163
SBSN	-2.977	-7.872	0.002753	0.014575
DRAM1	-2.974	-7.855	9.73E-08	3.32E-06
CTSH	-2.973	-7.854	0.000459	0.003579
LOC440028	-2.971	-7.84	3.30E-05	0.000427
SLC40A1	-2.969	-7.831	1.21E-06	2.84E-05
EVA1A	-2.965	-7.806	0.002221	0.012359
FOXP2	-2.953	-7.743	1.02E-05	0.000167
KIF20A	-2.949	-7.721	0.000421	0.003364
NFKBIZ	-2.949	-7.72	6.09E-08	2.22E-06
NA	-2.949	-7.72	0.000969	0.006387
LOC100653061	-2.948	-7.714	0.000121	0.001235
CBLN3	-2.942	-7.683	7.28E-06	0.000125
TSHZ2	-2.941	-7.681	4.87E-09	2.55E-07
FCHSD1	-2.941	-7.678	8.81E-07	2.15E-05
FGF13	-2.94	-7.675	0.000671	0.004831
Mar-04	-2.939	-7.669	0.000122	0.001241
COL8A1	-2.938	-7.664	0.000375	0.003067
GPR176	-2.936	-7.654	3.36E-07	9.39E-06
RIMS1	-2.935	-7.647	0.012716	0.047291
SCARA5	-2.934	-7.64	0.006296	0.02751
NA	-2.933	-7.636	0.006924	0.029656
ELF4	-2.933	-7.636	9.91E-08	3.35E-06
NA	-2.928	-7.613	0.000902	0.006048
CORO2B	-2.926	-7.599	4.08E-12	5.00E-10
RUNX1T1	-2.925	-7.596	5.63E-09	2.87E-07
MGAM	-2.924	-7.592	0.002424	0.013166
PRRX1	-2.923	-7.583	6.71E-08	2.41E-06
CD1D	-2.922	-7.579	0.001411	0.00862
DISP2	-2.92	-7.57	0.013222	0.04874
TRANK1	-2.918	-7.557	8.42E-09	4.02E-07
INMT	-2.916	-7.549	6.21E-05	0.000711
PSMB9	-2.913	-7.531	2.25E-05	0.000315
SYNE1	-2.91	-7.515	4.69E-07	1.26E-05
PLAU	-2.908	-7.506	1.43E-06	3.27E-05
FRMD6	-2.904	-7.486	9.62E-07	2.33E-05
THRB	-2.902	-7.475	2.65E-10	2.11E-08

LGALS9	-2.901	-7.468	0.000327	0.002748
TIMP3	-2.901	-7.467	2.21E-07	6.58E-06
NA	-2.9	-7.464	0.006269	0.027431
LRRC49	-2.893	-7.428	4.29E-05	0.000532
EFEMP1	-2.889	-7.409	1.25E-07	4.06E-06
COL16A1	-2.887	-7.397	9.83E-07	2.36E-05
MYH15	-2.881	-7.365	0.003329	0.016898
HGF	-2.88	-7.364	4.10E-06	7.86E-05
FBLN1	-2.88	-7.36	1.47E-07	4.64E-06
UNC5B	-2.88	-7.36	5.88E-05	0.00068
LOC101929295	-2.877	-7.346	0.000441	0.003487
ETS1	-2.874	-7.332	2.67E-07	7.71E-06
ACKR2	-2.874	-7.331	0.005258	0.023938
FN1	-2.873	-7.326	1.26E-05	0.0002
DNM3	-2.868	-7.302	0.000288	0.002498
C6orf132	-2.865	-7.287	5.07E-05	0.000604
NA	-2.864	-7.281	0.002574	0.013842
FMOD	-2.864	-7.281	1.28E-08	5.84E-07
NMNAT2	-2.864	-7.279	8.55E-07	2.11E-05
NRK	-2.861	-7.265	0.0017	0.010009
TMEM173	-2.859	-7.253	6.21E-08	2.25E-06
PTGS1	-2.856	-7.242	1.55E-08	6.78E-07
ASPM	-2.856	-7.238	0.000548	0.004099
PRICKLE1	-2.855	-7.236	1.83E-10	1.53E-08
NEB	-2.855	-7.233	0.001527	0.009201
ITGB3	-2.855	-7.233	1.32E-05	0.000207
TRIM47	-2.854	-7.232	0.000997	0.006525
ELTD1	-2.847	-7.195	5.18E-06	9.47E-05
KIFC1	-2.845	-7.186	0.00295	0.015371
NTRK2	-2.845	-7.184	0.000759	0.005304
COL21A1	-2.834	-7.129	5.71E-07	1.51E-05
SLC23A3	-2.832	-7.119	0.000732	0.005164
IFI44	-2.831	-7.115	7.64E-08	2.72E-06
TCEAL7	-2.826	-7.091	0.000276	0.002408
NRG1	-2.826	-7.091	0.003395	0.017157
COL14A1	-2.823	-7.074	1.27E-07	4.10E-06
SLC12A8	-2.811	-7.018	0.000816	0.005599
LTBP1	-2.81	-7.011	6.09E-06	0.000108
SEC14L2	-2.809	-7.01	1.82E-05	0.000268
PTPRH	-2.809	-7.01	0.003973	0.019282
OLFM1	-2.808	-7.001	2.45E-07	7.16E-06
NA	-2.804	-6.982	0.000387	0.003144
CASP10	-2.798	-6.954	4.79E-06	8.87E-05
KCNIP3	-2.794	-6.934	0.001247	0.007785
GOLGA8A	-2.793	-6.931	1.53E-09	9.82E-08
PID1	-2.793	-6.93	1.89E-09	1.18E-07
TRAM1L1	-2.79	-6.918	0.000243	0.002168
CTSS	-2.79	-6.914	0.000456	0.00356
LAMA2	-2.789	-6.914	3.52E-06	6.89E-05
NA	-2.789	-6.912	0.000673	0.004831

DPYSL3	-2.789	-6.91	4.75E-05	0.000574
SPON2	-2.784	-6.888	5.59E-06	0.000101
N4BP2L1	-2.783	-6.882	2.87E-05	0.000385
NA	-2.782	-6.879	0.00785	0.032778
FAM131B	-2.781	-6.875	1.47E-05	0.000227
NA	-2.78	-6.868	0.004633	0.021634
ZMIZ1-AS1	-2.779	-6.865	0.000338	0.002812
PPP4R4	-2.768	-6.811	0.006251	0.027375
SAMHD1	-2.766	-6.804	0.000796	0.005496
GPR173	-2.762	-6.783	1.61E-05	0.000244
NA	-2.762	-6.781	0.000741	0.005203
SCG5	-2.759	-6.768	0.003825	0.018737
NA	-2.758	-6.765	0.000774	0.005374
APOBEC3D	-2.756	-6.755	0.000188	0.00178
CCDC170	-2.756	-6.754	2.06E-05	0.000295
NA	-2.755	-6.751	0.000295	0.002536
NPL	-2.747	-6.714	9.58E-05	0.00102
RECK	-2.747	-6.712	4.30E-06	8.17E-05
FZD3	-2.744	-6.7	0.003495	0.017518
MBOAT2	-2.739	-6.674	1.70E-08	7.29E-07
RELL2	-2.735	-6.658	0.000535	0.004031
BTBD19	-2.735	-6.657	6.62E-05	0.000749
CD40	-2.727	-6.619	0.00027	0.002366
GFPT2	-2.726	-6.617	2.74E-09	1.59E-07
ITPRIP	-2.722	-6.6	3.85E-09	2.11E-07
INHBA	-2.722	-6.599	0.000136	0.001359
NXPH3	-2.721	-6.593	0.00062	0.004529
PLXDC1	-2.719	-6.584	0.001546	0.009299
KITLG	-2.718	-6.58	3.56E-08	1.38E-06
FBXO32	-2.717	-6.576	0.000236	0.002118
HELLS	-2.717	-6.575	0.000253	0.002238
LOC440416	-2.716	-6.571	0.005063	0.023198
MOV10L1	-2.715	-6.568	0.008771	0.035673
ITGB8	-2.713	-6.558	0.001315	0.008144
PDXDC2P	-2.711	-6.549	6.62E-05	0.000749
TEK	-2.711	-6.547	0.005275	0.023976
SIPA1L2	-2.707	-6.528	1.66E-05	0.000249
SLC37A1	-2.706	-6.524	0.000723	0.005119
GABBR1	-2.695	-6.476	3.81E-09	2.10E-07
C12orf75	-2.694	-6.47	5.17E-06	9.47E-05
GPRIN1	-2.693	-6.467	0.000911	0.006078
LINC01111	-2.691	-6.458	0.001899	0.010914
RSRP1	-2.686	-6.434	2.49E-09	1.46E-07
ELANE	-2.685	-6.43	0.004597	0.02148
BICC1	-2.683	-6.423	2.64E-07	7.64E-06
MYOZ2	-2.683	-6.42	0.008175	0.033794
MLPH	-2.678	-6.4	0.001574	0.009426
BUB1B	-2.677	-6.396	0.002724	0.01446
STXBP6	-2.677	-6.394	6.25E-05	0.000714
IL6R	-2.672	-6.374	1.58E-06	3.53E-05

AARD	-2.671	-6.37	0.001872	0.0108
KCNK2	-2.67	-6.363	6.71E-08	2.41E-06
NUSAP1	-2.669	-6.361	0.001534	0.009239
MOXD1	-2.666	-6.348	4.27E-05	0.00053
CCBE1	-2.666	-6.347	3.47E-06	6.81E-05
MEDAG	-2.666	-6.347	1.38E-08	6.17E-07
NA	-2.665	-6.343	0.004333	0.020566
NBL1	-2.664	-6.338	8.17E-08	2.88E-06
CCDC39	-2.663	-6.333	0.001095	0.007025
CYGB	-2.663	-6.332	9.17E-06	0.000153
LINC01119	-2.662	-6.328	0.000895	0.006016
NEK10	-2.661	-6.326	0.002396	0.013053
TMEM204	-2.659	-6.314	2.65E-07	7.64E-06
ACSS1	-2.656	-6.303	0.000456	0.00356
KCNE4	-2.656	-6.303	9.89-05	0.001044
DCHS1	-2.654	-6.293	0.00316	0.016191
LY96	-2.649	-6.273	7.19E-06	0.000124
GRAMD1C	-2.649	-6.273	0.00928	0.037219
ABCC3	-2.648	-6.268	1.25E-10	1.09E-08
LRRC2	-2.647	-6.264	0.00242	0.013158
DSE	-2.645	-6.257	1.33E-07	4.28E-06
C10orf11	-2.644	-6.25	1.90E-05	0.000277
AXL	-2.644	-6.249	7.64E-05	0.000843
COL1A1	-2.638	-6.223	0.000907	0.006069
SLC44A3	-2.637	-6.221	0.000199	0.00186
APOB	-2.635	-6.21	0.003962	0.019248
B3GALT2	-2.633	-6.204	0.001143	0.007267
BAIAP2L2	-2.633	-6.203	0.001563	0.009371
LOC154761	-2.632	-6.197	0.003769	0.018537
MYO18A	-2.629	-6.186	7.22E-07	1.84E-05
NAALADL1	-2.627	-6.179	6.11E-05	0.000701
ASPHD2	-2.619	-6.143	0.001911	0.010969
SPOCD1	-2.618	-6.137	4.76E-06	8.87E-05
EPHB3	-2.61	-6.106	0.000216	0.001986
SLC22A15	-2.603	-6.077	0.000273	0.002387
B3GNT8	-2.599	-6.058	0.000784	0.005429
TMSB15B	-2.598	-6.055	0.000981	0.006451
WNT11	-2.598	-6.054	0.000747	0.005243
MAP3K7CL	-2.594	-6.037	0.010125	0.039765
ISLR	-2.589	-6.019	4.48E-07	1.21E-05
NA	-2.589	-6.017	0.00169	0.009971
HTR2A	-2.588	-6.013	0.000162	0.001574
HAGLR	-2.588	-6.011	0.006237	0.027342
GUCY1A2	-2.587	-6.009	0.001392	0.008537
INHA	-2.586	-6.006	0.00348	0.017474
GLI2	-2.586	-6.003	5.89E-09	2.96E-07
CFH	-2.585	-6	6.56E-07	1.69E-05
RTKN2	-2.583	-5.99	0.004848	0.022433
MX2	-2.58	-5.98	0.000112	0.00116
PABPC5	-2.578	-5.972	6.22E-05	0.000712

TMEM200A	-2.576	-5.962	6.80E-07	1.75E-05
THTPA	-2.575	-5.959	0.000333	0.002787
PLEKHG4	-2.574	-5.953	8.00E-05	0.000874
TCTN2	-2.572	-5.948	2.89E-06	5.85E-05
ANXA3	-2.567	-5.926	0.01178	0.044592
GABRB3	-2.566	-5.923	0.004525	0.021247
C1RL	-2.565	-5.919	1.34E-09	8.70E-08
HP	-2.558	-5.89	0.011555	0.043994
IL1R1	-2.555	-5.876	5.88E-05	0.00068
GALNT5	-2.553	-5.87	0.000241	0.002157
FLNC	-2.551	-5.86	2.91E-05	0.000389
GDF15	-2.551	-5.859	0.009477	0.037865
PTPRN	-2.541	-5.821	0.000348	0.002872
SLC6A16	-2.54	-5.814	0.00283	0.014879
NDRG1	-2.538	-5.806	8.18E-07	2.03E-05
MEG9	-2.534	-5.794	0.000984	0.006459
CYP2R1	-2.533	-5.787	2.12E-05	0.000301
CMAHP	-2.533	-5.787	5.10E-06	9.37E-05
DDX60	-2.532	-5.785	1.11E-05	0.00018
TPM1	-2.531	-5.779	2.25E-05	0.000315
SHROOM4	-2.531	-5.779	0.0009	0.006039
NA	-2.527	-5.765	0.001297	0.008047
NA	-2.526	-5.761	0.004132	0.019834
MTMR9LP	-2.525	-5.755	0.003697	0.018255
LRRCC1	-2.521	-5.741	0.000172	0.00165
BACH2	-2.518	-5.728	0.005548	0.024976
CLHC1	-2.518	-5.726	0.00015	0.001481
NA	-2.517	-5.724	0.004064	0.019597
MYLK	-2.515	-5.718	0.001029	0.006693
FAM115C	-2.515	-5.714	0.000211	0.00195
LMCD1	-2.512	-5.704	0.000246	0.002193
BOC	-2.512	-5.703	6.20E-10	4.42E-08
FKBP1B	-2.511	-5.7	0.001796	0.010457
LOC100506258	-2.51	-5.695	0.001219	0.007631
ADCY1	-2.509	-5.692	0.001169	0.007396
DCN	-2.509	-5.692	1.41E-05	0.000219
LAYN	-2.507	-5.684	1.87E-06	4.06E-05
NA	-2.507	-5.683	0.004154	0.019905
SLFN11	-2.504	-5.671	1.58E-06	3.53E-05
LINC00565	-2.502	-5.665	0.000446	0.003505
FZD1	-2.495	-5.638	2.50E-07	7.30E-06
LOC102724814	-2.492	-5.624	3.96E-07	1.08E-05
GBAP1	-2.491	-5.622	0.000903	0.006049
PSORS1C1	-2.49	-5.616	0.001778	0.010386
KIF15	-2.489	-5.615	0.005687	0.025516
TRPV4	-2.488	-5.608	6.18E-05	0.000709
MIR100HG	-2.488	-5.608	4.04E-07	1.10E-05
NA	-2.484	-5.595	7.01E-05	0.000786
MYOF	-2.483	-5.59	2.15E-05	0.000304
FST	-2.479	-5.577	6.39E-07	1.65E-05

PTPRB	-2.479	-5.574	0.006894	0.029559
GSTM5	-2.476	-5.562	0.00213	0.011936
CALHM2	-2.473	-5.551	2.34E-07	6.88E-06
DST	-2.471	-5.546	6.53E-06	0.000114
CSMD2	-2.47	-5.54	2.10E-05	0.000299
MARK1	-2.468	-5.534	0.002066	0.011661
GPC3	-2.465	-5.521	1.46E-07	4.62E-06
ANKRD36B	-2.463	-5.513	0.000988	0.006484
KRT7	-2.462	-5.509	0.00025	0.002219
SLC25A37	-2.457	-5.492	1.82E-07	5.62E-06
PTPRJ	-2.457	-5.49	2.75E-05	0.00037
ENO2	-2.457	-5.489	9.63E-05	0.001023
LOC613037	-2.454	-5.479	0.000117	0.001202
LPAR1	-2.453	-5.476	1.32E-06	3.07E-05
CTSK	-2.452	-5.471	4.03E-05	0.000505
CACNB1	-2.449	-5.459	0.000335	0.002794
ZNF469	-2.449	-5.459	9.02E-05	0.000968
ABCA6	-2.448	-5.455	4.41E-09	2.36E-07
PSMB8	-2.447	-5.452	1.23E-07	4.02E-06
MMP16	-2.445	-5.445	0.000145	0.001442
AFF3	-2.444	-5.443	0.005163	0.023609
LOX	-2.443	-5.436	1.28E-05	0.000203
AHNAK	-2.44	-5.426	1.16E-05	0.000185
IL1RL2	-2.439	-5.423	0.001804	0.010482
NA	-2.438	-5.419	0.000994	0.006511
FBLN7	-2.434	-5.406	5.13E-05	0.00061
ANKRD6	-2.427	-5.378	1.84E-05	0.00027
ITGA2	-2.426	-5.375	0.000211	0.001952
ADCY7	-2.422	-5.359	4.33E-06	8.21E-05
ZC4H2	-2.422	-5.358	8.06E-06	0.000136
BAALC-AS1	-2.421	-5.354	0.001144	0.007272
ID3	-2.42	-5.351	3.01E-05	0.000396
IFITM2	-2.42	-5.351	6.18E-06	0.000109
COL1A2	-2.418	-5.344	0.000243	0.002168
ATHL1	-2.417	-5.342	5.63E-06	0.000101
PFKP	-2.415	-5.335	2.01E-08	8.40E-07
ODF2L	-2.411	-5.317	5.00E-07	1.34E-05
FAM129A	-2.407	-5.304	0.001203	0.007553
USP53	-2.406	-5.298	5.50E-05	0.000644
SYT11	-2.405	-5.296	2.41E-05	0.000335
NNMT	-2.405	-5.295	2.13E-05	0.000303
C9orf3	-2.402	-5.286	5.83E-06	0.000104
LINC01354	-2.402	-5.285	0.000956	0.006323
NDNF	-2.399	-5.276	9.96E-06	0.000164
HLA-DPB1	-2.399	-5.274	0.001913	0.010975
HIP1	-2.397	-5.267	5.15E-07	1.38E-05
RAMP1	-2.396	-5.264	0.003102	0.015959
ETV5	-2.395	-5.261	0.002089	0.011759
NA	-2.392	-5.25	0.002337	0.012776
TRO	-2.39	-5.242	5.75E-05	0.000667

LINC00941	-2.389	-5.238	0.004195	0.020071
DLG3	-2.388	-5.235	0.000327	0.002748
DTL	-2.387	-5.231	0.002836	0.014898
ZSWIM4	-2.387	-5.23	0.000784	0.005432
MIR29A	-2.386	-5.227	0.000706	0.005024
HSPB7	-2.383	-5.216	0.000176	0.001688
CEP152	-2.382	-5.214	0.000161	0.00157
FAM84B	-2.381	-5.211	0.00421	0.020101
MXD3	-2.381	-5.208	0.002022	0.011471
MAP3K12	-2.38	-5.207	2.28E-08	9.37E-07
SOCS3	-2.38	-5.207	0.001662	0.009845
ANKRD36	-2.375	-5.187	0.000367	0.003008
NA	-2.374	-5.185	0.002493	0.013478
NPHP1	-2.372	-5.175	0.000589	0.004344
HLA-B	-2.371	-5.173	2.46E-08	1.00E-06
NA	-2.37	-5.17	0.001887	0.010877
CCDC146	-2.369	-5.167	0.000112	0.00116
HIVEP1	-2.364	-5.149	4.92E-07	1.32E-05
TRPC1	-2.363	-5.146	1.37E-05	0.000214
GCNT1	-2.363	-5.144	0.000216	0.001987
EDNRB	-2.363	-5.143	0.000761	0.005316
LNX1	-2.362	-5.141	0.002316	0.012687
NA	-2.362	-5.141	0.002618	0.014024
SHCBP1	-2.361	-5.137	0.005411	0.024487
TNFRSF14	-2.36	-5.134	0.000191	0.001799
PYCARD	-2.359	-5.131	0.000203	0.001888
TMEM98	-2.357	-5.123	2.70E-08	1.08E-06
TSHZ3	-2.356	-5.119	6.57E-06	0.000115
CROCCP3	-2.356	-5.118	0.000196	0.001844
LOC100506834	-2.355	-5.117	0.00115	0.007298
NAP1L3	-2.355	-5.116	0.000459	0.003575
FIBIN	-2.355	-5.115	4.50E-08	1.69E-06
NA	-2.354	-5.112	0.0006	0.004408
ARHGEF25	-2.353	-5.107	0.00043	0.003423
GLIPR1	-2.35	-5.099	1.31E-07	4.22E-06
FOS	-2.35	-5.098	0.004055	0.019574
DDX26B	-2.349	-5.096	0.000136	0.001359
SDHAP2	-2.349	-5.093	1.89E-06	4.10E-05
PARP14	-2.346	-5.085	3.12E-07	8.78E-06
CXCL6	-2.346	-5.083	0.004332	0.020566
FAM111A	-2.345	-5.08	2.59E-07	7.53E-06
PLS3	-2.34	-5.063	1.84E-05	0.00027
BASP1	-2.338	-5.056	0.000291	0.00251
KCNS2	-2.338	-5.055	0.001161	0.00735
DKK3	-2.337	-5.051	1.36E-06	3.14E-05
SHC3	-2.334	-5.043	0.008748	0.035618
ANPEP	-2.334	-5.042	5.58E-06	0.000101
SLC7A7	-2.332	-5.036	0.002064	0.011656
PIP5KL1	-2.331	-5.033	0.013151	0.048523
HSD17B11	-2.331	-5.033	3.56E-07	9.90E-06

TTC21A	-2.327	-5.019	0.000626	0.00457
IER5L	-2.327	-5.018	0.001177	0.007445
DAB2	-2.327	-5.017	7.10E-05	0.000795
SERP2	-2.321	-4.996	0.002081	0.011729
CBX7	-2.32	-4.994	2.33E-06	4.90E-05
SPEG	-2.32	-4.992	4.95E-06	9.13E-05
ADAM22	-2.318	-4.988	0.00235	0.012839
PAQR5	-2.316	-4.981	0.008448	0.0347
LOC100506718	-2.316	-4.978	0.000156	0.001536
GPR135	-2.314	-4.973	0.000398	0.003224
CECR7	-2.312	-4.965	0.0011	0.007053
FBN1	-2.311	-4.963	1.58E-05	0.000241
NA	-2.308	-4.953	0.001093	0.007017
ADAM33	-2.308	-4.95	6.10E-05	0.000701
NABP1	-2.306	-4.944	4.55E-06	8.56E-05
EMP3	-2.303	-4.935	0.000144	0.001435
BTN2A2	-2.302	-4.931	0.000107	0.001122
IGSF10	-2.298	-4.917	0.006281	0.027463
PTPRD	-2.296	-4.91	1.01E-05	0.000165
C11orf96	-2.293	-4.902	0.005857	0.026035
SWAP70	-2.293	-4.901	4.05E-05	0.000508
EYA2	-2.293	-4.9	2.68E-05	0.000364
NA	-2.293	-4.899	0.000347	0.002867
NA	-2.287	-4.882	0.002405	0.013086
RENBP	-2.286	-4.877	0.012578	0.046905
PKD1L2	-2.285	-4.872	1.05E-05	0.000171
TGIF1	-2.284	-4.87	7.26E-07	1.84E-05
THBS3	-2.282	-4.862	4.38E-07	1.18E-05
LIMCH1	-2.28	-4.858	0.001136	0.007232
LY6G5B	-2.277	-4.845	0.002646	0.014136
ZNF37BP	-2.276	-4.845	0.000125	0.001265
GAS1	-2.273	-4.832	8.25E-06	0.000139
ARMCX2	-2.27	-4.825	7.97E-05	0.000872
RPL32P3	-2.267	-4.812	1.55E-05	0.000237
TC2N	-2.267	-4.812	0.011286	0.043309
ID1	-2.265	-4.806	0.007463	0.031518
NA	-2.265	-4.805	0.004882	0.02256
STK32A	-2.259	-4.787	0.000534	0.00403
LINC-PINT	-2.259	-4.786	0.000307	0.002616
NMI	-2.259	-4.785	0.000457	0.003566
NYNRIN	-2.257	-4.779	0.001081	0.006957
ICAM1	-2.256	-4.778	0.000993	0.006509
ACAN	-2.255	-4.773	5.37E-06	9.76E-05
DACT1	-2.254	-4.771	0.000824	0.005631
MT1L	-2.254	-4.77	0.000809	0.005565
NA	-2.254	-4.77	0.004576	0.021421
PSRC1	-2.254	-4.769	0.00425	0.020251
MELK	-2.254	-4.769	0.00045	0.003534
SSTR1	-2.253	-4.767	0.008862	0.035956
LXN	-2.253	-4.766	0.000468	0.00363

TPM4	-2.251	-4.762	0.000226	0.00205
EMX2OS	-2.251	-4.76	1.66E-05	0.000249
FAM13A-AS1	-2.251	-4.759	0.004526	0.021247
MAN1C1	-2.25	-4.758	4.35E-06	8.23E-05
FAM19A2	-2.25	-4.756	0.000387	0.003144
SFRP4	-2.249	-4.752	0.000157	0.001543
GDPD1	-2.247	-4.748	0.01363	0.049925
GATA6	-2.247	-4.746	0.000608	0.004454
ITGB5	-2.247	-4.745	1.74E-05	0.000257
AK5	-2.246	-4.743	0.0042	0.020071
C7orf31	-2.244	-4.736	0.000855	0.005791
TSPAN11	-2.243	-4.735	0.001483	0.008974
CBWD5	-2.24	-4.723	0.002856	0.014986
ITIH3	-2.239	-4.721	0.002993	0.015544
GLT8D2	-2.238	-4.716	2.60E-08	1.04E-06
NA	-2.237	-4.715	0.005446	0.024574
LMO3	-2.234	-4.704	0.000158	0.001548
AHRR	-2.232	-4.699	8.28E-05	0.0009
MSS51	-2.232	-4.699	0.004105	0.019755
SLC44A1	-2.23	-4.691	5.35E-05	0.00063
OSBPL3	-2.229	-4.689	0.004506	0.02119
ADAMTS1	-2.227	-4.682	0.000157	0.001544
KCNJ8	-2.226	-4.677	1.54E-05	0.000236
EBF2	-2.225	-4.677	7.17E-07	1.83E-05
VASN	-2.224	-4.673	0.000264	0.002322
ACSS3	-2.224	-4.672	3.61E-07	1.00E-05
LOC284454	-2.223	-4.668	0.000557	0.004157
ABCC9	-2.22	-4.66	2.96E-05	0.000394
HNRNPU-AS1	-2.22	-4.657	4.70E-06	8.79E-05
GPR124	-2.211	-4.63	5.18E-06	9.47E-05
LINC00857	-2.209	-4.625	0.00439	0.02078
ARHGDIB	-2.205	-4.61	4.65E-06	8.72E-05
ULBP1	-2.205	-4.609	0.006949	0.029733
LCA5	-2.203	-4.606	0.000534	0.00403
STX3	-2.201	-4.598	0.000161	0.00157
CCND1	-2.199	-4.593	0.001262	0.007869
KIF23	-2.198	-4.59	0.001611	0.009622
ATP8B2	-2.198	-4.588	1.91E-05	0.000278
IRF9	-2.195	-4.579	0.002259	0.012494
METRNL	-2.194	-4.576	9.19E-05	0.000983
PRSS12	-2.193	-4.574	0.009764	0.038738
PIK3IP1	-2.192	-4.57	8.68E-06	0.000146
ZNF662	-2.191	-4.566	0.000172	0.00165
RASSF2	-2.19	-4.564	0.011406	0.043614
CCDC14	-2.189	-4.56	1.98E-06	4.26E-05
LTB4R	-2.188	-4.557	0.000181	0.001728
KIF4A	-2.188	-4.556	0.011138	0.042789
IFITM3	-2.185	-4.547	7.69E-05	0.000847
APOBEC3G	-2.183	-4.542	0.000542	0.004066
OLFML3	-2.183	-4.54	2.24E-05	0.000315

TRAF5	-2.182	-4.537	0.000135	0.00135
CSGALNACT2	-2.179	-4.529	3.05E-06	6.15E-05
MYADM	-2.178	-4.524	0.000443	0.003495
TG	-2.177	-4.524	0.002876	0.01508
SLFN12	-2.177	-4.522	0.000177	0.001694
FUT8	-2.176	-4.52	3.97E-05	0.0005
AMT	-2.175	-4.517	2.89E-05	0.000386
FAM227A	-2.174	-4.513	0.004287	0.020391
ROBO3	-2.174	-4.512	0.00185	0.010685
NA	-2.171	-4.505	0.004023	0.019462
LUM	-2.171	-4.503	0.000133	0.001337
CADPS2	-2.168	-4.493	0.001475	0.008938
IL4R	-2.166	-4.489	2.70E-05	0.000366
NR2F1-AS1	-2.161	-4.471	0.000609	0.004459
INTS6-AS1	-2.159	-4.467	0.005655	0.025396
ARMC12	-2.159	-4.467	0.003926	0.019122
MYH2	-2.156	-4.456	0.008104	0.033595
PABPC1L	-2.154	-4.452	0.000431	0.003432
LSMEM1	-2.154	-4.45	0.005518	0.024863
CLIC2	-2.152	-4.445	0.011206	0.043029
CPT1C	-2.15	-4.439	1.42E-05	0.000221
NA	-2.149	-4.435	0.000774	0.005374
HSF4	-2.148	-4.431	0.001614	0.009622
EBLN2	-2.147	-4.428	0.007734	0.032392
IFT80	-2.146	-4.426	1.92E-05	0.000279
LINC00960	-2.144	-4.421	0.000151	0.001493
WDR66	-2.144	-4.42	0.001069	0.006896
FOXM1	-2.144	-4.419	0.006483	0.028157
ABCA8	-2.141	-4.409	8.70E-06	0.000146
NA	-2.139	-4.404	0.012583	0.046911
DPEP1	-2.139	-4.403	0.007322	0.031049
IL33	-2.135	-4.394	1.46E-06	3.31E-05
C15orf52	-2.135	-4.394	6.70E-06	0.000117
LOC100131564	-2.133	-4.385	7.30E-05	0.000812
NA	-2.132	-4.384	0.012068	0.045364
ZNF630	-2.131	-4.381	0.01141	0.043614
RNF182	-2.131	-4.38	0.002206	0.012294
LINC01024	-2.13	-4.377	0.004005	0.0194
RAD51-AS1	-2.129	-4.373	0.000219	0.002006
LOC101927204	-2.127	-4.368	0.008344	0.034385
SLC38A4	-2.126	-4.365	0.001054	0.006822
NAV2	-2.126	-4.364	2.94E-07	8.35E-06
FBN2	-2.125	-4.362	0.003113	0.015995
IL18BP	-2.125	-4.362	0.001492	0.009018
CREB3L1	-2.124	-4.36	0.000937	0.006224
SLIT3	-2.122	-4.354	2.01E-05	0.000288
NA	-2.121	-4.35	0.00677	0.029152
C3orf67	-2.12	-4.348	0.00226	0.012494
FANK1	-2.119	-4.345	0.000908	0.006073
NA	-2.118	-4.341	0.004969	0.022865

ENTPD7	-2.117	-4.339	9.87E-05	0.001043
DLGAP1-AS1	-2.116	-4.335	0.003421	0.017245
ACVR1	-2.114	-4.329	7.57E-07	1.91E-05
KIAA1683	-2.109	-4.314	0.005731	0.025666
NA	-2.108	-4.312	0.013432	0.049402
AVIL	-2.107	-4.307	0.000443	0.003495
MBOAT1	-2.106	-4.306	0.011922	0.045027
ZNF767P	-2.106	-4.304	7.35E-05	0.000815
EID3	-2.104	-4.3	0.00695	0.029733
AKT3	-2.101	-4.289	1.17E-05	0.000187
WNT5A	-2.097	-4.278	8.57E-05	0.000927
CXCL12	-2.095	-4.273	0.009212	0.037032
GALNT10	-2.095	-4.272	0.000116	0.001188
PCDHGC3	-2.093	-4.267	1.47E-06	3.33E-05
GPNMB	-2.092	-4.265	4.80E-05	0.000578
NKX3-1	-2.085	-4.244	0.001296	0.008047
ACRC	-2.082	-4.234	0.002982	0.0155
RAET1G	-2.079	-4.226	0.011683	0.044311
LOC101928524	-2.079	-4.226	0.0006	0.004405
FHDC1	-2.079	-4.224	0.008141	0.033691
NA	-2.078	-4.222	0.000988	0.006483
KLF5	-2.078	-4.222	0.004857	0.02245
HDGFRP3	-2.078	-4.221	1.29E-05	0.000204
LIMK1	-2.077	-4.219	5.52E-07	1.46E-05
ZFP36L1	-2.075	-4.213	0.000239	0.002141
PNMAL1	-2.074	-4.211	0.00086	0.005819
LRIG3	-2.073	-4.206	1.59E-06	3.54E-05
LRP4	-2.072	-4.204	7.79E-07	1.95E-05
PGM2L1	-2.069	-4.195	0.001449	0.008802
TMEM154	-2.068	-4.193	0.004333	0.020566
PDGFRB	-2.068	-4.192	0.00011	0.001144
INADL	-2.067	-4.191	5.00E-05	0.000597
FAM66C	-2.067	-4.189	0.004663	0.021729
ITGA1	-2.067	-4.189	0.000658	0.00476
GUSBP11	-2.066	-4.189	5.91E-06	0.000105
EPHA2	-2.066	-4.188	0.010152	0.039859
DSP	-2.066	-4.188	0.002765	0.01461
LOC202181	-2.065	-4.185	0.011643	0.044207
FBXO5	-2.065	-4.183	0.000887	0.005968
EPSTI1	-2.062	-4.177	0.001953	0.011168
TRIB3	-2.059	-4.166	0.005011	0.023014
SAMD9	-2.058	-4.165	3.98E-05	0.0005
BRCA2	-2.052	-4.147	0.013076	0.04834
NA	-2.052	-4.147	0.003636	0.01802
CCDC74A	-2.051	-4.143	0.003213	0.016398
A4GALT	-2.051	-4.143	3.97E-06	7.65E-05
DNAH7	-2.049	-4.139	0.011969	0.045118
MTCL1	-2.048	-4.137	0.000102	0.001073
PRDM8	-2.048	-4.135	0.000336	0.002799
JAK3	-2.046	-4.131	0.00115	0.007298

JDP2	-2.044	-4.123	0.000158	0.001548
COL4A5	-2.044	-4.123	0.002562	0.013789
LINC00894	-2.044	-4.123	0.001753	0.010262
SMAD3	-2.044	-4.123	1.23E-06	2.89E-05
SLC6A15	-2.041	-4.115	0.000965	0.00637
NA	-2.04	-4.111	0.000148	0.001467
KLF4	-2.039	-4.109	0.001668	0.009869
NT5E	-2.036	-4.101	0.000286	0.002486
LINC01133	-2.035	-4.099	0.001077	0.006934
TCF19	-2.034	-4.095	0.003846	0.01882
RORA	-2.034	-4.094	3.38E-05	0.000435
SRGAP1	-2.033	-4.093	2.46E-06	5.12E-05
RARG	-2.033	-4.091	0.000678	0.004856
NA	-2.026	-4.072	0.000873	0.005885
TBC1D8	-2.025	-4.069	5.58E-05	0.00065
C1S	-2.024	-4.068	0.000166	0.001607
GPR161	-2.024	-4.067	0.000535	0.004034
FAM101B	-2.023	-4.065	6.11E-06	0.000108
CNN1	-2.023	-4.063	0.001006	0.006572
CLEC14A	-2.02	-4.056	0.011939	0.045077
CPT1A	-2.02	-4.054	0.000485	0.003735
IRX1	-2.018	-4.049	0.00072	0.005101
MIR663AHG	-2.017	-4.048	0.009198	0.036989
ECM1	-2.017	-4.047	1.74E-06	3.81E-05
MYCBP2	-2.015	-4.041	0.001054	0.006822
MRVI1	-2.014	-4.039	0.001354	0.008341
NBPF10	-2.013	-4.037	0.000973	0.006407
VGLL3	-2.009	-4.026	0.000477	0.003681
CHEK2	-2.009	-4.025	0.00036	0.002958
LOC101927841	-2.007	-4.02	0.009352	0.037464
CTGF	-2.005	-4.013	0.000312	0.002646
PXN	-1.997	-3.991	0.000342	0.002833
MAP3K14	-1.996	-3.99	0.001677	0.009908
TPCN1	-1.996	-3.989	2.84E-06	5.78E-05
KCNK6	-1.996	-3.988	0.001677	0.009908
ADM	-1.994	-3.983	0.000834	0.005692
RNF213	-1.994	-3.983	4.19E-05	0.000521
LRRK2	-1.994	-3.982	2.56E-06	5.29E-05
ARMCX4	-1.993	-3.981	6.61E-05	0.000749
ZNF273	-1.989	-3.971	0.00913	0.036822
FSD1L	-1.988	-3.967	0.003234	0.01649
ZEB1	-1.985	-3.958	3.65E-05	0.000465
KCNMB4	-1.984	-3.955	0.011945	0.045089
LRRC37A4P	-1.983	-3.954	0.002469	0.013376
OAS3	-1.982	-3.95	0.000489	0.003746
GOLGA8B	-1.982	-3.95	0.0002	0.001872
CRIM1	-1.978	-3.939	0.000453	0.003547
TDRD7	-1.977	-3.936	0.000307	0.002616
STMN3	-1.976	-3.934	0.005269	0.023962
PTCHD4	-1.976	-3.933	0.002202	0.012279

TBX19	-1.975	-3.93	0.001297	0.008047
SULF2	-1.975	-3.93	4.09E-05	0.00051
ANKRD28	-1.971	-3.922	8.82E-05	0.000948
NA	-1.971	-3.921	0.011503	0.043861
NR4A2	-1.968	-3.914	0.007176	0.030535
PLEC	-1.96	-3.89	0.000402	0.003242
IGF1R	-1.958	-3.886	1.04E-05	0.00017
HIC1	-1.957	-3.882	0.000386	0.003138
PLSCR4	-1.957	-3.882	7.11E-05	0.000795
SDC4	-1.952	-3.87	1.20E-05	0.00019
CENPE	-1.951	-3.866	0.004808	0.022291
TNS3	-1.951	-3.865	0.005026	0.023061
PTK7	-1.95	-3.865	0.000111	0.001149
FLNA	-1.95	-3.863	0.000303	0.002593
IFT81	-1.948	-3.858	0.001988	0.011345
WIPF1	-1.947	-3.856	2.81E-05	0.000379
HCLS1	-1.944	-3.847	0.000433	0.003445
LOXL3	-1.943	-3.846	5.26E-05	0.000622
HOXD4	-1.942	-3.843	0.000821	0.005619
TRIM59	-1.94	-3.836	0.012034	0.045264
SPHK1	-1.938	-3.831	0.000445	0.003505
UBAP1L	-1.932	-3.817	0.003545	0.017676
HYPK	-1.932	-3.816	0.005378	0.02437
TMEM87B	-1.928	-3.805	3.79E-05	0.00048
TFPI	-1.927	-3.802	2.60E-05	0.000357
ANKRD34A	-1.923	-3.792	0.006504	0.028212
HERC2P9	-1.923	-3.791	0.000621	0.004533
PPAP2B	-1.92	-3.785	0.00031	0.002636
ZFP37	-1.92	-3.784	0.001644	0.009761
LINC00607	-1.918	-3.78	0.003963	0.019248
FAM198A	-1.918	-3.779	0.009568	0.03818
LPXN	-1.917	-3.777	0.003287	0.016702
ERCC6	-1.914	-3.769	0.001808	0.010499
MMP11	-1.914	-3.768	0.006147	0.027017
SGK1	-1.913	-3.767	7.01E-05	0.000786
WDR54	-1.913	-3.765	0.001615	0.009622
BGN	-1.906	-3.749	0.000574	0.004259
OSBPL10	-1.906	-3.748	0.000901	0.006044
ANKHD1	-1.906	-3.748	0.012381	0.046338
STXBP5	-1.902	-3.738	0.006711	0.028974
RCAN1	-1.902	-3.737	8.07E-05	0.00088
MITF	-1.902	-3.736	7.41E-05	0.000821
PCDHB4	-1.899	-3.73	0.01267	0.047171
CAPS	-1.897	-3.724	0.000943	0.006258
LOXL4	-1.897	-3.723	0.003269	0.016637
DDX60L	-1.894	-3.716	2.75E-05	0.000371
CAMK1D	-1.892	-3.712	0.000707	0.005025
SPON1	-1.889	-3.703	0.000279	0.002433
DOCK9	-1.887	-3.699	7.99E-05	0.000874
NA	-1.886	-3.697	0.008216	0.033942

TCF4	-1.886	-3.696	8.73E-05	0.000939
IL18R1	-1.886	-3.695	0.006096	0.026848
TMEM44	-1.886	-3.695	0.004226	0.020141
LPCAT4	-1.885	-3.694	0.000793	0.005481
SAMD9L	-1.885	-3.693	0.002301	0.01263
LIMS2	-1.884	-3.691	0.00137	0.008427
MFAP4	-1.881	-3.683	0.000446	0.003505
CD9	-1.88	-3.68	1.31E-05	0.000207
NA	-1.879	-3.677	0.0129	0.04783
RPGRIP1L	-1.879	-3.677	5.58E-05	0.00065
NA	-1.877	-3.673	0.002333	0.012765
IGF1	-1.876	-3.67	0.007964	0.033182
PDLIM7	-1.874	-3.666	0.000155	0.001523
CIDEB	-1.871	-3.658	0.008029	0.033395
SSC5D	-1.87	-3.656	0.000117	0.001199
NA	-1.87	-3.656	0.006483	0.028157
HLA-C	-1.87	-3.655	3.35E-05	0.000432
CPM	-1.869	-3.653	0.009713	0.038613
DMD	-1.869	-3.652	0.000209	0.001934
LOC729603	-1.867	-3.648	0.007137	0.030399
NHSL2	-1.866	-3.646	0.006089	0.026832
RHOJ	-1.865	-3.642	0.000592	0.00436
LOC155060	-1.865	-3.642	0.004681	0.021778
CRYGS	-1.865	-3.642	0.007629	0.032057
HTRA1	-1.864	-3.641	0.000548	0.004102
FAM109B	-1.864	-3.639	4.06E-05	0.000508
PCDHGB2	-1.862	-3.636	0.001004	0.006565
RP9P	-1.862	-3.636	0.003491	0.017502
MFI2	-1.859	-3.627	0.000115	0.00118
SCD5	-1.857	-3.623	2.36E-05	0.000329
HAND2	-1.857	-3.622	2.13E-05	0.000303
CCDC84	-1.854	-3.616	9.99E-05	0.001053
DNALI1	-1.854	-3.615	0.001297	0.008047
IGFBP4	-1.853	-3.612	0.000384	0.003131
MEIS3	-1.851	-3.608	1.11E-05	0.000179
EFCAB7	-1.851	-3.608	0.000846	0.005761
C1R	-1.848	-3.599	0.001739	0.010199
TMPO	-1.846	-3.595	0.001771	0.010355
MT1E	-1.841	-3.583	0.000903	0.006049
TNFRSF19	-1.841	-3.583	0.013034	0.048225
HS3ST3B1	-1.841	-3.582	0.001359	0.008367
TGFBR2	-1.84	-3.581	0.000413	0.003315
AHSA2	-1.839	-3.577	5.98E-05	0.000689
PEAK1	-1.837	-3.572	0.000836	0.005706
NFYB	-1.834	-3.566	4.70E-05	0.000568
JAM3	-1.834	-3.565	1.34E-05	0.00021
CFAP44	-1.833	-3.563	0.000853	0.005784
TGFB3	-1.833	-3.562	0.002247	0.01246
LINC00926	-1.832	-3.561	0.011477	0.043808
DOK4	-1.828	-3.551	3.10E-05	0.000406

GRK5	-1.827	-3.547	1.35E-05	0.000212
CDH24	-1.824	-3.541	0.002869	0.015052
RIN2	-1.824	-3.54	1.36E-05	0.000213
HLA-E	-1.824	-3.54	9.59E-05	0.00102
SH3RF3	-1.823	-3.538	1.67E-05	0.00025
DDAH1	-1.822	-3.536	0.001439	0.008754
SLC6A6	-1.821	-3.534	4.43E-06	8.36E-05
SDHAP1	-1.821	-3.532	0.003097	0.015941
NTNG2	-1.819	-3.529	0.011714	0.044403
TCP11L2	-1.818	-3.525	0.000127	0.001282
KIAA1024	-1.817	-3.524	0.001919	0.010997
PCNXL2	-1.817	-3.523	0.001733	0.010168
PDLIM2	-1.815	-3.519	5.20E-05	0.000616
EVC2	-1.811	-3.51	0.001004	0.006565
CEP162	-1.811	-3.509	0.001749	0.010247
C8orf88	-1.811	-3.508	0.003022	0.015658
RABL2A	-1.81	-3.507	0.000832	0.00568
PBX3	-1.81	-3.507	2.47E-05	0.00034
B3GALT4	-1.81	-3.505	0.00454	0.021298
NPR3	-1.807	-3.5	0.000111	0.001156
APOL3	-1.807	-3.498	5.07E-05	0.000604
TP53I11	-1.807	-3.498	0.001828	0.01058
CNTNAP1	-1.806	-3.496	1.03E-05	0.000168
ZFHX4	-1.805	-3.494	0.000336	0.002799
APOBEC3F	-1.802	-3.488	0.009223	0.037065
HNMT	-1.802	-3.488	0.000104	0.001095
TPBG	-1.801	-3.485	0.000487	0.003739
TIMP1	-1.801	-3.485	0.004982	0.022896
TRAM2	-1.8	-3.483	0.00066	0.004768
FHL3	-1.798	-3.478	0.000571	0.004247
LOC101929038	-1.798	-3.478	0.003753	0.018469
NA	-1.798	-3.477	0.008064	0.033467
RAPH1	-1.797	-3.474	5.96E-05	0.000688
NEXN	-1.793	-3.466	0.003033	0.015701
ABL2	-1.791	-3.462	0.000188	0.001777
SCART1	-1.791	-3.461	0.009966	0.039377
RRN3P3	-1.789	-3.456	0.013286	0.048957
FTX	-1.788	-3.453	0.006901	0.029578
TACC1	-1.787	-3.451	0.000853	0.005784
LAMA1	-1.785	-3.445	0.012782	0.047484
KIAA0754	-1.782	-3.439	0.000487	0.003739
WEE1	-1.781	-3.437	0.000291	0.00251
COLGALT2	-1.781	-3.437	0.000886	0.005965
KIAA1407	-1.781	-3.436	0.008036	0.033395
PLEKHH2	-1.78	-3.435	0.001206	0.007565
NPIPB15	-1.78	-3.434	0.007805	0.032609
S1PR2	-1.78	-3.434	0.002759	0.014585
GLS	-1.78	-3.434	0.001814	0.01052
SNHG23	-1.779	-3.432	0.002013	0.011444
HMGA2	-1.779	-3.431	0.005783	0.025823

LOC100130950	-1.778	-3.429	0.012513	0.046707
SLIT2	-1.778	-3.428	0.000942	0.006258
FEZ1	-1.777	-3.427	3.32E-06	6.57E-05
ANO4	-1.776	-3.425	0.001621	0.009647
FIGF	-1.776	-3.425	0.008704	0.035506
SERPING1	-1.776	-3.424	0.002658	0.014181
ANTXR2	-1.776	-3.424	0.001237	0.007734
NA	-1.775	-3.423	0.004339	0.020588
BACH1	-1.775	-3.421	1.90E-05	0.000277
NBPF14	-1.773	-3.417	0.002007	0.011425
TMEM67	-1.772	-3.415	0.000695	0.004952
SAMD4A	-1.77	-3.41	0.000201	0.001873
TNFRSF12A	-1.77	-3.41	0.005962	0.026395
LINC01116	-1.769	-3.407	0.003706	0.018288
CD44	-1.767	-3.404	0.000539	0.004055
ELK3	-1.765	-3.398	2.99E-05	0.000395
RTN2	-1.764	-3.397	0.000337	0.002806
FAM229A	-1.764	-3.395	0.006095	0.026848
ANLN	-1.762	-3.393	0.006605	0.02858
C16orf45	-1.761	-3.39	3.98E-05	0.0005
SBF2-AS1	-1.761	-3.389	0.005395	0.02443
TSPAN5	-1.759	-3.385	0.000495	0.003784
IGFBP6	-1.758	-3.383	0.004888	0.02257
RWDD2A	-1.757	-3.379	0.001537	0.009253
ATG16L2	-1.757	-3.379	0.008911	0.03609
NA	-1.754	-3.374	0.00997	0.039384
FOXL1	-1.753	-3.371	0.007766	0.032487
MACF1	-1.751	-3.367	0.000687	0.004904
BACE2	-1.751	-3.366	1.81E-05	0.000267
SNAI2	-1.748	-3.359	9.45E-05	0.001008
IFRD1	-1.743	-3.347	0.000253	0.002238
CCDC57	-1.743	-3.347	0.000178	0.001701
AMPD3	-1.742	-3.345	0.000425	0.003389
SFT2D2	-1.742	-3.344	0.001797	0.010457
OFD1	-1.74	-3.34	0.00052	0.00394
HOXB3	-1.738	-3.336	0.000593	0.004365
AP1G2	-1.738	-3.335	0.004634	0.021634
MAP3K8	-1.737	-3.334	0.00224	0.012439
PLP2	-1.736	-3.332	1.70E-05	0.000252
UPP1	-1.736	-3.332	0.002393	0.013044
EFEMP2	-1.734	-3.326	0.000244	0.002181
HOXD3	-1.733	-3.324	0.00085	0.005775
FHOD3	-1.732	-3.322	0.003618	0.017962
ZBTB20	-1.728	-3.313	0.006007	0.026552
PKM	-1.728	-3.313	0.000454	0.003548
CRIP2	-1.726	-3.309	0.000767	0.005343
HEPH	-1.726	-3.309	0.000813	0.005589
LOC100507053	-1.724	-3.303	0.001774	0.010363
C1orf21	-1.724	-3.303	6.27E-05	0.000715
NOTCH2NL	-1.722	-3.299	5.14E-05	0.000611

ABTB1	-1.721	-3.298	0.000341	0.002832
C8orf58	-1.721	-3.296	0.002275	0.012565
ARRDC2	-1.72	-3.294	0.000872	0.005885
AGAP6	-1.719	-3.292	0.002006	0.011424
FOXO3	-1.719	-3.291	0.003994	0.019376
EMX2	-1.719	-3.291	0.000703	0.005007
CASC10	-1.717	-3.287	0.010305	0.040364
MYO1E	-1.716	-3.286	0.000484	0.00373
SH3BP5-AS1	-1.716	-3.286	0.001804	0.010482
IFIT2	-1.716	-3.286	0.00085	0.005775
PARP12	-1.716	-3.285	0.001553	0.009314
ACVRL1	-1.716	-3.285	1.46E-05	0.000226
DTX3	-1.715	-3.284	0.004429	0.020926
GPSM2	-1.714	-3.28	0.005026	0.023061
CACNA1A	-1.712	-3.276	0.005118	0.023441
RBM43	-1.71	-3.272	0.000346	0.002859
CBLB	-1.709	-3.268	2.62E-05	0.000358
C2orf81	-1.705	-3.261	0.012698	0.04725
ZNF432	-1.703	-3.255	0.000224	0.002037
KLF7	-1.701	-3.251	7.01E-05	0.000786
SKIL	-1.701	-3.25	0.000482	0.003713
COL6A3	-1.699	-3.247	0.000909	0.006074
DGKI	-1.699	-3.247	0.00812	0.033627
MVP	-1.699	-3.246	0.000198	0.001855
ATP8B4	-1.695	-3.238	0.003403	0.017177
ALDH1A3	-1.695	-3.237	0.000763	0.005326
CLK1	-1.695	-3.237	1.74E-05	0.000258
ZNF112	-1.694	-3.236	0.002033	0.011514
ARHGEF10	-1.694	-3.235	3.11E-05	0.000407
NA	-1.693	-3.234	0.007426	0.031401
GJA1	-1.691	-3.228	0.001059	0.006839
GOLGA6L5P	-1.691	-3.228	0.004747	0.022031
SNX7	-1.689	-3.224	0.000181	0.001728
FXYD5	-1.688	-3.222	0.000909	0.006074
FZD6	-1.687	-3.221	0.002027	0.011494
AMMECR1	-1.687	-3.219	0.00173	0.010155
RALGPS2	-1.686	-3.218	0.002526	0.013631
IFIT3	-1.685	-3.215	8.54E-05	0.000925
LOC101928623	-1.684	-3.214	0.000512	0.00389
ALDH1L2	-1.679	-3.203	0.005758	0.025758
AR	-1.679	-3.203	0.000189	0.001786
SORBS2	-1.679	-3.203	0.000341	0.002829
IL1RAP	-1.679	-3.201	0.000377	0.003082
PAWR	-1.678	-3.2	0.002109	0.011862
CYB5R2	-1.678	-3.199	0.002721	0.01446
LOC100132077	-1.678	-3.199	0.01098	0.042327
TGFB1I1	-1.676	-3.196	0.000288	0.002498
NKAPL	-1.673	-3.189	0.003721	0.018354
ADARB1	-1.672	-3.187	0.01205	0.045312
LAMC1	-1.671	-3.185	0.001704	0.01003

HECTD2	-1.668	-3.177	0.000317	0.00268
HAND2-AS1	-1.667	-3.176	0.000814	0.005594
DNAJB4	-1.667	-3.175	0.000475	0.003666
KDM3A	-1.665	-3.171	1.95E-05	0.000282
YDJC	-1.664	-3.169	0.00687	0.029489
PPP1R3B	-1.664	-3.168	0.000214	0.001973
ATP8B1	-1.663	-3.168	0.006467	0.028108
LOC100996732	-1.662	-3.164	0.006827	0.029352
NAAA	-1.66	-3.16	0.000958	0.006329
PCDHGA12	-1.659	-3.158	4.54E-05	0.000554
CYB561	-1.658	-3.155	0.000234	0.002109
WDR27	-1.656	-3.152	0.000678	0.004856
A1BG-AS1	-1.656	-3.152	0.003405	0.017177
CTSO	-1.655	-3.149	4.45E-05	0.000547
TNFAIP2	-1.654	-3.147	0.002696	0.014361
NA	-1.651	-3.14	0.009978	0.039396
KMT2A	-1.651	-3.14	0.000119	0.001218
ZNF418	-1.651	-3.14	0.004378	0.020737
ADAM10	-1.65	-3.139	0.001548	0.009299
AJUBA	-1.65	-3.139	0.006467	0.028108
DEK	-1.648	-3.133	0.00091	0.006078
NA	-1.647	-3.132	0.000672	0.004831
IL16	-1.646	-3.13	0.000929	0.006179
NA	-1.646	-3.13	0.000292	0.002517
PTPLAD2	-1.645	-3.127	0.001593	0.009526
ASAP2	-1.643	-3.123	0.000274	0.002395
SESN3	-1.643	-3.123	0.00123	0.007696
FLRT3	-1.641	-3.12	0.007348	0.031132
B4GALT4	-1.641	-3.119	0.000246	0.002188
NA	-1.64	-3.117	0.000147	0.001461
TMEM159	-1.639	-3.115	0.000314	0.002658
TRIP10	-1.636	-3.108	7.30E-05	0.000812
GFRA1	-1.636	-3.108	0.000386	0.003137
KIZ	-1.636	-3.108	0.000497	0.003789
RAI14	-1.634	-3.104	0.003269	0.016637
LYST	-1.633	-3.101	2.37E-05	0.00033
DMTF1	-1.633	-3.101	2.63E-05	0.000359
NKTR	-1.631	-3.098	9.60E-05	0.001021
GABARAPL1	-1.631	-3.097	0.000289	0.002498
SEC31B	-1.629	-3.092	0.000949	0.006287
PNISR	-1.628	-3.09	0.000166	0.001604
SLC16A3	-1.626	-3.087	0.000962	0.006349
SH3PXD2B	-1.626	-3.086	0.001106	0.007068
CNPY4	-1.626	-3.086	0.000258	0.002275
LOC440300	-1.625	-3.084	0.013448	0.049437
PKD2	-1.624	-3.083	0.00136	0.008367
TAGLN	-1.623	-3.081	0.003484	0.017489
CYTH3	-1.62	-3.075	0.001891	0.01089
CCDC71L	-1.619	-3.071	0.002518	0.013598
PTCH1	-1.618	-3.07	0.0003	0.002567

KIAA1109	-1.618	-3.07	0.000312	0.002646
FAM71F2	-1.616	-3.065	0.011867	0.04488
SLC7A11	-1.614	-3.062	0.010752	0.04166
C7orf60	-1.614	-3.06	0.003465	0.017431
DDIT3	-1.613	-3.059	0.001234	0.007724
MBNL2	-1.613	-3.058	0.002126	0.011932
TPST1	-1.612	-3.057	6.42E-05	0.00073
OMD	-1.612	-3.057	0.0002	0.001872
RAB27A	-1.611	-3.056	0.001014	0.006618
KCNAB2	-1.611	-3.055	0.002754	0.014575
SMAD7	-1.61	-3.052	0.000395	0.003199
LINC00152	-1.608	-3.049	0.000842	0.005737
STX2	-1.608	-3.048	0.000133	0.001334
B4GALT6	-1.601	-3.035	0.002996	0.015554
ZNF251	-1.601	-3.034	0.000692	0.004938
PPFIBP2	-1.598	-3.026	0.000212	0.00196
ZNF300	-1.597	-3.024	0.001979	0.011304
GLT8D1	-1.593	-3.017	9.72E-05	0.00103
SYNGAP1	-1.593	-3.016	0.001199	0.007537
TRIB2	-1.593	-3.016	0.000802	0.005527
FOXP1	-1.59	-3.011	0.000257	0.002267
ARHGAP6	-1.59	-3.011	0.008037	0.033395
TCIRG1	-1.589	-3.008	0.000759	0.005304
SIRPA	-1.589	-3.008	0.000948	0.006287
NID2	-1.588	-3.005	0.007691	0.032261
NLRC5	-1.587	-3.004	0.000248	0.002205
IRX2	-1.582	-2.994	0.000485	0.003734
UBE2L6	-1.581	-2.992	3.34E-05	0.000432
NPIPA5	-1.58	-2.989	0.000403	0.003248
CDC42EP3	-1.578	-2.985	0.002015	0.011447
SFI1	-1.576	-2.981	0.006034	0.026651
PHC1	-1.574	-2.978	0.000463	0.003596
HAPLN3	-1.572	-2.973	0.001122	0.007157
GYPE	-1.571	-2.97	0.006923	0.029656
MAGI3	-1.569	-2.967	0.000562	0.004192
FBXL7	-1.561	-2.95	0.000121	0.001235
B4GALT1	-1.561	-2.95	0.004424	0.020918
GSTM3	-1.558	-2.944	0.000128	0.001294
FLJ31306	-1.556	-2.941	0.000235	0.002114
KIAA1614	-1.555	-2.939	0.002138	0.011977
LPCAT1	-1.555	-2.938	0.000364	0.002988
ZNF608	-1.552	-2.933	0.001187	0.007479
LHFP	-1.55	-2.927	0.000473	0.00366
GNB4	-1.548	-2.924	0.001917	0.010991
LMOD1	-1.546	-2.921	0.011488	0.043837
ARL13B	-1.545	-2.918	0.001434	0.008731
IPMK	-1.543	-2.915	0.001824	0.010563
VEGFC	-1.543	-2.914	0.000444	0.003498
NA	-1.54	-2.908	0.001928	0.011047
GDAP1	-1.537	-2.902	0.007486	0.031578

C10orf54	-1.537	-2.901	4.95E-05	0.000595
TTC7A	-1.536	-2.901	0.000356	0.002931
CD99P1	-1.534	-2.895	0.011658	0.04423
GPRASP1	-1.533	-2.895	0.006164	0.027076
NA	-1.533	-2.894	0.000859	0.005812
ZSCAN30	-1.532	-2.893	0.000222	0.002021
FKBP10	-1.532	-2.892	0.001353	0.008338
SPTLC3	-1.532	-2.892	0.005424	0.024523
RASSF8	-1.532	-2.891	0.001103	0.00706
STAT6	-1.53	-2.887	0.001046	0.006781
SLC13A3	-1.528	-2.885	0.00561	0.025236
CD27-AS1	-1.525	-2.878	0.001634	0.009713
OSBPL5	-1.525	-2.877	7.28E-05	0.000811
C19orf66	-1.524	-2.877	0.000754	0.005281
UST	-1.524	-2.876	0.003376	0.017074
NXPE3	-1.52	-2.868	0.000229	0.002076
RASAL2	-1.519	-2.866	0.00133	0.008219
POLR2J4	-1.519	-2.865	0.012625	0.04703
GAL3ST4	-1.517	-2.862	0.00885	0.035929
STAG1	-1.516	-2.86	0.000192	0.001812
REV3L	-1.514	-2.857	0.012787	0.047491
TMOD2	-1.514	-2.856	0.00047	0.003643
CTSF	-1.514	-2.855	0.000109	0.001136
LOC100506634	-1.511	-2.85	0.002905	0.015183
BDH2	-1.511	-2.85	0.00027	0.002366
IMPA2	-1.51	-2.849	0.001512	0.009128
SMURF2	-1.509	-2.846	0.004105	0.019755
ARRDC4	-1.509	-2.846	0.000436	0.00346
SLC15A3	-1.507	-2.842	0.003907	0.019071
MIR99AHG	-1.506	-2.84	0.011545	0.043991
FAS	-1.504	-2.836	0.000141	0.001404
ZNHIT6	-1.503	-2.834	0.00148	0.008963
STAMBPL1	-1.502	-2.832	0.003504	0.017548
BEND6	-1.5	-2.828	0.007415	0.031376
MGAT5	-1.498	-2.825	0.003274	0.016651
SFXN3	-1.496	-2.821	0.000167	0.001615
LRRC37A3	-1.495	-2.819	0.013003	0.048121
CTSB	-1.494	-2.817	0.005197	0.023749
SLC38A2	-1.494	-2.817	0.004715	0.021905
SH3BP4	-1.493	-2.816	0.001693	0.009984
TTC26	-1.492	-2.813	0.005234	0.02385
GLIS2	-1.492	-2.813	0.007086	0.030246
IFI35	-1.49	-2.809	0.003906	0.019071
GOLIM4	-1.488	-2.805	0.000612	0.004477
NPIPA5	-1.488	-2.805	0.00044	0.003487
MAP1B	-1.488	-2.804	0.003706	0.018288
JADE1	-1.487	-2.804	0.00624	0.027348
ARID5B	-1.487	-2.803	0.003741	0.018442
KDELC1	-1.487	-2.803	0.001119	0.00714
FES	-1.487	-2.803	0.008205	0.033905

SEC24D	-1.485	-2.8	0.003037	0.015701
SNAPC1	-1.484	-2.798	0.010409	0.040697
ADCY9	-1.484	-2.797	0.000158	0.001546
MICAL1	-1.483	-2.796	0.000178	0.001708
GLI3	-1.483	-2.795	0.000257	0.002266
LEPREL2	-1.482	-2.794	0.001537	0.009253
ZC2HC1A	-1.482	-2.794	0.007159	0.030482
CARD6	-1.482	-2.793	5.81E-05	0.000672
CARHSP1	-1.481	-2.792	0.00023	0.002076
B3GALTL	-1.481	-2.79	0.000727	0.005145
MSANTD3	-1.48	-2.79	0.003815	0.018714
SPRED2	-1.48	-2.789	0.000279	0.002433
GALNT7	-1.476	-2.782	0.003775	0.018551
MIR4435-1HG	-1.476	-2.781	0.002587	0.013901
NFATC1	-1.476	-2.781	0.006678	0.028868
ACCS	-1.475	-2.781	0.005796	0.025852
ST3GAL5	-1.474	-2.779	0.000399	0.003225
ABCC5	-1.473	-2.775	0.00042	0.003361
AP3M2	-1.472	-2.775	0.002116	0.011882
ADAM9	-1.471	-2.772	0.001944	0.011127
DOCK5	-1.469	-2.769	0.000629	0.004585
FBLN2	-1.467	-2.765	0.004903	0.022618
NEAT1	-1.467	-2.764	0.003402	0.017177
CEBPD	-1.466	-2.763	0.00391	0.019071
NA	-1.463	-2.756	0.001519	0.009166
FOSL2	-1.463	-2.756	0.002942	0.015345
MPZL1	-1.461	-2.753	0.003774	0.018551
CHD3	-1.46	-2.752	0.00152	0.009166
PSIP1	-1.459	-2.749	0.003336	0.016921
SOCS5	-1.459	-2.748	0.000829	0.005664
IDS	-1.457	-2.746	0.000508	0.003864
ID2	-1.456	-2.744	0.000767	0.005343
RAB11FIP2	-1.456	-2.743	0.002789	0.014729
NAV3	-1.455	-2.742	0.001745	0.010228
RNF19A	-1.455	-2.742	0.001892	0.01089
OGT	-1.455	-2.741	0.003996	0.019378
SH3D19	-1.449	-2.731	0.002573	0.013842
THNSL2	-1.448	-2.729	0.000633	0.004602
ZNF503	-1.446	-2.724	0.000384	0.003131
MYSM1	-1.444	-2.721	0.000233	0.002099
MTHFD2	-1.441	-2.715	0.00923	0.037071
NBPF1	-1.439	-2.712	0.00016	0.001557
TNFAIP8L3	-1.436	-2.706	0.007271	0.030874
HTR7P1	-1.436	-2.706	0.004945	0.022771
ADAMTS5	-1.436	-2.705	0.005886	0.026134
CHIC2	-1.433	-2.7	0.003929	0.019129
KCTD17	-1.433	-2.699	0.009147	0.036879
KAZN	-1.431	-2.696	0.000767	0.005345
ZNF107	-1.428	-2.69	0.011289	0.043312
ZNF415	-1.427	-2.689	0.009016	0.036429

PARD3B	-1.426	-2.688	0.001048	0.006791
GEM	-1.424	-2.684	0.011558	0.043994
MLH3	-1.422	-2.68	0.000758	0.0053
FAM76B	-1.42	-2.675	0.004466	0.021045
RNF145	-1.419	-2.674	0.000819	0.005614
ZNF700	-1.418	-2.672	0.008029	0.033395
LOC653160	-1.417	-2.671	0.011385	0.043569
UBE2Q2P1	-1.416	-2.669	0.01218	0.04571
SGCE	-1.416	-2.668	0.000196	0.001843
C14orf37	-1.415	-2.667	0.002222	0.012363
MIR3916	-1.414	-2.665	0.005792	0.02585
PCDHGA9	-1.414	-2.665	0.010621	0.041282
APBB2	-1.406	-2.649	0.002512	0.013569
GORAB	-1.405	-2.649	0.005049	0.023151
ZNF8	-1.404	-2.647	0.007905	0.032996
SLC26A2	-1.404	-2.647	0.001152	0.007304
N4BP2L2	-1.403	-2.644	0.000486	0.003739
MFAP3L	-1.401	-2.642	0.005263	0.023946
LPCAT2	-1.401	-2.64	0.010537	0.041049
NEK3	-1.398	-2.635	0.007244	0.030785
LCAT	-1.398	-2.635	0.009762	0.038738
SLC44A2	-1.396	-2.632	0.002631	0.014079
ENG	-1.395	-2.629	0.003286	0.016702
WDFY2	-1.394	-2.627	0.010887	0.042039
SLC39A14	-1.393	-2.626	0.002969	0.015449
GCC2	-1.393	-2.626	0.002157	0.012072
ALDH3B1	-1.393	-2.626	0.001944	0.011127
PSMD5-AS1	-1.393	-2.626	0.004448	0.020996
CPE	-1.393	-2.626	0.00125	0.007801
CCNL1	-1.389	-2.619	0.002281	0.012587
C5orf45	-1.388	-2.617	0.004	0.019386
PIBF1	-1.387	-2.615	0.002717	0.014443
NCOA3	-1.386	-2.613	0.007613	0.032023
P4HA2	-1.385	-2.611	0.003019	0.01565
LPP	-1.381	-2.605	0.006632	0.028681
THUMPD3-AS1	-1.38	-2.603	0.004897	0.022597
ZFC3H1	-1.38	-2.603	0.001062	0.006858
SLC39A13	-1.379	-2.602	0.001896	0.010904
RGMA	-1.379	-2.601	0.002717	0.014443
PLEKHF1	-1.378	-2.6	0.003189	0.016302
TMEM50A	-1.377	-2.598	0.002705	0.014395
MAMLD1	-1.377	-2.597	0.001842	0.010644
NIPAL2	-1.376	-2.596	0.001753	0.010263
SLC4A7	-1.375	-2.594	0.003642	0.01804
IFFO1	-1.374	-2.592	0.010329	0.040435
GPCPD1	-1.371	-2.586	0.000685	0.004895
CYP2U1	-1.37	-2.585	0.000327	0.002748
TRAF1	-1.368	-2.582	0.003372	0.017068
TMEM43	-1.368	-2.582	0.002893	0.015143
VEZF1	-1.363	-2.573	0.000395	0.003199

ENTPD4	-1.363	-2.573	0.00174	0.010202
HIF1A	-1.363	-2.573	0.009956	0.03935
HDAC7	-1.362	-2.571	0.003404	0.017177
ARHGAP19	-1.362	-2.57	0.011631	0.044187
SMC5	-1.361	-2.568	0.001097	0.007032
LINC01089	-1.359	-2.566	0.008034	0.033395
HLA-A	-1.358	-2.564	0.003051	0.015761
SLC31A2	-1.358	-2.564	0.000739	0.005196
INSIG2	-1.358	-2.563	0.00229	0.01261
CTTN	-1.357	-2.562	0.005358	0.024289
CREBRF	-1.356	-2.56	0.000855	0.00579
PDIA5	-1.355	-2.558	0.000489	0.003749
PTPN21	-1.354	-2.557	0.000346	0.00286
FAXDC2	-1.354	-2.556	0.0008	0.005522
WIPI1	-1.354	-2.556	0.002013	0.011444
KIAA0753	-1.352	-2.553	0.003565	0.017757
CROCCP2	-1.352	-2.552	0.011564	0.044005
SMG1	-1.352	-2.552	0.003664	0.018122
OSR1	-1.351	-2.551	0.002608	0.01399
TPT1-AS1	-1.35	-2.55	0.012603	0.046962
LRRK1	-1.349	-2.547	0.000287	0.002487
SACS	-1.348	-2.545	0.002954	0.015385
C5orf28	-1.346	-2.541	0.003636	0.01802
NR1D2	-1.345	-2.54	0.008495	0.034843
TBC1D2	-1.344	-2.539	0.010254	0.040202
NT5C2	-1.341	-2.534	0.001331	0.008223
CTTNBP2NL	-1.339	-2.529	0.003515	0.017572
AKAP13	-1.338	-2.528	0.005663	0.025418
ABCC1	-1.337	-2.526	0.002796	0.014754
MYH9	-1.334	-2.522	0.012837	0.04765
VPS13B	-1.332	-2.518	0.001799	0.010459
WSB1	-1.332	-2.517	0.003487	0.017496
FGF5	-1.332	-2.517	0.004689	0.021809
BRWD3	-1.331	-2.516	0.005513	0.024851
LINC00654	-1.33	-2.513	0.001768	0.010345
SLC12A6	-1.329	-2.513	0.004691	0.021811
MAFB	-1.327	-2.509	0.004031	0.019485
SYBU	-1.326	-2.507	0.003049	0.015757
OSBPL9	-1.326	-2.506	0.004576	0.021421
LTBP3	-1.325	-2.505	0.003489	0.0175
FILIP1L	-1.32	-2.496	0.000732	0.005164
UNC93B1	-1.319	-2.495	0.00875	0.035618
CRYBB2P1	-1.319	-2.494	0.00981	0.038893
PLK2	-1.318	-2.493	0.002313	0.012683
RAB31	-1.317	-2.492	0.007765	0.032487
LENG8	-1.316	-2.49	0.009882	0.039127
RUFY3	-1.314	-2.487	0.001157	0.007331
KLF12	-1.313	-2.485	0.00449	0.021128
MYO10	-1.313	-2.484	0.004125	0.019809
RHOBTB1	-1.313	-2.484	0.010723	0.041571

WNT5B	-1.312	-2.483	0.012143	0.045604
PRKG1	-1.312	-2.482	0.001108	0.007079
PCSK5	-1.31	-2.48	0.013293	0.048971
CTDSP2	-1.31	-2.48	0.005659	0.025406
ATXN1	-1.31	-2.479	0.003521	0.017585
DNAJC10	-1.309	-2.477	0.007247	0.03079
TUSC3	-1.307	-2.475	0.002209	0.012305
CHIC1	-1.307	-2.474	0.00895	0.036217
AHR	-1.306	-2.473	0.004754	0.022058
CEP164	-1.306	-2.472	0.000772	0.005371
DZIP1	-1.302	-2.466	0.00225	0.012468
ARGLU1	-1.302	-2.465	0.00137	0.008427
ZNF337	-1.301	-2.464	0.001466	0.00889
FSCN1	-1.3	-2.463	0.008455	0.034708
GPX8	-1.3	-2.463	0.012514	0.046707
P4HTM	-1.298	-2.459	0.002614	0.014014
ZNF37A	-1.298	-2.458	0.000688	0.004908
CSNK1A1	-1.297	-2.458	0.00411	0.01976
EPG5	-1.296	-2.455	0.003033	0.015701
CCNL2	-1.293	-2.451	0.003871	0.018915
RHBDF1	-1.291	-2.447	0.005584	0.025128
NAPB	-1.291	-2.447	0.010505	0.040959
ARHGAP21	-1.291	-2.446	0.008937	0.036175
SEC14L1	-1.289	-2.443	0.00183	0.010586
C12orf57	-1.288	-2.442	0.000578	0.004281
SP100	-1.288	-2.442	0.001127	0.007185
FAM63B	-1.288	-2.442	0.008171	0.03379
PLEKHA5	-1.287	-2.441	0.001074	0.006921
HERPUD2	-1.287	-2.44	0.000753	0.005273
NPIPB5	-1.285	-2.436	0.00966	0.038469
THY1	-1.284	-2.435	0.008901	0.036061
SQRDL	-1.283	-2.433	0.001909	0.010962
ZNF441	-1.282	-2.431	0.008972	0.036284
C5orf42	-1.281	-2.43	0.000795	0.00549
TLDC1	-1.281	-2.43	0.01155	0.043991
EIF5A2	-1.281	-2.429	0.005231	0.023845
CDC25B	-1.28	-2.429	0.004518	0.021228
PXDC1	-1.28	-2.428	0.002739	0.014519
BOD1L1	-1.278	-2.425	0.004009	0.019414
BST1	-1.274	-2.418	0.00991	0.039215
TRIM66	-1.274	-2.418	0.005329	0.02418
ODF2	-1.273	-2.417	0.001414	0.008636
ZNF83	-1.273	-2.416	0.000984	0.006459
CERS6	-1.269	-2.41	0.009242	0.037097
NBPF10	-1.269	-2.41	0.001016	0.006622
ELF2	-1.263	-2.399	0.000885	0.005957
FAM57A	-1.262	-2.398	0.004503	0.021181
TTBK2	-1.262	-2.398	0.001662	0.009845
ZC3HAV1	-1.261	-2.397	0.002813	0.014813
MMP2	-1.26	-2.395	0.011549	0.043991

SRGN	-1.26	-2.395	0.006777	0.029176
VAMP1	-1.258	-2.391	0.010588	0.041184
RNF24	-1.258	-2.391	0.005174	0.023649
SMTN	-1.255	-2.387	0.010964	0.042279
ZNF577	-1.255	-2.386	0.0085	0.034851
SH3GLB2	-1.254	-2.385	0.003947	0.01919
SH3RF1	-1.254	-2.385	0.004855	0.022449
NPHP4	-1.251	-2.38	0.006272	0.027434
DDX58	-1.249	-2.377	0.006137	0.026981
ZBED6	-1.247	-2.374	0.006008	0.026552
BNC2	-1.247	-2.373	0.000821	0.005619
ARHGAP24	-1.245	-2.371	0.003652	0.018079
NA	-1.245	-2.371	0.000787	0.00545
EXT1	-1.245	-2.369	0.013645	0.049955
FAM102B	-1.243	-2.367	0.004485	0.021124
AFG3L1P	-1.241	-2.364	0.01006	0.0396
APPL2	-1.241	-2.363	0.00391	0.019071
GLTSCR1L	-1.24	-2.363	0.00166	0.009845
STXBP5	-1.24	-2.362	0.002756	0.014577
HSPA13	-1.239	-2.36	0.005534	0.02492
ATP1A1	-1.239	-2.36	0.007454	0.03149
KIAA1731	-1.237	-2.358	0.003674	0.018161
DEPTOR	-1.237	-2.357	0.008868	0.035971
GOLGA8R	-1.234	-2.352	0.012637	0.047064
CCDC149	-1.234	-2.352	0.009997	0.039408
MR1	-1.232	-2.349	0.001015	0.006619
SCPEP1	-1.232	-2.349	0.001869	0.010792
ARHGAP26	-1.229	-2.345	0.003749	0.018469
PAQR7	-1.226	-2.34	0.01191	0.044998
ENPP2	-1.226	-2.339	0.005894	0.02615
PPAPDC1B	-1.226	-2.339	0.007447	0.031481
LOC101929147	-1.22	-2.329	0.01315	0.048523
TRPM4	-1.219	-2.327	0.00291	0.015202
SLC11A2	-1.218	-2.326	0.001014	0.006618
ERVK13-1	-1.217	-2.324	0.003934	0.019148
MARVELD1	-1.215	-2.322	0.010436	0.040784
ZSCAN26	-1.214	-2.32	0.007731	0.032389
S100PBP	-1.214	-2.319	0.005737	0.025671
UGDH	-1.213	-2.318	0.002655	0.014171
PLCE1	-1.213	-2.318	0.0036	0.017891
LINC00968	-1.212	-2.317	0.008818	0.035819
SPTLC2	-1.211	-2.315	0.002042	0.011552
GOLGB1	-1.21	-2.313	0.009165	0.036898
PPP1R15A	-1.21	-2.313	0.00403	0.019485
PDLIM4	-1.207	-2.308	0.002683	0.014299
IRAK4	-1.206	-2.306	0.002755	0.014575
CEP95	-1.205	-2.306	0.003215	0.016404
PTPN9	-1.205	-2.306	0.001423	0.008677
LRP12	-1.205	-2.305	0.009085	0.036673
HERC2	-1.205	-2.305	0.009091	0.036687

ZNF84	-1.205	-2.305	0.002745	0.014548
PTPN14	-1.203	-2.303	0.006597	0.028552
DYNC2H1	-1.201	-2.299	0.001509	0.009117
TXNRD1	-1.2	-2.298	0.012453	0.046541
JMJD1C	-1.2	-2.297	0.004453	0.021012
FKBP9	-1.2	-2.297	0.012897	0.04783
NBPF15	-1.199	-2.296	0.011951	0.045099
RBL1	-1.198	-2.295	0.013631	0.049925
RBFOX2	-1.197	-2.293	0.010233	0.040142
SRSF11	-1.196	-2.292	0.009586	0.038231
PCDH18	-1.195	-2.289	0.013345	0.049133
PNRC1	-1.191	-2.284	0.007615	0.032023
SLC24A1	-1.191	-2.282	0.004313	0.020494
TYMP	-1.19	-2.282	0.008675	0.035431
TGFBR1	-1.19	-2.281	0.006335	0.027639
RBMS1	-1.189	-2.279	0.007466	0.031524
ZNF655	-1.187	-2.277	0.001548	0.009299
TBC1D8B	-1.187	-2.277	0.005308	0.024092
CHST14	-1.186	-2.275	0.003541	0.017669
CDK19	-1.186	-2.275	0.002825	0.014864
NLRP1	-1.186	-2.275	0.005351	0.024266
TMCO3	-1.185	-2.274	0.009163	0.036898
NFAT5	-1.185	-2.274	0.004364	0.020691
GADD45B	-1.185	-2.273	0.006753	0.029117
DNAJC18	-1.183	-2.271	0.008695	0.035499
TMEM64	-1.182	-2.268	0.013563	0.049734
NBPF11	-1.178	-2.262	0.004891	0.022577
TMEM198B	-1.177	-2.261	0.010544	0.041064
FKBP11	-1.176	-2.26	0.005843	0.025991
NDST2	-1.176	-2.26	0.003479	0.017474
RCN3	-1.173	-2.255	0.012033	0.045264
CEP290	-1.172	-2.253	0.01235	0.046234
ATM	-1.172	-2.253	0.004365	0.020691
ZNF395	-1.17	-2.251	0.00695	0.029733
TRIM38	-1.17	-2.25	0.002112	0.01187
ZCCHC8	-1.17	-2.249	0.009013	0.036427
UBN2	-1.169	-2.248	0.005044	0.023135
YPEL3	-1.169	-2.248	0.008523	0.034924
SNX25	-1.168	-2.247	0.008649	0.035343
RASA2	-1.167	-2.246	0.010866	0.041985
IL13RA1	-1.166	-2.244	0.005432	0.02454
VPS13C	-1.166	-2.243	0.005795	0.025852
MYO9B	-1.166	-2.243	0.006681	0.02887
YEATS2	-1.162	-2.238	0.006246	0.027364
ZNF160	-1.16	-2.235	0.002232	0.012404
RAB11FIP3	-1.16	-2.234	0.001897	0.010904
CLIP2	-1.157	-2.23	0.010453	0.040805
SLC30A7	-1.157	-2.23	0.002808	0.014794
H3F3B	-1.153	-2.224	0.011491	0.043837
PRKAB2	-1.152	-2.222	0.004302	0.020447

REXO2	-1.152	-2.222	0.006293	0.027508
TMF1	-1.149	-2.218	0.006076	0.026791
DOPEY1	-1.148	-2.216	0.002737	0.014513
FER	-1.145	-2.212	0.007807	0.032609
DNMBP	-1.145	-2.211	0.00252	0.013602
HEXB	-1.144	-2.21	0.009156	0.036893
ARSJ	-1.142	-2.208	0.013584	0.049797
MED13L	-1.138	-2.201	0.012016	0.045221
TRAK2	-1.136	-2.198	0.013537	0.049667
FNBP4	-1.135	-2.197	0.002837	0.014898
FSTL3	-1.133	-2.194	0.006802	0.029256
YPEL2	-1.131	-2.19	0.00303	0.015696
LARGE	-1.128	-2.186	0.006073	0.026787
TUBG2	-1.128	-2.186	0.008437	0.034667
EBF3	-1.126	-2.182	0.002903	0.015175
STON1	-1.122	-2.176	0.009929	0.039265
RALA	-1.122	-2.176	0.004815	0.022301
PDE4A	-1.12	-2.174	0.003339	0.016927
CREBZF	-1.118	-2.17	0.00405	0.019565
GAA	-1.116	-2.168	0.006122	0.026932
SNX29	-1.114	-2.164	0.002286	0.012597
CCDC93	-1.113	-2.163	0.005263	0.023946
SENP1	-1.113	-2.163	0.011223	0.04308
FAM43A	-1.11	-2.159	0.010025	0.039485
ZNF33A	-1.107	-2.154	0.003938	0.01915
PDP1	-1.105	-2.151	0.011866	0.04488
RNF217	-1.101	-2.146	0.013476	0.049469
MALT1	-1.1	-2.144	0.006928	0.029666
EP400	-1.1	-2.143	0.004489	0.021128
CNPY3	-1.099	-2.142	0.00677	0.029152
RNF130	-1.098	-2.141	0.005222	0.023813
EDEM1	-1.098	-2.141	0.010692	0.041489
NF1	-1.098	-2.14	0.010441	0.040794
PLEKHA4	-1.094	-2.135	0.013544	0.04968
ZNF224	-1.092	-2.132	0.009397	0.03762
EEF2K	-1.089	-2.127	0.005135	0.023494
KMT2E	-1.089	-2.127	0.007052	0.030122
CHD2	-1.087	-2.125	0.011892	0.04495
KIAA0907	-1.085	-2.121	0.004345	0.020608
FAM26E	-1.085	-2.121	0.004655	0.021701
GUSB	-1.085	-2.121	0.011502	0.043861
TTC14	-1.084	-2.12	0.006052	0.026702
CDC14B	-1.084	-2.12	0.002972	0.015462
ABI2	-1.082	-2.118	0.004555	0.021351
RECQL	-1.082	-2.118	0.009433	0.037742
ST5	-1.082	-2.117	0.006372	0.027778
CD55	-1.081	-2.116	0.012313	0.046132
PCGF5	-1.081	-2.116	0.009674	0.038512
TRIM25	-1.081	-2.115	0.010853	0.041956
SERPINB8	-1.074	-2.106	0.008416	0.034612

ZNF862	-1.071	-2.1	0.009272	0.03721
TSPAN4	-1.07	-2.099	0.003216	0.016404
SPECC1L	-1.067	-2.096	0.005769	0.025786
IFT57	-1.067	-2.095	0.004704	0.021861
ELF1	-1.065	-2.092	0.006995	0.0299
CD47	-1.062	-2.088	0.007115	0.030343
RAB34	-1.061	-2.087	0.004384	0.020758
CCDC82	-1.061	-2.086	0.007326	0.031059
MAPRE2	-1.056	-2.079	0.010073	0.039631
GNG11	-1.052	-2.074	0.005875	0.026094
PCGF2	-1.051	-2.072	0.007565	0.03185
CEP120	-1.051	-2.071	0.012506	0.046702
CERS5	-1.049	-2.069	0.007378	0.031247
CHD9	-1.048	-2.068	0.013527	0.049641
GRB10	-1.048	-2.068	0.007949	0.033132
BTN2A1	-1.047	-2.066	0.009189	0.036962
NFKB1	-1.046	-2.065	0.004938	0.022749
USP1	-1.046	-2.064	0.010302	0.040364
EZH1	-1.04	-2.056	0.004058	0.019577
GBA	-1.037	-2.052	0.010466	0.040845
ZNF652	-1.031	-2.043	0.00642	0.027948
C2CD5	-1.028	-2.04	0.007646	0.032108
MLXIP	-1.023	-2.033	0.010912	0.042124
UHRF2	-1.02	-2.027	0.008878	0.035989
CEP192	-1.015	-2.021	0.011745	0.044495
SNHG1	-1.014	-2.02	0.013104	0.048416
ZDHHC17	-1.009	-2.013	0.005221	0.023813
PCNXL4	-1.008	-2.012	0.009812	0.038893
NPHP3	-1.004	-2.005	0.01315	0.048523
POGZ	-1.003	-2.004	0.008976	0.036289
RNF103	-1.001	-2.001	0.006859	0.029455
LSM8	-1.001	-2.001	0.008455	0.034708
SREK1	-1.001	-2.001	0.011568	0.044008
TNKS	-1	-2	0.013449	0.049437
KIAA0556	-0.998	-1.998	0.009312	0.037312
ZHX2	-0.995	-1.994	0.009993	0.039406
PPWD1	-0.991	-1.988	0.010101	0.039705
ENDOD1	-0.991	-1.987	0.007397	0.03131
TSHZ1	-0.988	-1.983	0.007921	0.033053
HECA	-0.973	-1.963	0.008659	0.035375
SRC	-0.972	-1.962	0.01295	0.047967
TRRAP	-0.969	-1.957	0.012241	0.045928
MIER1	-0.965	-1.952	0.009934	0.039274
NLGN2	-0.962	-1.949	0.011432	0.043687
MLLT6	-0.961	-1.947	0.008244	0.034038
GALC	-0.96	-1.945	0.009676	0.038512
CDK5RAP2	-0.959	-1.943	0.009824	0.038928
DDX39B	-0.957	-1.941	0.012523	0.046729
PVRL3	-0.957	-1.941	0.013156	0.048531
TP53BP2	-0.947	-1.928	0.010743	0.041637

TBRG1	-0.945	-1.926	0.011536	0.043971
MAML2	-0.94	-1.918	0.01131	0.043355
MEIS2	-0.936	-1.913	0.011293	0.043312
RNF146	-0.932	-1.908	0.009455	0.037811

KEGG Term	Count	Upregulated/ downregulated set of genes	Genes	P-value (Bonferroni)
**Oxidative phosphorylation**	37	Upregulated	ATP5D, NDUFB4, NDUFB5, NDUFB6, NDUFB7, COX10, NDUFB9, CYC1, NDUFAB1, ATP5G2, ATP5G1, ATP6V1G1, UQCRFS1, UQCRQ, ATP5G3, COX5B, ATP6V0B, NDUFB2, NDUFS7, NDUFS6, NDUFS8, ATP5O, COX17, NDUFS2, ATP5J, NDUFA4, ATP5J2, NDUFA2, NDUFA3, COX8A, NDUFC2, ATP6V1D, NDUFV3, ATP6V0E2, UQCRH, SDHC, SDHD	1E-19
**Valine, leucine and isoleucine degradation**	24		ACAA2, HSD17B10, ALDH6A1, ACADSB, ACADM, BCAT2, ACADS, BCKDHB, HMGCS1, ACAT2, ALDH3A2, HADHA, HADHB, MCCC2, DBT, ALDH1B1, MCCC1, ALDH2, ACAD8, HIBCH, HADH, PCCB, ALDH9A1, ACAA1	2E-19
**Parkinson's disease**	31		ATP5D, NDUFB4, NDUFB5, NDUFB6, NDUFB7, NDUFB9, CYC1, NDUFAB1, ATP5G2, ATP5G1, UQCRFS1, UQCRQ, ATP5G3, COX5B, NDUFB2, NDUFS7, NDUFS6, NDUFS8, ATP5O, NDUFS2, ATP5J, NDUFA4, NDUFA2, NDUFA3, COX8A, CYCS, NDUFC2, NDUFV3, UQCRH, SDHC, SDHD	7E-14
**Alzheimer's disease**	32		ATP5D, NDUFB4, HSD17B10, NDUFB5, NDUFB6, NDUFB7, NDUFB9, CYC1, NDUFAB1, ATP5G2, ATP5G1, UQCRFS1, UQCRQ, ATP5G3, COX5B, NDUFB2, NDUFS7, NDUFS6, NDUFS8, ATP5O, NDUFS2, ATP5J, NDUFA4, NDUFA2, NDUFA3, COX8A, CYCS, NDUFC2, NDUFV3, UQCRH, SDHC, SDHD	1E-11
**Fatty acid metabolism**	17		ACAA2, ACOX1, GCDH, ACADSB, ACADM, ACADS, ACAT2, ALDH3A2, HADHA, HADHB, ACADVL, ACSL1, ALDH1B1, ALDH2, HADH, ALDH9A1, ACAA1	6E-11
**Huntington's disease**	32		ATP5D, NDUFB4, NDUFB5, POLR2E, NDUFB6, NDUFB7, NDUFB9, CYC1, NDUFAB1, ATP5G2, ATP5G1, UQCRFS1, UQCRQ, ATP5G3, COX5B, NDUFB2, NDUFS7, NDUFS6, NDUFS8, ATP5O, NDUFS2, ATP5J, NDUFA4, NDUFA2, NDUFA3, COX8A, CYCS, NDUFC2, NDUFV3, UQCRH, SDHC, SDHD	2E-10
**Glycerolipid metabolism**	15		DGKQ, PNPLA3, ALDH3A2, GLYCTK, DGAT1, DGKD, ALDH1B1, ALDH2, MGLL, DGKZ, GK, GPAM, AGK, ALDH9A1, AGPAT2	9E-08
**Butanoate metabolism**	13		ACADS, ALDH5A1, HMGCS1, ACAT2, ALDH3A2, HADHA, ACSM3, ALDH1B1, ALDH2, PDHA1, HADH, BDH1, ALDH9A1	3E-07
**Propanoate metabolism**	12		ALDH6A1, ACADM, MLYCD, ALDH1B1, ALDH2, HIBCH, ACSS2, ACAT2, PCCB, ALDH3A2, HADHA, ALDH9A1	2E-06
**Citrate cycle (TCA cycle)**	11		ACO1, SDHC, SDHD, CS, IDH1, ACLY, PDHA1, DLAT, IDH3A, PCK1, MDH1	2E-05
**Terpenoid backbone biosynthesis**	8		MVD, HMGCR, HMGCS1, FDPS, MVK, IDI1, PMVK, ACAT2	7E-05
**beta-Alanine metabolism**	9		ACADM, MLYCD, ALDH1B1, ALDH2, HIBCH, ALDH3A2, HADHA, ALDH9A1, AOC3	1E-04
**Glycerophospholipid metabolism**	14		CHKA, PLA2G4A, CRLS1, DGKQ, DGKD, PLA2G12A, PEMT, DGKZ, PISD, PCYT2, PNPLA3, GPAM, AGPAT2, PTDSS2	2E-04
**Glyoxylate and dicarboxylate metabolism**	7		MTHFD1, GLYCTK, PGP, ACO1, CS, MDH1, HYI	1E-03
**Pyruvate metabolism**	10		ALDH1B1, ALDH2, PDHA1, DLAT, ACSS2, ACAT2, ALDH3A2, ALDH9A1, PCK1, MDH1	2E-03
**Arginine and proline metabolism**	11		PYCRL, CKMT1A, CKMT1B, ACY1, ASS1, ALDH1B1, ALDH2, ALDH4A1, GAMT, AMD1, ALDH3A2, ALDH9A1	4E-03
**Glycolysis / Gluconeogenesis**	11		ALDOA, ALDH1B1, PGM1, ALDH2, ALDH3B2, PDHA1, DLAT, ACSS2, ALDH3A2, ALDH9A1, PCK1	1E-02
**Limonene and pinene degradation**	6		ALDH1B1, ALDH2, PNPLA3, ALDH3A2, HADHA, ALDH9A1	1E-02
**Fatty acid elongation in mitochondria**	5		ACAA2, HADH, MECR, HADHA, HADHB	2E-02
**O-Glycan biosynthesis**	7	Downregulated	GALNT10, GALNT7, WBSCR17, GALNT5, GALNT12, GCNT1, GALNT13	4E-04
**Sphingolipid metabolism**	6		SPTLC2, SPHK1, GALC, B4GALT6, PPAP2B, GBA	2E-02
**Nicotinate and nicotinamide metabolism**	5		NMNAT2, BST1, NT5C2, NT5E, NNMT	4E-02
**Drug metabolism**	6		CYP3A5, TYMP, GUSB, UPP1, CDA, TK1	4E-02
